# Evaluation of the risk factors for venous thromboembolism post splenectomy – A ten year retrospective cohort study in St James’s hospital

**DOI:** 10.1016/j.amsu.2021.102381

**Published:** 2021-05-08

**Authors:** Manal Abduljalil, Jean Saunders, Dearbhla Doherty, Marthinus Dicks, Catherine Maher, Brian Mehigan, Richard Flavin, Catherine M. Flynn

**Affiliations:** a Department of Haematology, St James's Hospital, James Street, Dublin, D03 R2WY, Ireland; b Director of Centre for Support Training Analysis Research, University of Limerick, Limerick, Ireland; c HOPE Directorate, St James's Hospital, James Street, Dublin, D03 R2WY, Ireland; d Department of Surgery, St James's Hospital, James Street, Dublin, D03 R2WY, Ireland; e Department of Histology, St James's Hospital, James's Street, Dublin, D03 R2WY, Ireland

**Keywords:** Splenectomy, Venous thromboembolism, Obesity, Prophylactic anticoagulation

## Abstract

**Background:**

Splenectomy is a surgical intervention for a variety of indications; benign and malignant. Complications of this procedure include Venous thromboembolism (VTE) and infection. The incidence of VTE post-surgery has been reported between 0.8%–3% depending on the type of surgery. A higher incidence of abdominal VTE was reported post splenectomy (6–11%). However, there is limited literature regarding the risk factors for post splenectomy VTE and the optimal strategy for thromboprophylaxis.

**Objective:**

The primary objective of the study was to evaluate the incidence of VTE post splenectomy and to identify the pre-operative, intra-operative and post-operative risk factors. The secondary objective was to assess the local compliance with post-splenectomy prophylactic antibiotics and vaccination protocols.

**Methods:**

We conducted a retrospective observational study. All patients who had a splenectomy in St James's Hospital between January 2007 and June 2017 were included and reviewed. Statistical analysis was carried out using SPSS statistical package.

**Results:**

85 patients were involved in the study. The main indications for splenectomy were benign haematology, malignant haematology, solid tumours, traumatic and spontaneous rupture. 6/85 patients developed VTE (7.06%).

High BMI ≥ 30 was associated with increased risk of VTE (p = 0.007), while the use of post-operative prophylactic anticoagulation was associated with reduced risk (p = 0.005). Other factors including age >50 years, female gender, presence of active malignancy and splenomegaly were associated with increased VTE risk with no statistical significance. All VTE's occurred in elective versus emergency splenectomy. Laparoscopic splenectomy was associated with higher risk of VTE than open splenectomy. 97% of patients were prescribed prophylactic antibiotics on discharge, but only 88% had received recommended vaccinations.

**Conclusion:**

Venous thromboembolism is common post splenectomy. Our data showed that BMI ≥30 was associated with a statistically significant increased risk of VTE, while the use of prophylactic anticoagulation was associated with reduced risk. Further prospective studies with larger samples are warranted and a splenectomy care plan may be helpful.

## Highlights

• Venous thromboembolism is common post splenectomy procedure.
• High Body Mass Index can increase the risk of venous thromboembolism post splenectomy surgery.
• The use of extended prophylactic anticoagulation post splenectomy can reduce the venous thromboembolism complications.
• Many other factors can contribute to the venous thrombosis risk post splenectomy at variable extents.

## 1 Introduction

Once considered a vestigial organ, the spleen has important haematological and immunological functions. These include removing old and damaged red blood cells from the circulation, direct clearance of pathogens from the bloodstream and generation of immune responses to pathogens.

Splenectomy remains a diagnostic and therapeutic option in a variety of clinical circumstances. Therapeutic indications for splenectomy include benign haematological conditions such as refractory idiopathic thrombocytopenic purpura (ITP), warm autoimmune haemolytic anaemia (AIHA) and severe inherited haemolytic anaemias, such as hereditary spherocytosis. Splenectomy is also considered a treatment option for malignant conditions including lymphoma [1], chronic lymphoblastic leukaemia (CLL) and solid tumours with local invasion to the spleen. Other less common indications for removal include hypersplenism in thalassemia patients, traumatic or spontaneous splenic rupture, parenchymal splenic lesion, splenic abscess and splenic artery aneurysm.

The spleen can be removed either via classical open laparotomy or laparoscopic surgical approaches. While postoperative morbidity and mortality outcomes are more favourable with laparoscopic approaches due to minimal surgical trauma and faster recovery [2,3], both approaches can be associated with serious complications. These include intra and postoperative haemorrhage, infections, malignancy, and thromboembolism.

Infection risk is greatest in the early postoperative period, with an estimated 10% of splenectomies complicated by infection during the first 90 days [4]. The lifetime risk of overwhelming post-splenectomy infection is approximately 1–3% [5], with patients susceptible to infection by encapsulated organisms causing pneumococcal pneumonia (RR 2.1), meningitis (RR 2.4), and septicaemia (RR 3.4) [6]. Post-splenectomy infections may be fatal, particularly in younger, immunocompromised patients or those with malignant disease [6]. The increase risk of infections post splenectomy is attributed to the loss of the protective immunological function of the spleen. This risk of infection justifies the importance of vaccination and the use of prophylactic antibiotics post splenectomy.

Splenectomized patients have an increased risk of cancer, as well as increased cancer related mortality (RR 1.5 for both). A variety of cancer types have been reported with increased incidence post splenectomy, including solid neoplasms (lung, prostate, upper gastrointestinal and hepatic malignancies, RR 1.3–1.9); and haematologic malignancies (non-Hodgkin lymphoma, leukaemia & multiple myeloma, RR 1.8–6.0). Based on sparse data from animal studies, it has been suggested that splenectomy can induce of enhance tumour growth [7,8]. The risk for cancer development is greatest during the first two to five years post-splenectomy. However, a persistent risk beyond ten years post-splenectomy was demonstrated in one study [6].

Venous thromboembolism (VTE) is an infrequent but serious complication after splenectomy. Over the last two decades, there has been increased awareness about the risk of thromboembolism in the post-operative period, and post splenectomy in particular. There have been multiple studies to assess the incidence of venous thromboembolism post splenectomy. In a large cohort study of more than 8000 patients, splenectomized patients had an increased risk of developing deep vein thrombosis (DVT) (RR = 2.18) and pulmonary embolism (RR = 2.24), but not coronary artery disease, or ischaemic stroke [6].

In another cohort study with a similar population, VTE occurred in 1.9% of splenectomy patients within the ﬁrst 90 days post splenectomy in contrast to 0.1% of general population controls [9].

In a third large cohort involving more than 1.5 million patients, the incidence of symptomatic venous thromboembolic events (VTE) was determined within a 3-month period after 76 different surgical procedures. Among 3833 patients post splenectomy, the incidence of VTE was 1.6%, compared with 0.8% in the general surgical population. The majority of VTE events (56%) were diagnosed post hospital discharge [10].

A higher incidence of post-splenectomy portal vein system thrombosis (PVST) in noncirrhotic patients was reported, at 6%–11% [2].

The increased risk of VTE appears to be due to a hypercoagulable state post splenectomy. Many mechanisms have been proposed including the transient thrombocytosis and leucocytosis secondary to absent splenic breakdown, platelet agglutination, increased rigidity of erythrocytes and disturbance and activation of vascular endothelium. In addition, changes in postoperative haemodynamics and the decrease in portal vein blood flow velocity were attributed to the development of postoperative PVST [6,11].

Multiple risk factors for post splenectomy VTE have been reported in the literature. Patient specific risk factors including advanced age [3] and active malignancy [9], procedure specific factors including laparoscopic surgery [2,3] and thromboprophylaxis [2,11,12] and haematological factors including platelet count ≥ 650 × 10^9^/L [13] or < 50 × 10^9^/L [14] and D-dimer level of >500 lg/mL [13].

Additional risk factors have been identified among specific patient subgroups. For example, portal vein diameter >13 mm and enlarged splenic vein diameter were associated with increased risk of PVST after Laparoscopic Splenectomy in cirrhotic patients with hypersplenism due to portal hypertension [3]. Among thalassemia patients, spleen weight of more than 1.5 kg was the only independent factor associated with the presence of PVST [15].

## 2 Objectives

The primary objective of the study was to evaluate the incidence of VTE post splenectomy and to identify the pre-operative, intra-operative and post-operative risk factors.

The secondary objective was to assess local compliance with post-splenectomy prophylactic antibiotics and vaccination protocols.

## 3 Methods & materials

### 3.1 Patients

The study was conducted in St James's Hospital Dublin. All cases of splenectomy in St James's Hospital between January 2007 and June 2017 were identified by review of splenic specimens processed in St James's Hospital histopathology laboratory during this time period. Splenic biopsy samples and external samples were excluded. Of the 98 cases of splenectomy identified, incomplete data was available in 8 patients and 5 patients were on therapeutic anticoagulation for VTE prior to the surgery; these were excluded from the analysis. The remaining 85 patients were included in the cohort.

### 3.2 Clinical data

We performed a retrospective observational analysis. Data was obtained from both paper and electronic patient records. All patients who developed VTE were identified by confirmed venous thromboembolism radiologically post splenectomy. Details of the timing of VTE post splenectomy, vessels involved, and platelet count at the time of the event were collected. Multiple perioperative factors were studied. Preoperative factors included patients’ age, gender, BMI, comorbid illness using Charlson Comorbidity Index, presence of malignancy, use of hormonal contraception, spleen size on imaging, preoperative platelet (PLT) count, preoperative use of anticoagulation or anti-platelets, indication and urgency of procedure. Intraoperative factors included type of surgery, open or laparoscopic, complexity of the procedure, duration of anaesthesia, estimated blood loss and blood product transfusion. Postoperative factors included length of hospital stay, wound complications and additional interventions, postoperative platelet count and use of anticoagulation or anti-platelets during admission and upon discharge. Histological data including splenic dimensions, weight and underlying histological diagnosis were also recorded. As a secondary aim, use of perioperative vaccination and postoperative prophylactic antibiotics was recorded.

### 3.3 Statistical analyses

Statistical analysis of the data was done using SPSS statistical package. The inﬂuence of possible risk factors in the development of VTE was assessed by univariate and multivariate analyses. The demographic and clinical characteristics of the patients who developed VTE post-splenectomy and those who did not were compared and percentages, means (SDs), medians, IQRs given in each group where appropriate. Cross-tabulations and chi-squared and fishers exact tests (FET) were carried out to see was analyse any association between the factor and VTE incidence. The work has been reported in line with the STROCSS criteria [38].

## 4 Results

**Fig. 1:**
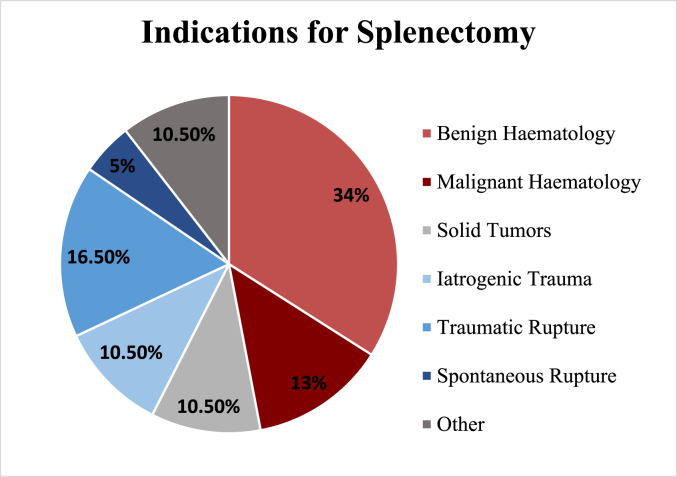
Indications for splenectomy.

A total of 85 patients who underwent splenectomy surgery were included in the study. The median age at time of the splenectomy was 48 years with a male predominance (51.8 versus 48.2%) The indications for splenectomy were benign haematology (n = 29), malignant haematology (n = 11), solid tumours (n = 9), traumatic rupture (n = 14), iatrogenic trauma (n = 9), spontaneous rupture (n = 4) and others (n = 9). Details on the indications for splenectomy are demonstrated in Fig. 1 and Table 1.

**Table 1:** Detailed indications for splenectomy.

Indications for Splenectomy	
**Indication**	**No. of Patients**

**Benign haematology:**	
ITP	21
AIHA	4
Hereditary spherocytosis	4
Total	29

**Malignant haematology**	
Lymphoma	9
CLL	2
Total	11

**Solid tumours**	
Gastric cancer	3
Invasive colonic cancer	1
Metastatic ovarian cancer	1
Metastatic cervical cancer	1
Melanoma	1
Metastatic adenocarcinoma unknown primary	2
Total	9

**Traumatic splenic rupture**	14
**Iatrogenic splenic trauma**	9
**Spontaneous splenic rupture**	4
**Others:**	
Ruptured splenic artery aneurysm	2
AAA repair	2
Splenic AV fistula repair	1
Calcified splenic cyst	1
Splenic abscess	1
Pancreaticodudenal fistula	1
Distal Pancreatic lesion	1
Total	9

Of the 85 patients included in the analysis, 6 patients developed VTE complications within 90 days post splenectomy (VTE incidence rate 7.06%).

Among the 6 VTE events, 5 (83.3%) were symptomatic. These consisted of four splanchnic venous thromboses (2 in the splenic vein, 1 in the portal vein and 1 in the portal and splenic veins diagnosed by abdominal CT scan on days 6, 23, 16 and 9 respectively) and one non-fatal pulmonary embolism diagnosed by pulmonary CT angiogram on day 5. The asymptomatic event involved multiple upper and lower limb venous thromboses (including internal jugular, common femoral and common iliac veins) diagnosed incidentally by CT scan for restaging of malignancy on day 28 postoperatively.

The median interval between splenectomy procedure and the VTE detection in our study was 16 days. The mean platelet count at the time of VTE events was 624 × 10^3^ (range 204 × 10^9^/L to 1414 × 10^9^/L). All but one VTE event occurred after the patients were discharged from the hospital. Details of patients’ characteristics and VTE diagnosis are presented in Table 2.

**Table 2:** Details of patients with VTE.

Age	Gender	BMI	Active cancer	Indication for surgery	Type of surgery	Procedure	POD of VTE diagnosis	Presence of symptoms	VTE location	PLT count on diagnosis
70	F	30.8	Yes	Iatrogenic rupture	Elective	Laparoscopic	28	No	RIJ, RCF, RCI veins	444
62	F	25.57	No	AV fistula of splenic artery	Elective	Open	6	Yes	Splenic vein	842
42	F	48	No	Refractory ITP	Elective	Open	5	Yes	Pulmonary artery	204
67	M	23.38	Yes	B-Cell lymphoma	Elective	Open	23	Yes	PSVT	214
38	F	34.91	No	Refractory ITP	Elective	Laparoscopic	9	Yes	PSVT	628
19	F	N/A	No	Hereditary spherocytosis	Elective	Laparoscopic	16	Yes	PSVT	1414

BMI, body mass index; POD, postoperative day; VTE, venous thromboembolism; PLT, platelets; ITP, immune thrombocytopenic purpura; RIJ, right internal jugular; RCF, right common femoral; RCI, right common iliac; PSVT Portal-splenic venous thrombosis, N/A, not available.

### 4.1 Pre-operative risks analysis

Body Mass Index (BMI) was documented for 35 out of 85 patients. High BMI ≥30 was significantly associated with increased risk of VTE (p = 0.007). 60% (3/5) of patients in the VTE group had high BMI ≥ 30 in comparison to 20% (6/29) of patients in the non-VTE group. Female gender was not associated with increased VTE risk (p = 0.102). All patients who developed VTE underwent splenectomy in the elective setting. Age ≥50 (p = 0.58), active cancer (p = 1.000), high preoperative platelets > 450 × 10^9^/L (p = 0.364), pre-operative vaccinations (p = 0.233) and splenomegaly (defined by longitudinal diameter of spleen > 12 cm in imaging and/or splenic gross weight > 500 g) (p = 0.157) were associated with higher incidence of VTE with no statistical significance. No signiﬁcant differences were found in terms of the use of oral contraceptive pills (OCP) (p = 1.000), presence of documented chronic liver disease (p = 1.000), low preoperative platelet count < 100 × 10^9^/L (p = 0.576), indication for surgery (p = 0.851), emergency procedure (p = 0.086) and Charlson Comorbidity Score ≥ 3 (p = 1.000). Neither the use of preoperative antiplatelets medications (p = 0.576) or prophylactic anticoagulation (p = 1.000) altered the risk of postoperative VTE (Table 3).

**Table 3:** Demographic and perioperative clinical characteristics in the VTE and non-VTE groups.

Variable	VTE (n = 6)	Non-VTE (n = 79)	p Value
***Preoperative variable***			

Age ≥ 50	3/6 (50%)	36/79 (45.6%)	1.000
Female gender	5/6 (83.3%)	36/79 (35.6%)	0.102
BMI ≥ 30^a^	3/5 (60%)	6/29 (20%)	0.007
OCP^b^	0/5 (0%)	3/36 (8%)	1.000
Active cancer	2/6 (33.3%)	24/79 (30.4%)	1.000
CLD	0/6 (0%)	2/79 (2.5%)	1.000
Pre-operative PLT < 100 × 10^3^	1/6 (16.7%)	10/79 (12.7%)	0.576
Pre- operative PLT > 450 × 10^3^	1/6 (16.7%)	5/79 (6.3%)	0.364
Pre- operative antiplatelets	1/6 (16.7%)	10/79 (12.7%)	0.576
Pre- operative anticoagulation	1/6 (16.7%)	14/79 (17.7%)	1.000
Pre- operative vaccination	4/6 (66.7%)	28/70 (40%)	0.233
Emergency surgery	0/6 (0%)	30/79 (38%)	0.086
Splenomegaly^c^	3/6 (50%)	18/79 (22.8%)	0.157
Charlson comorbidity score ≥ 3	2/6 (33.3%)	27/79 (34.2%)	1.000
***Intraoperative variable***			

Laparoscopic surgery	3/6 (50%)	33/79 (41.8%)	0.695
Complex surgery	2/6 (33.3%)	25/79 (31.7%)	1.000
Red cell transfusion	1/6 (16.7%)	33/79 (41.8%)	0.395
Platelets transfusion	0/6 (0%)	15/79 (19.0%)	0.585
Plasma transfusion	0/6 (0%)	14/79 (17.7%)	0.583
Fibrinogen transfusion	0/6 (0%)	3/79 (3.8%)	1.000
Mean duration of surgery (hr)	2.87	2.87	0.547
Mean intra-operative blood loss (ml)	300	575	0.205

***Postoperative variable***			

Mean duration of hospital stay (day)	9.5	11	0.419
Malignant splenic pathology	1/6 (16.7%)	12/79 (15.2%)	1.000
Post-operative in hospital prophylactic anticoagulation	5/6 (83.3%)	62/79 (78.5%)	1.000
Wound complications	1/6 (16.7%)	18/79 (22.8%)	1.000
Intra-abdominal collection drainage/re-operation	2/6 (33.3%)	13/79 (16.5%)	0.285
Discharge on anticoagulation	0/4 (0%)	8/79 (10.1%)	0.005
Discharge on antiplatelets^d^	1/6 (16.7%)	16/79 (20.3%)	1.000
Post- operative PLT > 450 × 10^3^	4/6 (66.7%)	58/79 (73.4%)	0.660
Post- operative PLT > 1000 × 10^3^	1/6 (16.7%)	20/79 (25.3%)	1.000

BMI, body mass index; OCP, oral contraceptive pills; CLD, chronic liver disease; PLT, platelets.

a BMI was only documented for total of 34 patients (n = 34).

b OCP analysis was done among females (n = 41).

c Splenomegaly defined by longitudinal diameter of the spleen >12 cm in imaging and/or splenic gross weight of >500 g.

d Two patients developed VTE during admission and were discharged on therapeutic anticoagulation, so excluded from the analysis.

### 4.2 Intraoperative risks analysis

Intraoperative univariate analysis (Table 3) showed no difference in VTE risk between laparoscopic and open splenectomy (p = 0.69). VTE events complicated both procedures equally. The complexity of the surgery (p = 1.000) and the intraoperative red cell transfusion (p = 0.395) as well as transfusion of other blood components were not associated with increased risk of VTE with no significant inter-group difference was observed. The mean duration of surgery (p = 0.547) as well as the mean blood loss (p = 0.205) were similar in the VTE and non-VTE groups.

### 4.3 Postoperative risks analysis

The use of prophylactic anticoagulation post discharge was the only postoperative risk factor associated with significantly lower VTE risk (Table 3). 2 patients developed VTE events during hospital admission and were discharged on therapeutic anticoagulation, so were excluded from the analysis. None of the remaining 4 patients in the VTE group (0/4) had received prophylactic anticoagulation upon discharge, in comparison to 8/79 in the non-VTE group (p = 0.005). Mean duration of hospitalization was similar in VTE and non-VTE groups, 9.5 and 11 days respectively with no statistical significance (p = 0.419). No other postoperative variables significantly predicted VTE risk, including; malignant pathology of the spleen (p = 1.000), use of prophylactic anticoagulation during hospital stay (p = 1.000), wound infection (p = 1.000), intra-abdominal collection drainage or re-operation (p = 0.285), discharge on antiplatelets (p = 1.000) and highest recorded postoperative platelet count of >450 × 10^9^/L and >1000 × 10^9^/L within four weeks post-surgery (p = 0.660 and 1.000 respectively).

When multiple logistic regression analysis was conducted in a stepwise method, high BMI ≥30 and the use of prophylactic anticoagulation after discharge are the only signiﬁcant independent risk factors for VTE.

Demographic and perioperative clinical characteristics in the VTE and non-VTE groups are shown in Table 3.

Perioperative vaccinations and prophylactic antibiotics:

Most patients (89.4%) included in the analysis received perioperative vaccinations. 32 patients (37.6%) received preoperative vaccinations and 44 patients (51.8%) received postoperative vaccinations. However, vaccinations were not documented for 9/85 patients (10.6%). All except 2 patients (98%) were prescribed prophylactic antibiotics upon discharge.

## 5 Discussion

Splenectomy remains a therapeutic option for a variety of clinical conditions; benign and malignant. One third of patients in our cohort (34%) underwent splenectomy due to an underlying benign haematological condition. However, recently due to the introduction of alternative novel medical treatment options, fewer patients will be considered for surgical splenectomy.

In this study, the incidence of VTE post splenectomy was 7.05%. The majority of the VTE events were intraabdominal with a PVST incidence rate of 4.7%. These incidence rates are higher than the 0.63% VTE risk seen in previous large study of more than 180,000 patients who underwent vascular and general surgical procedures [16].

The reported incidence of PVST after splenectomy is variable. Lower incidence rates of 1.6% and 2% reported in two retrospective American and Dutch studies respectively [17,18]. Higher incidence rates have been reported in patients with B-thalassemia (8.3%), hereditary haemolytic anaemias (12.3%) [15], haematological malignancies (7.2%) [19], noncirrhotic patients (6–11%) [3] and cirrhotic patients with hypersplenism (24–40%) [3,14].

In addition, cases of VTE are often asymptomatic and are only detected on pre-emptive screening. 8–37% of all splenectomized patients found to have PVST in three studies in which patients were screened for PVST by doppler ultrasound [20,21] or contrast enhanced computed tomography (CT) scans [22], of which only 2–15% were symptomatic [9]. The variable incidence rates can be related to different sample sizes, the diagnostic modalities used and whether the patients were diagnosed upon screening or when symptomatic. Despite this variation, most of these studies suggested that splenectomy is associated with a significant risk of venous thromboembolism and pre-emptive monitoring for PVST by regular patient examination or screening imaging may ensure timely diagnosis and treatment to avoid further life-threatening complications [3,14,23].

In our study, all VTE events were detected between 5 and 28 days post splenectomy which probably indicates a higher thrombosis risk within the first 4 weeks post-surgery and the need for heightened vigilance and consideration of this severe complication. However, a persistent thrombosis risk more than ten years following the procedure was shown in one study suggesting a life-long susceptibility state [6].

Analysis of preoperative risk factors showed that BMI ≥30 was an independent risk factor for VTE development. Obesity is known as a risk factor for venous thrombosis [24]. It has been suggested that obesity is associated with inflammation and reduction in fibrinolysis with consequent increased thrombotic risk. There is a high incidence of obesity in the Irish population (60%) as revealed by HSE stats in April 2018. Predictions from UK Foresight Model indicate that 90% of Irish population will be overweight or obese by 2030.

The presence of active cancer has been shown to be associated with increased VTE risk after various general and vascular surgeries [10,16], as well as post splenectomy [9]. However, this association was not found in our study. Indeed, 50% of cases of VTE in our cohort occurred in patients with underlying ‘low risk’ indications, such as autoimmune disease and traumatic splenectomy. It was initially proposed that the rationale behind that was that some patients with underlying malignancy were discharged home on extended prophylaxis which altered their risk of VTE development. However, multivariate analysis excluded prophylaxis as a confounding factor.

In a study done by Rogers et al. emergency surgery was found to be associated with increased risk of VTE after general and vascular procedures [16]. However, this correlation was not demonstrated in our study as all VTE events occurred after elective procedures. Although found to be independent predictors for VTE in multiple previous studies, advanced age [3,10,25], female gender [16,24] and splenomegaly [9,15] were not associated with increased VTE risk in our cohort.

Worthy to mention that degree of portal hypertension manifested by wider splenic vein [26] and portal vein diameters [2] were attributed to higher risk of PVST post splenectomy in previous studies, these parameters were not assessed in our study. In addition, history of prior VTE was reported to have a strong association with VTE post cancer surgeries [25,27] and other different surgical procedures [10], however, this was not evaluated in our study as none of the VTE patients had prior VTE event.

In two previous studies, a higher rate of PVST was demonstrated post laparoscopic splenectomy with or without azygoportal disconnection in patients with liver cirrhosis and hypersplenism. It was suggested that the higher incidence of PVST in patients who have undergone laparoscopic procedure may be associated with CO2 pneumoperitoneum causing a hypercoagulable state and the technique and the instruments used to disconnect the splenic hilar vessels [2,3]. Yet, no difference in the incidence of PVST between open surgery and laparoscopic surgery was shown in one study [28]. Likewise, in our study both procedures were associated with similar VTE risk. While duration of anaesthesia of ≥3.5 h and >2 h strongly predicted the occurrence of postoperative VTE after orthopaedic and cancer surgeries respectively [2,25], this was not confirmed in our study. Although several studies described blood transfusions as a risk factor for postoperative VTE [3,16,[29], [30], [31]]; however, neither intraoperative blood transfusion nor intraoperative blood loss were associated with an increased VTE risk in this cohort.

In our study extended prophylactic anticoagulation on discharge was a significant negative predictor of VTE (*p* = 0.005). The effectiveness and safety of prophylactic anticoagulation use for postoperative VTE prevention has been explored and confirmed in several different settings, including surgery for malignant disease [27,32,33], orthopaedic surgery [34,35], and splenectomy in cirrhotic patients. In a study by Lai et al. involving more than 300 patients, early use of LMWH for 5 days post-surgery followed by a long-term maintenance therapy with warfarin and aspirin for one month was safe and effective method for PVST prevention after splenectomy with gastroesophageal devascularization [11]. In another retrospective cohort study of 75 consecutive cirrhotic patients who underwent laparoscopic splenectomy and azygoportal disconnection, the incidence of PVST was significantly lower in patient received postoperative thromboprophylaxis with warfarin than aspirin [2]. Another study reported that low molecular weight heparin (LMWH) was also effective in reducing the incidence of PVST in the same group [12]. High platelet count was associated with the development of PVST in previous studies [9,13,15], for which long-term antiplatelet therapy for high-risk patients has been recommended [15,36,37]. Yet, this was not confirmed for the patient population described here. Wound infection has been reported among fifteen variables independently associated with increased risk of VTE after general and vascular surgery [2]; however, this correlation was not shown in our cohort. Early ambulation was described as a significant protective factor of VTE post total hip arthroplasty [24], while prolonged bed rest was associated with a higher VTE risk post cancer surgery [25], those were difficult to be assessed in our study retrospectively.

The limitations of our study were its retrospective design and sample size We lacked complete data, including patient's body mass index, and details on postoperative immobility. Despite these limitations, a large number of perioperative risk factors have been assessed in the current study.

In conclusion, our study showed a high incidence of venous thromboembolism in patients post splenectomy, the majority of VTE involved the portal venous system. Patients require close follow-up for the development of PVST. Algorithms for investigation of abdominal pain and fever post splenectomy should consider PVST high in the differential. High BMI ≥30 was a significant positive predictor of VTE, while the use of extended prophylactic anti-coagulation was a significant negative predictor. To further reduce the incidence of symptomatic thromboembolism, efforts to improve recording of BMI on all patients and accurate in-hospital and post discharge thromboprophylaxis especially in overweight patients would be beneficial. Further prospective studies and large randomized controlled trials to confirm these findings are warranted. A splenectomy care plan to consider individual risks may be helpful. Compliance with peri-operative vaccinations and prophylactic antibiotic use was good but ongoing education of staff and patients to maintain and improve practice is recommended.

## Ethical approval

This study was performed as a retrospective audit and was given local authority to proceed through our hospital research and innovation office. Formal Ethical approval was not sought given the retrospective nature of the data review.

## Sources of funding

None.

## Author contribution

Dr Manal Abduljalil: Study design, data collection and writing the paper.

Dr Jean Saunders: Data analysis.

Dr Dearbhla Doherty: Data collection.

Dr Marthinus Dicks: Data collection.

Catherine Maher: Data analysis.

Mr Brian Mehigan: Study design and paper review.

Dr Richard Flavin: Data collection, paper review.

Dr Catherine M Flynn: Study design, paper review and editing.

## Research registration Unique Identifying number (UIN)

1. Name of the registry: https://www.researchregistry.com/
2. Unique Identifying number or registration ID: researchregistry6463
3. Hyperlink to your specific registration (must be publicly accessible and will be checked):https://emea01.safelinks.protection.outlook.com/?url=https%3A%2F%2Fwww.researchregistry.com%2Fbrowse-the-registry%23home%2F&data=04%7C01%7C%7Cea806e94e8094ce7d4e608d8ba4300e3%7C84df9e7fe9f640afb435aaaaaaaaaaaa%7C1%7C0%7C637464144219950422%7CUnknown%7CTWFpbGZsb3d8eyJWIjoiMC4wLjAwMDAiLCJQIjoiV2luMzIiLCJBTiI6Ik1haWwiLCJXVCI6Mn0%3D%7C1000&sdata=0sDas%2FQas2A33Jez8rVSzLS45aptBJMj3zZ9aK87RiE%3D&reserved=0

## Guarantor

Dr Manal Abduljalil, Clinical Haematologist and Internist, BDF Hospital, Bahrain.

Dr Catherine M Flynn, Consultant haematologist, St James's Hospital, James's Street, Dublin, Ireland. MCRN 22706.

## Declaration of competing interest

None.
