# Supplementary material for: Evaluation of the risk factors for venous thromboembolism post splenectomy – A ten year retrospective cohort study in St James’s hospital
Source: Ann Med Surg (Lond). 2021 May 8;66:102381. doi: 10.1016/j.amsu.2021.102381 (PMC8131975; doi:10.1016/j.amsu.2021.102381)
Supplement: Multimedia component 2 [file mmc2.docx]

**Crosstabs**

| **Case Processing Summary** | | | | | | |
| --- | --- | --- | --- | --- | --- | --- |
|  | Cases | | | | | |
|  | Valid | | Missing | | Total | |
|  | N | Percent | N | Percent | N | Percent |
| VTE * Age | 85 | 100.0% | 0 | 0.0% | 85 | 100.0% |

| **VTE * Age Crosstabulation** | | | | | | | | |
| --- | --- | --- | --- | --- | --- | --- | --- | --- |
|  | | | Age | | | | | Total |
|  |  |  | <18 | 18-29 | 30-49 | 50-65 | >65 |  |
| VTE | yes | Count | 0 | 1 | 2 | 1 | 2 | 6 |
|  |  | Expected Count | .1 | 1.6 | 1.5 | 1.6 | 1.1 | 6.0 |
|  |  | % within Age | 0.0% | 4.3% | 9.5% | 4.3% | 12.5% | 7.1% |
|  | no | Count | 2 | 22 | 19 | 22 | 14 | 79 |
|  |  | Expected Count | 1.9 | 21.4 | 19.5 | 21.4 | 14.9 | 79.0 |
|  |  | % within Age | 100.0% | 95.7% | 90.5% | 95.7% | 87.5% | 92.9% |
| Total | | Count | 2 | 23 | 21 | 23 | 16 | 85 |
|  |  | Expected Count | 2.0 | 23.0 | 21.0 | 23.0 | 16.0 | 85.0 |
|  |  | % within Age | 100.0% | 100.0% | 100.0% | 100.0% | 100.0% | 100.0% |

| **Chi-Square Tests** | | | |
| --- | --- | --- | --- |
|  | Value | df | Asymp. Sig. (2-sided) |
| Pearson Chi-Square | 1.584^a^ | 4 | .812 |
| Likelihood Ratio | 1.658 | 4 | .798 |
| Linear-by-Linear Association | .567 | 1 | .451 |
| N of Valid Cases | 85 |  |  |
| a. 6 cells (60.0%) have expected count less than 5. The minimum expected count is .14. | | | |

| **Symmetric Measures** | | | |
| --- | --- | --- | --- |
|  | | Value | Approx. Sig. |
| Nominal by Nominal | Phi | .137 | .812 |
|  | Cramer's V | .137 | .812 |
| N of Valid Cases | | 85 |  |


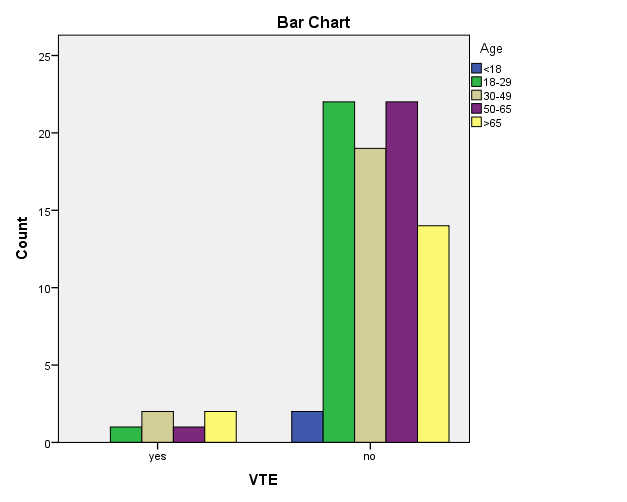


**Crosstabs**

| **Case Processing Summary** | | | | | | | | | | | | | | | | | |  |
| --- | --- | --- | --- | --- | --- | --- | --- | --- | --- | --- | --- | --- | --- | --- | --- | --- | --- | --- |
|  | | Cases | | | | | | | | | | | | | | | |  |
|  |  | Valid | | | | | Missing | | | | | | Total | | | | |  |
|  |  | N | | Percent | | | N | | | Percent | | | N | | | Percent | |  |
| VTE * Gender | | 85 | | 100.0% | | | 0 | | | 0.0% | | | 85 | | | 100.0% | |  |
| **VTE * Gender Crosstabulation** | | | | | | | | | | | | | | |  |  |  |  |
|  | | | | | | Gender | | | | | | Total | | |  |  |  |  |
|  |  |  |  |  |  | male | | | female | | |  |  |  |  |  |  |  |
| VTE | yes | | Count | | | 1 | | | 5 | | | 6 | | |  |  |  |  |
|  |  |  | Expected Count | | | 3.1 | | | 2.9 | | | 6.0 | | |  |  |  |  |
|  |  |  | % within Gender | | | 2.3% | | | 12.2% | | | 7.1% | | |  |  |  |  |
|  | no | | Count | | | 43 | | | 36 | | | 79 | | |  |  |  |  |
|  |  |  | Expected Count | | | 40.9 | | | 38.1 | | | 79.0 | | |  |  |  |  |
|  |  |  | % within Gender | | | 97.7% | | | 87.8% | | | 92.9% | | |  |  |  |  |
| Total | | | Count | | | 44 | | | 41 | | | 85 | | |  |  |  |  |
|  |  |  | Expected Count | | | 44.0 | | | 41.0 | | | 85.0 | | |  |  |  |  |
|  |  |  | % within Gender | | | 100.0% | | | 100.0% | | | 100.0% | | |  |  |  |  |
| **Chi-Square Tests** | | | | | | | | | | | | | | | | | | |
|  | | | | | Value | | | df | | | Asymp. Sig. (2-sided) | | | Exact Sig. (2-sided) | | | Exact Sig. (1-sided) | |
| Pearson Chi-Square | | | | | 3.185^a^ | | | 1 | | | .074 | | |  | | |  | |
| Continuity Correction^b^ | | | | | 1.852 | | | 1 | | | .174 | | |  | | |  | |
| Likelihood Ratio | | | | | 3.426 | | | 1 | | | .064 | | |  | | |  | |
| Fisher's Exact Test | | | | |  | | |  | | |  | | | .102 | | | .086 | |
| Linear-by-Linear Association | | | | | 3.148 | | | 1 | | | .076 | | |  | | |  | |
| N of Valid Cases | | | | | 85 | | |  | | |  | | |  | | |  | |
| a. 2 cells (50.0%) have expected count less than 5. The minimum expected count is 2.89. | | | | | | | | | | | | | | | | | | |
| b. Computed only for a 2x2 table | | | | | | | | | | | | | | | | | | |

| **Symmetric Measures** | | | |
| --- | --- | --- | --- |
|  | | Value | Approx. Sig. |
| Nominal by Nominal | Phi | -.194 | .074 |
|  | Cramer's V | .194 | .074 |
| N of Valid Cases | | 85 |  |


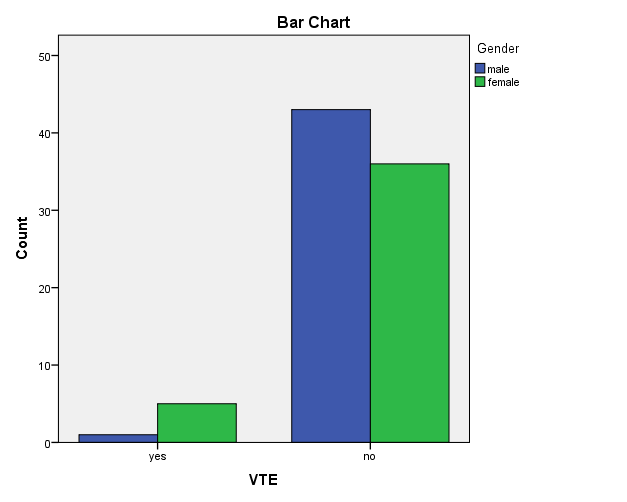


**Crosstabs**

| **Case Processing Summary** | | | | | | | | | | | | |  |
| --- | --- | --- | --- | --- | --- | --- | --- | --- | --- | --- | --- | --- | --- |
|  | | Cases | | | | | | | | | | |  |
|  |  | Valid | | | | Missing | | | | Total | | |  |
|  |  | N | | Percent | | N | | Percent | | N | Percent | |  |
| VTE * BMI | | 85 | | 100.0% | | 0 | | 0.0% | | 85 | 100.0% | |  |
| **VTE * BMI Crosstabulation** | | | | | | | | | | | | | |
|  | | | | | BMI | | | | | | | Total | |
|  |  |  |  |  | <30 | | >=30 | | not mentioned | | |  |  |
| VTE | yes | | Count | | 2 | | 3 | | 1 | | | 6 | |
|  |  |  | Expected Count | | 1.8 | | .6 | | 3.6 | | | 6.0 | |
|  |  |  | % within BMI | | 8.0% | | 33.3% | | 2.0% | | | 7.1% | |
|  | no | | Count | | 23 | | 6 | | 50 | | | 79 | |
|  |  |  | Expected Count | | 23.2 | | 8.4 | | 47.4 | | | 79.0 | |
|  |  |  | % within BMI | | 92.0% | | 66.7% | | 98.0% | | | 92.9% | |
| Total | | | Count | | 25 | | 9 | | 51 | | | 85 | |
|  |  |  | Expected Count | | 25.0 | | 9.0 | | 51.0 | | | 85.0 | |
|  |  |  | % within BMI | | 100.0% | | 100.0% | | 100.0% | | | 100.0% | |

| **Chi-Square Tests** | | | | | | |
| --- | --- | --- | --- | --- | --- | --- |
|  | Value | df | Asymp. Sig. (2-sided) | Exact Sig. (2-sided) | Exact Sig. (1-sided) | Point Probability |
| Pearson Chi-Square | 11.525^a^ | 2 | .003 | .005 |  |  |
| Likelihood Ratio | 8.137 | 2 | .017 | .017 |  |  |
| Fisher's Exact Test | 8.527 |  |  | .007 |  |  |
| Linear-by-Linear Association | 1.780^b^ | 1 | .182 | .222 | .132 | .063 |
| N of Valid Cases | 85 |  |  |  |  |  |
| a. 3 cells (50.0%) have expected count less than 5. The minimum expected count is .64. | | | | | | |
| b. The standardized statistic is 1.334. | | | | | | |

| **Symmetric Measures** | | | | |
| --- | --- | --- | --- | --- |
|  | | Value | Approx. Sig. | Exact Sig. |
| Nominal by Nominal | Phi | .368 | .003 | .005 |
|  | Cramer's V | .368 | .003 | .005 |
| N of Valid Cases | | 85 |  |  |


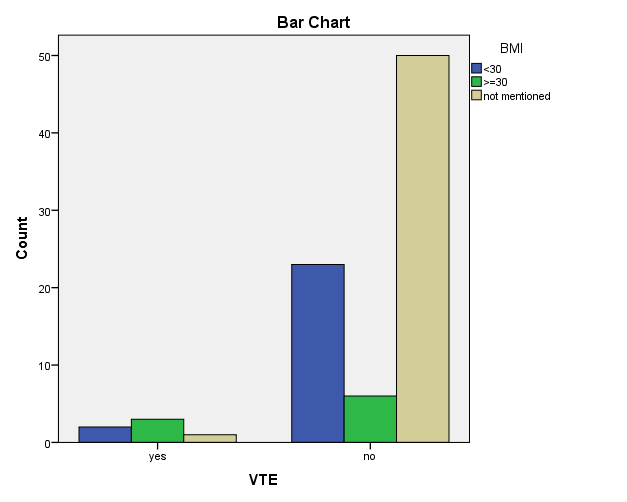


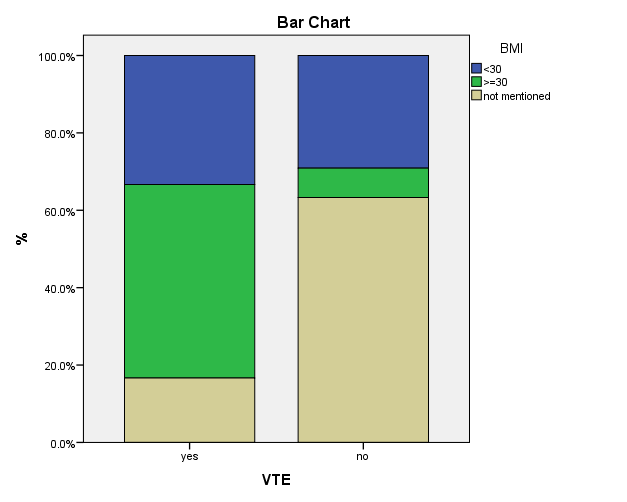


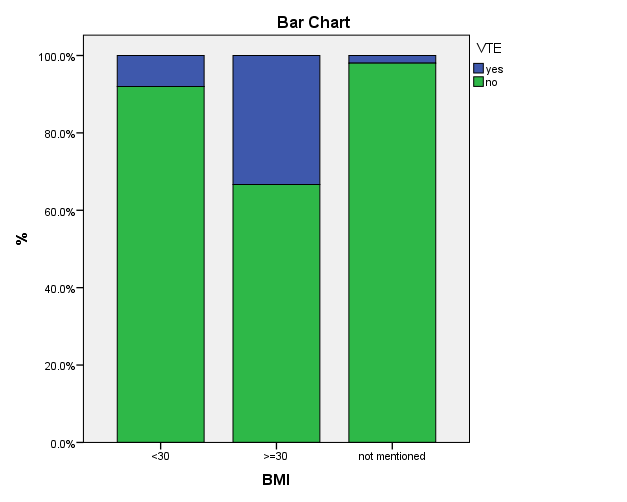


**Crosstabs**

| **Case Processing Summary** | | | | | | |
| --- | --- | --- | --- | --- | --- | --- |
|  | Cases | | | | | |
|  | Valid | | Missing | | Total | |
|  | N | Percent | N | Percent | N | Percent |
| VTE * OCP | 85 | 100.0% | 0 | 0.0% | 85 | 100.0% |

| **VTE * OCP Crosstabulation** | | | | | | |
| --- | --- | --- | --- | --- | --- | --- |
|  | | | OCP | | | Total |
|  |  |  | yes | no | N/A |  |
| VTE | yes | Count | 0 | 5 | 1 | 6 |
|  |  | Expected Count | .2 | 2.8 | 3.0 | 6.0 |
|  |  | % within OCP | 0.0% | 12.8% | 2.3% | 7.1% |
|  | no | Count | 3 | 34 | 42 | 79 |
|  |  | Expected Count | 2.8 | 36.2 | 40.0 | 79.0 |
|  |  | % within OCP | 100.0% | 87.2% | 97.7% | 92.9% |
| Total | | Count | 3 | 39 | 43 | 85 |
|  |  | Expected Count | 3.0 | 39.0 | 43.0 | 85.0 |
|  |  | % within OCP | 100.0% | 100.0% | 100.0% | 100.0% |

| **Chi-Square Tests** | | | | | | |
| --- | --- | --- | --- | --- | --- | --- |
|  | Value | df | Asymp. Sig. (2-sided) | Exact Sig. (2-sided) | Exact Sig. (1-sided) | Point Probability |
| Pearson Chi-Square | 3.670^a^ | 2 | .160 | .158 |  |  |
| Likelihood Ratio | 4.007 | 2 | .135 | .125 |  |  |
| Fisher's Exact Test | 3.435 |  |  | .215 |  |  |
| Linear-by-Linear Association | 1.843^b^ | 1 | .175 | .260 | .160 | .117 |
| N of Valid Cases | 85 |  |  |  |  |  |
| a. 4 cells (66.7%) have expected count less than 5. The minimum expected count is .21. | | | | | | |
| b. The standardized statistic is 1.358. | | | | | | |

| **Symmetric Measures** | | | | |
| --- | --- | --- | --- | --- |
|  | | Value | Approx. Sig. | Exact Sig. |
| Nominal by Nominal | Phi | .208 | .160 | .158 |
|  | Cramer's V | .208 | .160 | .158 |
| N of Valid Cases | | 85 |  |  |


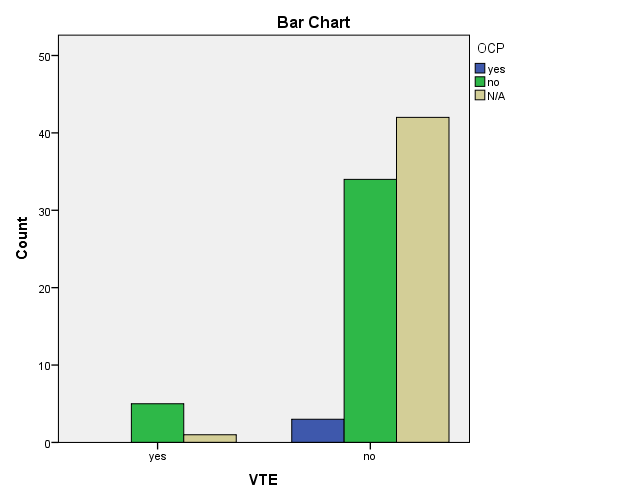


**Crosstabs**

| **Case Processing Summary** | | | | | | | | | | | | | | | | | | | | | | |  |  |
| --- | --- | --- | --- | --- | --- | --- | --- | --- | --- | --- | --- | --- | --- | --- | --- | --- | --- | --- | --- | --- | --- | --- | --- | --- |
|  | | | Cases | | | | | | | | | | | | | | | | | | | |  |  |
|  |  |  | Valid | | | | | | | Missing | | | | | | | | Total | | | | |  |  |
|  |  |  | N | | | Percent | | | | N | | | | Percent | | | | N | | | Percent | |  |  |
| VTE * Active Cancer | | | 85 | | | 100.0% | | | | 0 | | | | 0.0% | | | | 85 | | | 100.0% | |  |  |
| **VTE * Active Cancer Crosstabulation** | | | | | | | | | | | | | | | | | | | |  |  |  |  |  |
|  | | | | | | | | | Active Cancer | | | | | | | Total | | | |  |  |  |  |  |
|  |  |  |  |  |  |  |  |  | yes | | | | no | | |  |  |  |  |  |  |  |  |  |
| VTE | yes | Count | | | | | | | 2 | | | | 4 | | | 6 | | | |  |  |  |  |  |
|  |  | Expected Count | | | | | | | 1.8 | | | | 4.2 | | | 6.0 | | | |  |  |  |  |  |
|  |  | % within Active Cancer | | | | | | | 7.7% | | | | 6.8% | | | 7.1% | | | |  |  |  |  |  |
|  | no | Count | | | | | | | 24 | | | | 55 | | | 79 | | | |  |  |  |  |  |
|  |  | Expected Count | | | | | | | 24.2 | | | | 54.8 | | | 79.0 | | | |  |  |  |  |  |
|  |  | % within Active Cancer | | | | | | | 92.3% | | | | 93.2% | | | 92.9% | | | |  |  |  |  |  |
| Total | | Count | | | | | | | 26 | | | | 59 | | | 85 | | | |  |  |  |  |  |
|  |  | Expected Count | | | | | | | 26.0 | | | | 59.0 | | | 85.0 | | | |  |  |  |  |  |
|  |  | % within Active Cancer | | | | | | | 100.0% | | | | 100.0% | | | 100.0% | | | |  |  |  |  |  |
| **Chi-Square Tests** | | | | | | | | | | | | | | | | | | | | | | | | |
|  | | | | | Value | | | df | | | | Asymp. Sig. (2-sided) | | | | | Exact Sig. (2-sided) | | | | | Exact Sig. (1-sided) | | Point Probability |
| Pearson Chi-Square | | | | | .023^a^ | | | 1 | | | | .880 | | | | | 1.000 | | | | | .599 | |  |
| Continuity Correction^b^ | | | | | .000 | | | 1 | | | | 1.000 | | | | |  | | | | |  | |  |
| Likelihood Ratio | | | | | .023 | | | 1 | | | | .881 | | | | | 1.000 | | | | | .599 | |  |
| Fisher's Exact Test | | | | |  | | |  | | | |  | | | | | 1.000 | | | | | .599 | |  |
| Linear-by-Linear Association | | | | | .023^c^ | | | 1 | | | | .880 | | | | | 1.000 | | | | | .599 | | .338 |
| N of Valid Cases | | | | | 85 | | |  | | | |  | | | | |  | | | | |  | |  |
| a. 2 cells (50.0%) have expected count less than 5. The minimum expected count is 1.84. | | | | | | | | | | | | | | | | | | | | | | | | |
| b. Computed only for a 2x2 table | | | | | | | | | | | | | | | | | | | | | | | | |
| c. The standardized statistic is .150. | | | | | | | | | | | | | | | | | | | | | | | | |
| **Symmetric Measures** | | | | | | | | | | | | | | | | | | |  |  |  |  |  |  |
|  | | | | | | | Value | | | | Approx. Sig. | | | | Exact Sig. | | | |  |  |  |  |  |  |
| Nominal by Nominal | | | | Phi | | | .016 | | | | .880 | | | | 1.000 | | | |  |  |  |  |  |  |
|  |  |  |  | Cramer's V | | | .016 | | | | .880 | | | | 1.000 | | | |  |  |  |  |  |  |
| N of Valid Cases | | | | | | | 85 | | | |  | | | |  | | | |  |  |  |  |  |  |


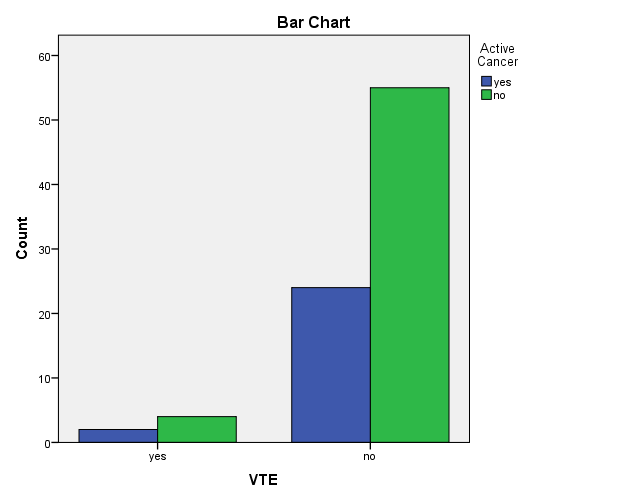


**Crosstabs**

| **Case Processing Summary** | | | | | | | | | | | | |
| --- | --- | --- | --- | --- | --- | --- | --- | --- | --- | --- | --- | --- |
|  | | | Cases | | | | | | | | | |
|  |  |  | Valid | | | Missing | | | Total | | | |
|  |  |  | N | Percent | | N | | Percent | N | | Percent | |
| VTE * Chronic Liver Disease (CLD) | | | 85 | 100.0% | | 0 | | 0.0% | 85 | | 100.0% | |
| **VTE * Chronic Liver Disease (CLD) Crosstabulation** | | | | | | | | | | | |  |
|  | | | | | Chronic Liver Disease (CLD) | | | | | Total | |  |
|  |  |  |  |  | yes | | no | | |  |  |  |
| VTE | yes | Count | | | 0 | | 6 | | | 6 | |  |
|  |  | Expected Count | | | .1 | | 5.9 | | | 6.0 | |  |
|  |  | % within Chronic Liver Disease (CLD) | | | 0.0% | | 7.2% | | | 7.1% | |  |
|  | no | Count | | | 2 | | 77 | | | 79 | |  |
|  |  | Expected Count | | | 1.9 | | 77.1 | | | 79.0 | |  |
|  |  | % within Chronic Liver Disease (CLD) | | | 100.0% | | 92.8% | | | 92.9% | |  |
| Total | | Count | | | 2 | | 83 | | | 85 | |  |
|  |  | Expected Count | | | 2.0 | | 83.0 | | | 85.0 | |  |
|  |  | % within Chronic Liver Disease (CLD) | | | 100.0% | | 100.0% | | | 100.0% | |  |

| **Chi-Square Tests** | | | | | | |
| --- | --- | --- | --- | --- | --- | --- |
|  | Value | df | Asymp. Sig. (2-sided) | Exact Sig. (2-sided) | Exact Sig. (1-sided) | Point Probability |
| Pearson Chi-Square | .156^a^ | 1 | .693 | 1.000 | .863 |  |
| Continuity Correction^b^ | .000 | 1 | 1.000 |  |  |  |
| Likelihood Ratio | .296 | 1 | .586 | 1.000 | .863 |  |
| Fisher's Exact Test |  |  |  | 1.000 | .863 |  |
| Linear-by-Linear Association | .154^c^ | 1 | .695 | 1.000 | .863 | .863 |
| N of Valid Cases | 85 |  |  |  |  |  |
| a. 2 cells (50.0%) have expected count less than 5. The minimum expected count is .14. | | | | | | |
| b. Computed only for a 2x2 table | | | | | | |
| c. The standardized statistic is -.392. | | | | | | |

| **Symmetric Measures** | | | | |
| --- | --- | --- | --- | --- |
|  | | Value | Approx. Sig. | Exact Sig. |
| Nominal by Nominal | Phi | -.043 | .693 | 1.000 |
|  | Cramer's V | .043 | .693 | 1.000 |
| N of Valid Cases | | 85 |  |  |


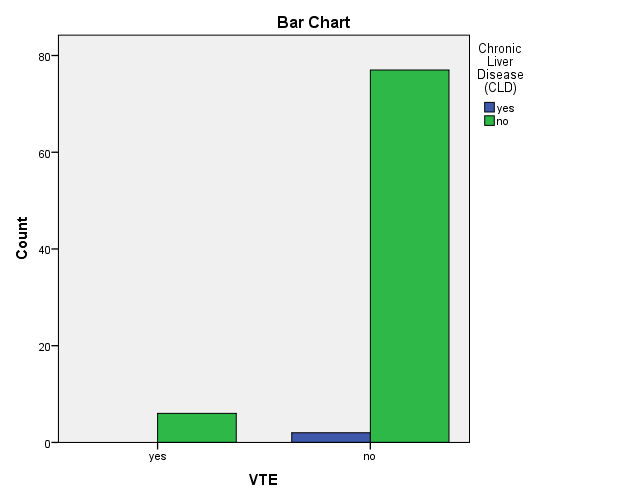


**Crosstabs**

| **Case Processing Summary** | | | | | | |
| --- | --- | --- | --- | --- | --- | --- |
|  | Cases | | | | | |
|  | Valid | | Missing | | Total | |
|  | N | Percent | N | Percent | N | Percent |
| VTE * Pre-operative platelets | 85 | 100.0% | 0 | 0.0% | 85 | 100.0% |

| **VTE * Pre-operative platelets Crosstabulation** | | | | | | |
| --- | --- | --- | --- | --- | --- | --- |
|  | | | Pre-operative platelets | | | Total |
|  |  |  | 50-99 | 100-450 | >450 |  |
| VTE | yes | Count | 1 | 4 | 1 | 6 |
|  |  | Expected Count | .8 | 4.8 | .4 | 6.0 |
|  |  | % within Pre-operative platelets | 9.1% | 5.9% | 16.7% | 7.1% |
|  | no | Count | 10 | 64 | 5 | 79 |
|  |  | Expected Count | 10.2 | 63.2 | 5.6 | 79.0 |
|  |  | % within Pre-operative platelets | 90.9% | 94.1% | 83.3% | 92.9% |
| Total | | Count | 11 | 68 | 6 | 85 |
|  |  | Expected Count | 11.0 | 68.0 | 6.0 | 85.0 |
|  |  | % within Pre-operative platelets | 100.0% | 100.0% | 100.0% | 100.0% |

| **Chi-Square Tests** | | | | | | |
| --- | --- | --- | --- | --- | --- | --- |
|  | Value | df | Asymp. Sig. (2-sided) | Exact Sig. (2-sided) | Exact Sig. (1-sided) | Point Probability |
| Pearson Chi-Square | 1.057^a^ | 2 | .590 | .738 |  |  |
| Likelihood Ratio | .842 | 2 | .656 | 1.000 |  |  |
| Fisher's Exact Test | 1.999 |  |  | .345 |  |  |
| Linear-by-Linear Association | .112^b^ | 1 | .738 | 1.000 | .571 | .377 |
| N of Valid Cases | 85 |  |  |  |  |  |
| a. 3 cells (50.0%) have expected count less than 5. The minimum expected count is .42. | | | | | | |
| b. The standardized statistic is -.335. | | | | | | |

| **Symmetric Measures** | | | | |
| --- | --- | --- | --- | --- |
|  | | Value | Approx. Sig. | Exact Sig. |
| Nominal by Nominal | Phi | .112 | .590 | .738 |
|  | Cramer's V | .112 | .590 | .738 |
| N of Valid Cases | | 85 |  |  |


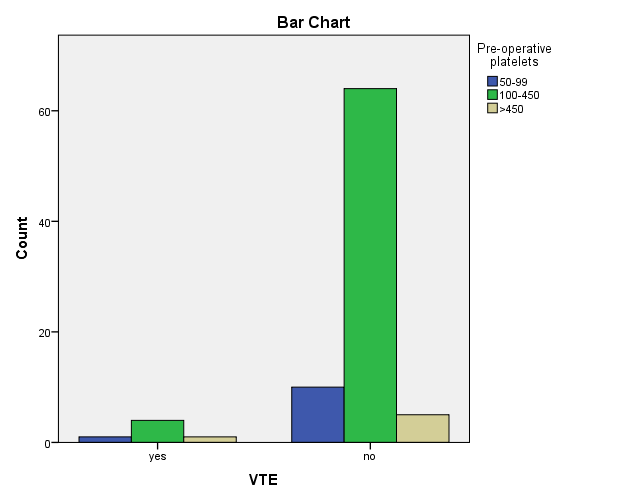


**Crosstabs**

| **Case Processing Summary** | | | | | | | | | | | | | | | | | |  |  |
| --- | --- | --- | --- | --- | --- | --- | --- | --- | --- | --- | --- | --- | --- | --- | --- | --- | --- | --- | --- |
|  | | | Cases | | | | | | | | | | | | | | |  |  |
|  |  |  | Valid | | | | | Missing | | | | | Total | | | | |  |  |
|  |  |  | N | | Percent | | | N | | | Percent | | N | | | Percent | |  |  |
| VTE * Antiplatelets | | | 85 | | 100.0% | | | 0 | | | 0.0% | | 85 | | | 100.0% | |  |  |
| **VTE * Antiplatelets Crosstabulation** | | | | | | | | | | | | | | |  |  |  |  |  |
|  | | | | | | | Antiplatelets | | | | | Total | | |  |  |  |  |  |
|  |  |  |  |  |  |  | yes | | | no | |  |  |  |  |  |  |  |  |
| VTE | yes | Count | | | | | 1 | | | 5 | | 6 | | |  |  |  |  |  |
|  |  | Expected Count | | | | | .8 | | | 5.2 | | 6.0 | | |  |  |  |  |  |
|  |  | % within Antiplatelets | | | | | 9.1% | | | 6.8% | | 7.1% | | |  |  |  |  |  |
|  | no | Count | | | | | 10 | | | 69 | | 79 | | |  |  |  |  |  |
|  |  | Expected Count | | | | | 10.2 | | | 68.8 | | 79.0 | | |  |  |  |  |  |
|  |  | % within Antiplatelets | | | | | 90.9% | | | 93.2% | | 92.9% | | |  |  |  |  |  |
| Total | | Count | | | | | 11 | | | 74 | | 85 | | |  |  |  |  |  |
|  |  | Expected Count | | | | | 11.0 | | | 74.0 | | 85.0 | | |  |  |  |  |  |
|  |  | % within Antiplatelets | | | | | 100.0% | | | 100.0% | | 100.0% | | |  |  |  |  |  |
| **Chi-Square Tests** | | | | | | | | | | | | | | | | | | | |
|  | | | | Value | | df | | | Asymp. Sig. (2-sided) | | | | | Exact Sig. (2-sided) | | | Exact Sig. (1-sided) | | Point Probability |
| Pearson Chi-Square | | | | .080^a^ | | 1 | | | .778 | | | | | 1.000 | | | .576 | |  |
| Continuity Correction^b^ | | | | .000 | | 1 | | | 1.000 | | | | |  | | |  | |  |
| Likelihood Ratio | | | | .074 | | 1 | | | .785 | | | | | 1.000 | | | .576 | |  |
| Fisher's Exact Test | | | |  | |  | | |  | | | | | .576 | | | .576 | |  |
| Linear-by-Linear Association | | | | .079^c^ | | 1 | | | .779 | | | | | 1.000 | | | .576 | | .405 |
| N of Valid Cases | | | | 85 | |  | | |  | | | | |  | | |  | |  |
| a. 1 cells (25.0%) have expected count less than 5. The minimum expected count is .78. | | | | | | | | | | | | | | | | | | | |
| b. Computed only for a 2x2 table | | | | | | | | | | | | | | | | | | | |
| c. The standardized statistic is .280. | | | | | | | | | | | | | | | | | | | |

| **Symmetric Measures** | | | | |
| --- | --- | --- | --- | --- |
|  | | Value | Approx. Sig. | Exact Sig. |
| Nominal by Nominal | Phi | .031 | .778 | 1.000 |
|  | Cramer's V | .031 | .778 | 1.000 |
| N of Valid Cases | | 85 |  |  |


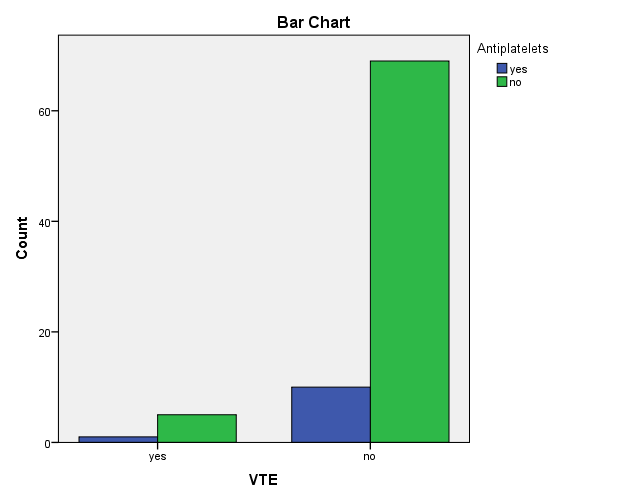


**Crosstabs**

| **Case Processing Summary** | | | | | | |
| --- | --- | --- | --- | --- | --- | --- |
|  | Cases | | | | | |
|  | Valid | | Missing | | Total | |
|  | N | Percent | N | Percent | N | Percent |
| VTE * Anticoagulation | 85 | 100.0% | 0 | 0.0% | 85 | 100.0% |

| **VTE * Anticoagulation Crosstabulation** | | | | | |
| --- | --- | --- | --- | --- | --- |
|  | | | Anticoagulation | | Total |
|  |  |  | yes | no |  |
| VTE | yes | Count | 1 | 5 | 6 |
|  |  | Expected Count | 1.1 | 4.9 | 6.0 |
|  |  | % within Anticoagulation | 6.7% | 7.1% | 7.1% |
|  | no | Count | 14 | 65 | 79 |
|  |  | Expected Count | 13.9 | 65.1 | 79.0 |
|  |  | % within Anticoagulation | 93.3% | 92.9% | 92.9% |
| Total | | Count | 15 | 70 | 85 |
|  |  | Expected Count | 15.0 | 70.0 | 85.0 |
|  |  | % within Anticoagulation | 100.0% | 100.0% | 100.0% |

| **Chi-Square Tests** | | | | | | |
| --- | --- | --- | --- | --- | --- | --- |
|  | Value | df | Asymp. Sig. (2-sided) | Exact Sig. (2-sided) | Exact Sig. (1-sided) | Point Probability |
| Pearson Chi-Square | .004^a^ | 1 | .948 | 1.000 | .715 |  |
| Continuity Correction^b^ | .000 | 1 | 1.000 |  |  |  |
| Likelihood Ratio | .004 | 1 | .948 | 1.000 | .715 |  |
| Fisher's Exact Test |  |  |  | 1.000 | .715 |  |
| Linear-by-Linear Association | .004^c^ | 1 | .948 | 1.000 | .715 | .415 |
| N of Valid Cases | 85 |  |  |  |  |  |
| a. 2 cells (50.0%) have expected count less than 5. The minimum expected count is 1.06. | | | | | | |
| b. Computed only for a 2x2 table | | | | | | |
| c. The standardized statistic is -.065. | | | | | | |

| **Symmetric Measures** | | | | |
| --- | --- | --- | --- | --- |
|  | | Value | Approx. Sig. | Exact Sig. |
| Nominal by Nominal | Phi | -.007 | .948 | 1.000 |
|  | Cramer's V | .007 | .948 | 1.000 |
| N of Valid Cases | | 85 |  |  |


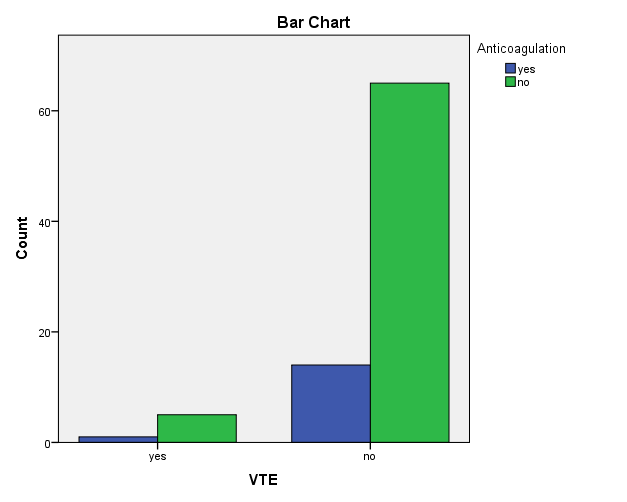


**Crosstabs**

| **Case Processing Summary** | | | | | | |
| --- | --- | --- | --- | --- | --- | --- |
|  | Cases | | | | | |
|  | Valid | | Missing | | Total | |
|  | N | Percent | N | Percent | N | Percent |
| VTE * Vaccination | 85 | 100.0% | 0 | 0.0% | 85 | 100.0% |

| **VTE * Vaccination Crosstabulation** | | | | | | |
| --- | --- | --- | --- | --- | --- | --- |
|  | | | Vaccination | | | Total |
|  |  |  | pre-operative | post-operative | not mentioned |  |
| VTE | yes | Count | 4 | 2 | 0 | 6 |
|  |  | Expected Count | 2.3 | 3.1 | .6 | 6.0 |
|  |  | % within Vaccination | 12.5% | 4.5% | 0.0% | 7.1% |
|  | no | Count | 28 | 42 | 9 | 79 |
|  |  | Expected Count | 29.7 | 40.9 | 8.4 | 79.0 |
|  |  | % within Vaccination | 87.5% | 95.5% | 100.0% | 92.9% |
| Total | | Count | 32 | 44 | 9 | 85 |
|  |  | Expected Count | 32.0 | 44.0 | 9.0 | 85.0 |
|  |  | % within Vaccination | 100.0% | 100.0% | 100.0% | 100.0% |

| **Chi-Square Tests** | | | | | | |
| --- | --- | --- | --- | --- | --- | --- |
|  | Value | df | Asymp. Sig. (2-sided) | Exact Sig. (2-sided) | Exact Sig. (1-sided) | Point Probability |
| Pearson Chi-Square | 2.551^a^ | 2 | .279 | .375 |  |  |
| Likelihood Ratio | 2.992 | 2 | .224 | .336 |  |  |
| Fisher's Exact Test | 1.876 |  |  | .295 |  |  |
| Linear-by-Linear Association | 2.446^b^ | 1 | .118 | .186 | .104 | .082 |
| N of Valid Cases | 85 |  |  |  |  |  |
| a. 3 cells (50.0%) have expected count less than 5. The minimum expected count is .64. | | | | | | |
| b. The standardized statistic is 1.564. | | | | | | |

| **Symmetric Measures** | | | | |
| --- | --- | --- | --- | --- |
|  | | Value | Approx. Sig. | Exact Sig. |
| Nominal by Nominal | Phi | .173 | .279 | .375 |
|  | Cramer's V | .173 | .279 | .375 |
| N of Valid Cases | | 85 |  |  |


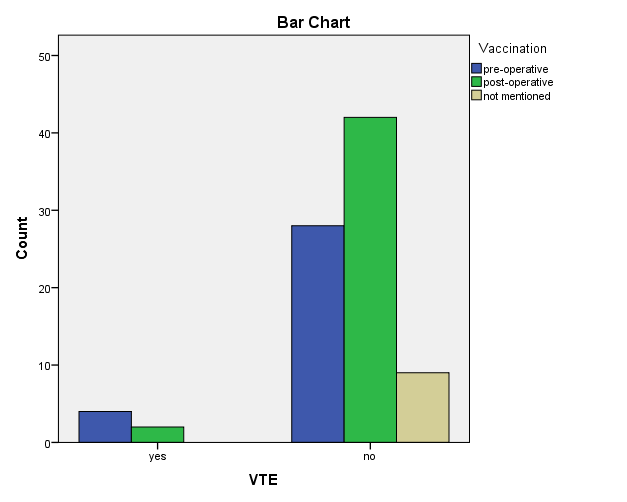


**Crosstabs**

| **Case Processing Summary** | | | | | | | | | | | | | | | | | |  |  |  |  |
| --- | --- | --- | --- | --- | --- | --- | --- | --- | --- | --- | --- | --- | --- | --- | --- | --- | --- | --- | --- | --- | --- |
|  | | | Cases | | | | | | | | | | | | | | |  |  |  |  |
|  |  |  | Valid | | | | | Missing | | | | Total | | | | | |  |  |  |  |
|  |  |  | N | | Percent | | | N | | Percent | | N | | | Percent | | |  |  |  |  |
| VTE * Referring Dr | | | 85 | | 100.0% | | | 0 | | 0.0% | | 85 | | | 100.0% | | |  |  |  |  |
| **VTE * Referring Dr Crosstabulation** | | | | | | | | | | | | | | | | | | | | |  |
|  | | | | | | | Referring Dr | | | | | | | | | | | | Total | |  |
|  |  |  |  |  |  |  | Haematology | | | | Oncology | | | Upper GI | | others | | |  |  |  |
| VTE | yes | Count | | | | | 4 | | | | 1 | | | 0 | | 1 | | | 6 | |  |
|  |  | Expected Count | | | | | 2.8 | | | | .7 | | | 2.1 | | .4 | | | 6.0 | |  |
|  |  | % within Referring Dr | | | | | 10.3% | | | | 10.0% | | | 0.0% | | 16.7% | | | 7.1% | |  |
|  | no | Count | | | | | 35 | | | | 9 | | | 30 | | 5 | | | 79 | |  |
|  |  | Expected Count | | | | | 36.2 | | | | 9.3 | | | 27.9 | | 5.6 | | | 79.0 | |  |
|  |  | % within Referring Dr | | | | | 89.7% | | | | 90.0% | | | 100.0% | | 83.3% | | | 92.9% | |  |
| Total | | Count | | | | | 39 | | | | 10 | | | 30 | | 6 | | | 85 | |  |
|  |  | Expected Count | | | | | 39.0 | | | | 10.0 | | | 30.0 | | 6.0 | | | 85.0 | |  |
|  |  | % within Referring Dr | | | | | 100.0% | | | | 100.0% | | | 100.0% | | 100.0% | | | 100.0% | |  |
| **Chi-Square Tests** | | | | | | | | | | | | | | | | | | | | | |
|  | | | | Value | | df | | | Asymp. Sig. (2-sided) | | | | Exact Sig. (2-sided) | | | | Exact Sig. (1-sided) | | | Point Probability | |
| Pearson Chi-Square | | | | 3.862^a^ | | 3 | | | .277 | | | | .212 | | | |  | | |  | |
| Likelihood Ratio | | | | 5.675 | | 3 | | | .129 | | | | .133 | | | |  | | |  | |
| Fisher's Exact Test | | | | 4.911 | |  | | |  | | | | .142 | | | |  | | |  | |
| Linear-by-Linear Association | | | | .793^b^ | | 1 | | | .373 | | | | .439 | | | | .257 | | | .122 | |
| N of Valid Cases | | | | 85 | |  | | |  | | | |  | | | |  | | |  | |
| a. 4 cells (50.0%) have expected count less than 5. The minimum expected count is .42. | | | | | | | | | | | | | | | | | | | | | |
| b. The standardized statistic is .891. | | | | | | | | | | | | | | | | | | | | | |

| **Symmetric Measures** | | | | |
| --- | --- | --- | --- | --- |
|  | | Value | Approx. Sig. | Exact Sig. |
| Nominal by Nominal | Phi | .213 | .277 | .212 |
|  | Cramer's V | .213 | .277 | .212 |
| N of Valid Cases | | 85 |  |  |


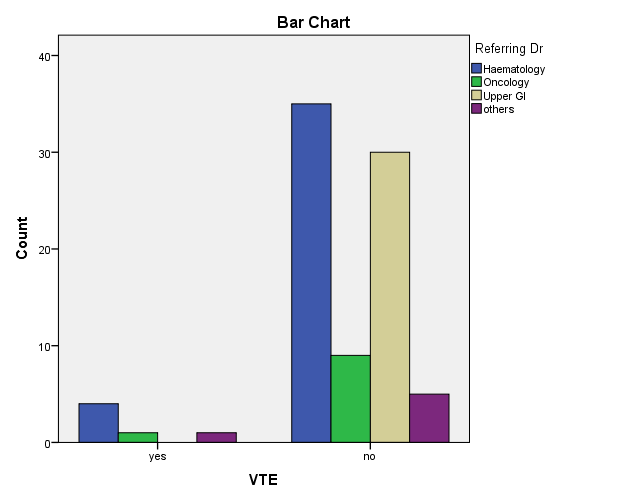


**Crosstabs**

| **Case Processing Summary** | | | | | | |
| --- | --- | --- | --- | --- | --- | --- |
|  | Cases | | | | | |
|  | Valid | | Missing | | Total | |
|  | N | Percent | N | Percent | N | Percent |
| VTE * Indication | 85 | 100.0% | 0 | 0.0% | 85 | 100.0% |

| **VTE * Indication Crosstabulation** | | | | | | | | | | |
| --- | --- | --- | --- | --- | --- | --- | --- | --- | --- | --- |
|  | | | Indication | | | | | | | Total |
|  |  |  | Benign haematology | Malignant haematology | Solid tumors | Spontaneous rupture | Traumatic rupture | Iatrogenic trauma | Others |  |
| VTE | yes | Count | 3 | 1 | 0 | 0 | 0 | 1 | 1 | 6 |
|  |  | Expected Count | 2.0 | .8 | .6 | .3 | 1.0 | .6 | .6 | 6.0 |
|  |  | % within Indication | 10.3% | 9.1% | 0.0% | 0.0% | 0.0% | 11.1% | 11.1% | 7.1% |
|  | no | Count | 26 | 10 | 9 | 4 | 14 | 8 | 8 | 79 |
|  |  | Expected Count | 27.0 | 10.2 | 8.4 | 3.7 | 13.0 | 8.4 | 8.4 | 79.0 |
|  |  | % within Indication | 89.7% | 90.9% | 100.0% | 100.0% | 100.0% | 88.9% | 88.9% | 92.9% |
| Total | | Count | 29 | 11 | 9 | 4 | 14 | 9 | 9 | 85 |
|  |  | Expected Count | 29.0 | 11.0 | 9.0 | 4.0 | 14.0 | 9.0 | 9.0 | 85.0 |
|  |  | % within Indication | 100.0% | 100.0% | 100.0% | 100.0% | 100.0% | 100.0% | 100.0% | 100.0% |

| **Chi-Square Tests** | | | | | | |
| --- | --- | --- | --- | --- | --- | --- |
|  | Value | df | Asymp. Sig. (2-sided) | Exact Sig. (2-sided) | Exact Sig. (1-sided) | Point Probability |
| Pearson Chi-Square | 3.048^a^ | 6 | .803 | .878 |  |  |
| Likelihood Ratio | 4.826 | 6 | .566 | .756 |  |  |
| Fisher's Exact Test | 3.178 |  |  | .851 |  |  |
| Linear-by-Linear Association | .126^b^ | 1 | .723 | .780 | .410 | .073 |
| N of Valid Cases | 85 |  |  |  |  |  |
| a. 8 cells (57.1%) have expected count less than 5. The minimum expected count is .28. | | | | | | |
| b. The standardized statistic is .354. | | | | | | |

| **Symmetric Measures** | | | | |
| --- | --- | --- | --- | --- |
|  | | Value | Approx. Sig. | Exact Sig. |
| Nominal by Nominal | Phi | .189 | .803 | .878 |
|  | Cramer's V | .189 | .803 | .878 |
| N of Valid Cases | | 85 |  |  |


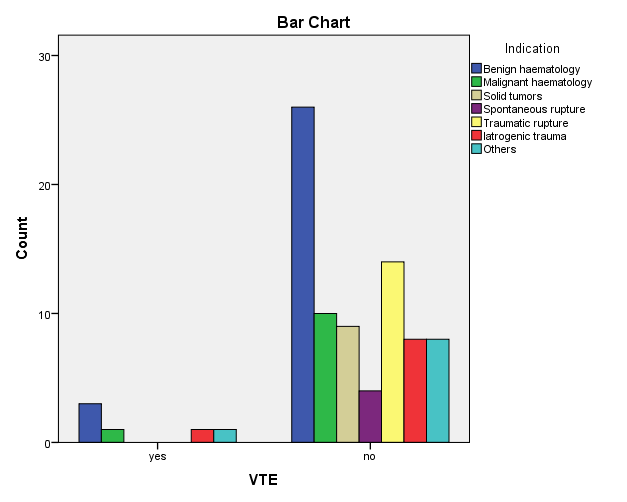


**Crosstabs**

| **Case Processing Summary** | | | | | | |
| --- | --- | --- | --- | --- | --- | --- |
|  | Cases | | | | | |
|  | Valid | | Missing | | Total | |
|  | N | Percent | N | Percent | N | Percent |
| VTE * Date of Surgery | 85 | 100.0% | 0 | 0.0% | 85 | 100.0% |

| **VTE * Date of Surgery Crosstabulation** | | | | | | | | | | | | | | |
| --- | --- | --- | --- | --- | --- | --- | --- | --- | --- | --- | --- | --- | --- | --- |
|  | | | Date of Surgery | | | | | | | | | | | Total |
|  |  |  | 2007 | 2008 | 2009 | 2010 | 2011 | 2012 | 2013 | 2014 | 2015 | 2016 | 2017 |  |
| VTE | yes | Count | 0 | 0 | 1 | 1 | 1 | 0 | 0 | 1 | 0 | 1 | 1 | 6 |
|  |  | Expected Count | .5 | 1.0 | .4 | .8 | .6 | .6 | .4 | .6 | .5 | .4 | .4 | 6.0 |
|  |  | % within Date of Surgery | 0.0% | 0.0% | 20.0% | 9.1% | 12.5% | 0.0% | 0.0% | 12.5% | 0.0% | 20.0% | 16.7% | 7.1% |
|  | no | Count | 7 | 14 | 4 | 10 | 7 | 8 | 6 | 7 | 7 | 4 | 5 | 79 |
|  |  | Expected Count | 6.5 | 13.0 | 4.6 | 10.2 | 7.4 | 7.4 | 5.6 | 7.4 | 6.5 | 4.6 | 5.6 | 79.0 |
|  |  | % within Date of Surgery | 100.0% | 100.0% | 80.0% | 90.9% | 87.5% | 100.0% | 100.0% | 87.5% | 100.0% | 80.0% | 83.3% | 92.9% |
| Total | | Count | 7 | 14 | 5 | 11 | 8 | 8 | 6 | 8 | 7 | 5 | 6 | 85 |
|  |  | Expected Count | 7.0 | 14.0 | 5.0 | 11.0 | 8.0 | 8.0 | 6.0 | 8.0 | 7.0 | 5.0 | 6.0 | 85.0 |
|  |  | % within Date of Surgery | 100.0% | 100.0% | 100.0% | 100.0% | 100.0% | 100.0% | 100.0% | 100.0% | 100.0% | 100.0% | 100.0% | 100.0% |

| **Chi-Square Tests** | | | | | | |
| --- | --- | --- | --- | --- | --- | --- |
|  | Value | df | Asymp. Sig. (2-sided) | Exact Sig. (2-sided) | Exact Sig. (1-sided) | Point Probability |
| Pearson Chi-Square | 7.378^a^ | 10 | .689 | .701 |  |  |
| Likelihood Ratio | 9.203 | 10 | .513 | .698 |  |  |
| Fisher's Exact Test | 7.993 |  |  | .470 |  |  |
| Linear-by-Linear Association | 1.208^b^ | 1 | .272 | .281 | .155 | .030 |
| N of Valid Cases | 85 |  |  |  |  |  |
| a. 13 cells (59.1%) have expected count less than 5. The minimum expected count is .35. | | | | | | |
| b. The standardized statistic is -1.099. | | | | | | |

| **Symmetric Measures** | | | | |
| --- | --- | --- | --- | --- |
|  | | Value | Approx. Sig. | Exact Sig. |
| Nominal by Nominal | Phi | .295 | .689 | .701 |
|  | Cramer's V | .295 | .689 | .701 |
| N of Valid Cases | | 85 |  |  |


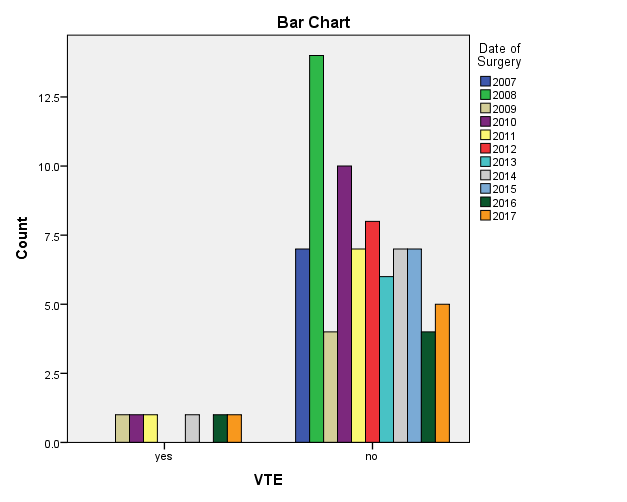


**Crosstabs**

| **Case Processing Summary** | | | | | | | | | | | | | | | | |  |  |  |
| --- | --- | --- | --- | --- | --- | --- | --- | --- | --- | --- | --- | --- | --- | --- | --- | --- | --- | --- | --- |
|  | | Cases | | | | | | | | | | | | | | |  |  |  |
|  |  | Valid | | | | | Missing | | | | | Total | | | | |  |  |  |
|  |  | N | | Percent | | | N | | Percent | | | N | | | Percent | |  |  |  |
| VTE * Type | | 85 | | 100.0% | | | 0 | | 0.0% | | | 85 | | | 100.0% | |  |  |  |
| **VTE * Type Crosstabulation** | | | | | | | | | | | | | | | |  |  |  |  |
|  | | | | | | Type | | | | | | | Total | | |  |  |  |  |
|  |  |  |  |  |  | Emergency | | | | Elective | | |  |  |  |  |  |  |  |
| VTE | yes | | Count | | | 0 | | | | 6 | | | 6 | | |  |  |  |  |
|  |  |  | Expected Count | | | 2.1 | | | | 3.9 | | | 6.0 | | |  |  |  |  |
|  |  |  | % within Type | | | 0.0% | | | | 10.9% | | | 7.1% | | |  |  |  |  |
|  | no | | Count | | | 30 | | | | 49 | | | 79 | | |  |  |  |  |
|  |  |  | Expected Count | | | 27.9 | | | | 51.1 | | | 79.0 | | |  |  |  |  |
|  |  |  | % within Type | | | 100.0% | | | | 89.1% | | | 92.9% | | |  |  |  |  |
| Total | | | Count | | | 30 | | | | 55 | | | 85 | | |  |  |  |  |
|  |  |  | Expected Count | | | 30.0 | | | | 55.0 | | | 85.0 | | |  |  |  |  |
|  |  |  | % within Type | | | 100.0% | | | | 100.0% | | | 100.0% | | |  |  |  |  |
| **Chi-Square Tests** | | | | | | | | | | | | | | | | | | | |
|  | | | | | Value | | | df | | | Asymp. Sig. (2-sided) | | | Exact Sig. (2-sided) | | | | Exact Sig. (1-sided) | Point Probability |
| Pearson Chi-Square | | | | | 3.521^a^ | | | 1 | | | .061 | | | .086 | | | | .066 |  |
| Continuity Correction^b^ | | | | | 2.055 | | | 1 | | | .152 | | |  | | | |  |  |
| Likelihood Ratio | | | | | 5.470 | | | 1 | | | .019 | | | .086 | | | | .066 |  |
| Fisher's Exact Test | | | | |  | | |  | | |  | | | .086 | | | | .066 |  |
| Linear-by-Linear Association | | | | | 3.480^c^ | | | 1 | | | .062 | | | .086 | | | | .066 | .066 |
| N of Valid Cases | | | | | 85 | | |  | | |  | | |  | | | |  |  |
| a. 2 cells (50.0%) have expected count less than 5. The minimum expected count is 2.12. | | | | | | | | | | | | | | | | | | | |
| b. Computed only for a 2x2 table | | | | | | | | | | | | | | | | | | | |
| c. The standardized statistic is -1.865. | | | | | | | | | | | | | | | | | | | |

| **Symmetric Measures** | | | | |
| --- | --- | --- | --- | --- |
|  | | Value | Approx. Sig. | Exact Sig. |
| Nominal by Nominal | Phi | -.204 | .061 | .086 |
|  | Cramer's V | .204 | .061 | .086 |
| N of Valid Cases | | 85 |  |  |


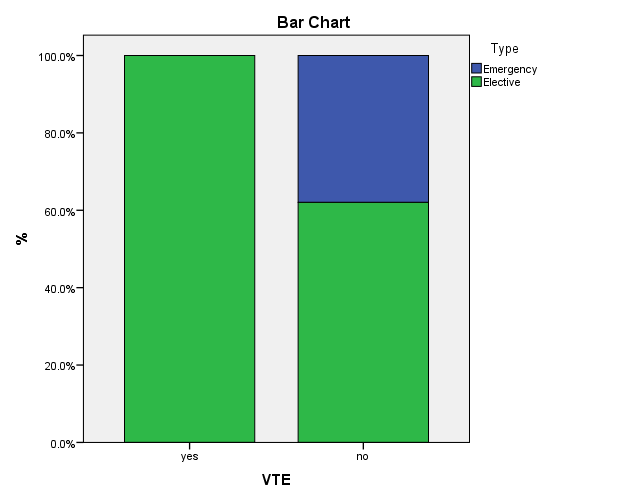


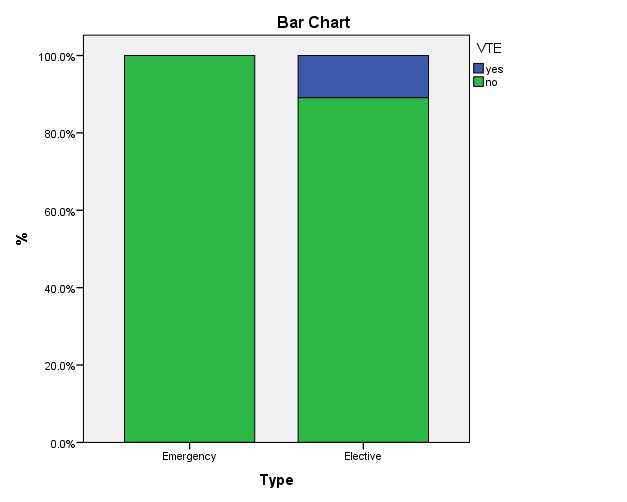


**Crosstabs**

| **Case Processing Summary** | | | | | | | | | | | | | | | | | |  |  |
| --- | --- | --- | --- | --- | --- | --- | --- | --- | --- | --- | --- | --- | --- | --- | --- | --- | --- | --- | --- |
|  | | | Cases | | | | | | | | | | | | | | |  |  |
|  |  |  | Valid | | | | | Missing | | | | Total | | | | | |  |  |
|  |  |  | N | | Percent | | | N | | | Percent | N | | | Percent | | |  |  |
| VTE * Procedure | | | 85 | | 100.0% | | | 0 | | | 0.0% | 85 | | | 100.0% | | |  |  |
| **VTE * Procedure Crosstabulation** | | | | | | | | | | | | | | | |  |  |  |  |
|  | | | | | | Procedure | | | | | | | Total | | |  |  |  |  |
|  |  |  |  |  |  | Open | | | Laparoscopic | | | |  |  |  |  |  |  |  |
| VTE | yes | Count | | | | 3 | | | 3 | | | | 6 | | |  |  |  |  |
|  |  | Expected Count | | | | 3.5 | | | 2.5 | | | | 6.0 | | |  |  |  |  |
|  |  | % within Procedure | | | | 6.1% | | | 8.3% | | | | 7.1% | | |  |  |  |  |
|  | no | Count | | | | 46 | | | 33 | | | | 79 | | |  |  |  |  |
|  |  | Expected Count | | | | 45.5 | | | 33.5 | | | | 79.0 | | |  |  |  |  |
|  |  | % within Procedure | | | | 93.9% | | | 91.7% | | | | 92.9% | | |  |  |  |  |
| Total | | Count | | | | 49 | | | 36 | | | | 85 | | |  |  |  |  |
|  |  | Expected Count | | | | 49.0 | | | 36.0 | | | | 85.0 | | |  |  |  |  |
|  |  | % within Procedure | | | | 100.0% | | | 100.0% | | | | 100.0% | | |  |  |  |  |
| **Chi-Square Tests** | | | | | | | | | | | | | | | | | | | |
|  | | | | Value | | | df | | | Asymp. Sig. (2-sided) | | | | Exact Sig. (2-sided) | | | Exact Sig. (1-sided) | | Point Probability |
| Pearson Chi-Square | | | | .155^a^ | | | 1 | | | .694 | | | | 1.000 | | | .506 | |  |
| Continuity Correction^b^ | | | | .000 | | | 1 | | | 1.000 | | | |  | | |  | |  |
| Likelihood Ratio | | | | .153 | | | 1 | | | .696 | | | | 1.000 | | | .506 | |  |
| Fisher's Exact Test | | | |  | | |  | | |  | | | | .695 | | | .506 | |  |
| Linear-by-Linear Association | | | | .153^c^ | | | 1 | | | .696 | | | | 1.000 | | | .506 | | .301 |
| N of Valid Cases | | | | 85 | | |  | | |  | | | |  | | |  | |  |
| a. 2 cells (50.0%) have expected count less than 5. The minimum expected count is 2.54. | | | | | | | | | | | | | | | | | | | |
| b. Computed only for a 2x2 table | | | | | | | | | | | | | | | | | | | |
| c. The standardized statistic is -.391. | | | | | | | | | | | | | | | | | | | |

| **Symmetric Measures** | | | | |
| --- | --- | --- | --- | --- |
|  | | Value | Approx. Sig. | Exact Sig. |
| Nominal by Nominal | Phi | -.043 | .694 | 1.000 |
|  | Cramer's V | .043 | .694 | 1.000 |
| N of Valid Cases | | 85 |  |  |


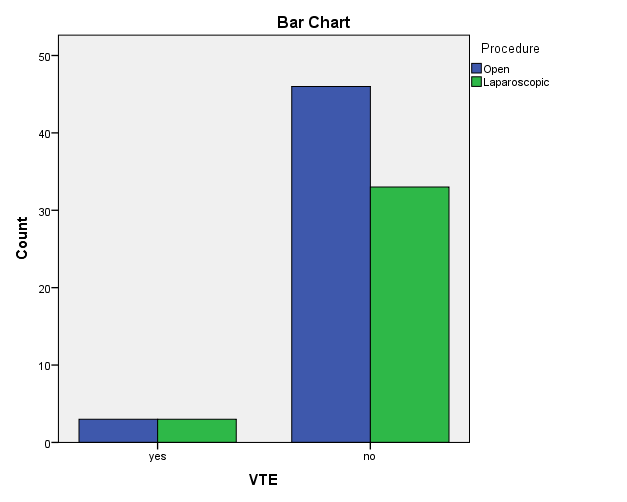


**Crosstabs**

| **Case Processing Summary** | | | | | | | | | | | | |
| --- | --- | --- | --- | --- | --- | --- | --- | --- | --- | --- | --- | --- |
|  | | | Cases | | | | | | | | | |
|  |  |  | Valid | | | Missing | | | | Total | | |
|  |  |  | N | Percent | | N | | Percent | | N | | Percent |
| VTE * Complexity | | | 85 | 100.0% | | 0 | | 0.0% | | 85 | | 100.0% |
| **VTE * Complexity Crosstabulation** | | | | | | | | | | |  |  |
|  | | | | | Complexity | | | | Total | |  |  |
|  |  |  |  |  | Simple | | Complex | |  |  |  |  |
| VTE | yes | Count | | | 4 | | 2 | | 6 | |  |  |
|  |  | Expected Count | | | 4.1 | | 1.9 | | 6.0 | |  |  |
|  |  | % within Complexity | | | 6.9% | | 7.4% | | 7.1% | |  |  |
|  | no | Count | | | 54 | | 25 | | 79 | |  |  |
|  |  | Expected Count | | | 53.9 | | 25.1 | | 79.0 | |  |  |
|  |  | % within Complexity | | | 93.1% | | 92.6% | | 92.9% | |  |  |
| Total | | Count | | | 58 | | 27 | | 85 | |  |  |
|  |  | Expected Count | | | 58.0 | | 27.0 | | 85.0 | |  |  |
|  |  | % within Complexity | | | 100.0% | | 100.0% | | 100.0% | |  |  |

| **Chi-Square Tests** | | | | | | |
| --- | --- | --- | --- | --- | --- | --- |
|  | Value | df | Asymp. Sig. (2-sided) | Exact Sig. (2-sided) | Exact Sig. (1-sided) | Point Probability |
| Pearson Chi-Square | .007^a^ | 1 | .932 | 1.000 | .625 |  |
| Continuity Correction^b^ | .000 | 1 | 1.000 |  |  |  |
| Likelihood Ratio | .007 | 1 | .932 | 1.000 | .625 |  |
| Fisher's Exact Test |  |  |  | 1.000 | .625 |  |
| Linear-by-Linear Association | .007^c^ | 1 | .932 | 1.000 | .625 | .340 |
| N of Valid Cases | 85 |  |  |  |  |  |
| a. 2 cells (50.0%) have expected count less than 5. The minimum expected count is 1.91. | | | | | | |
| b. Computed only for a 2x2 table | | | | | | |
| c. The standardized statistic is -.085. | | | | | | |

| **Symmetric Measures** | | | | |
| --- | --- | --- | --- | --- |
|  | | Value | Approx. Sig. | Exact Sig. |
| Nominal by Nominal | Phi | -.009 | .932 | 1.000 |
|  | Cramer's V | .009 | .932 | 1.000 |
| N of Valid Cases | | 85 |  |  |


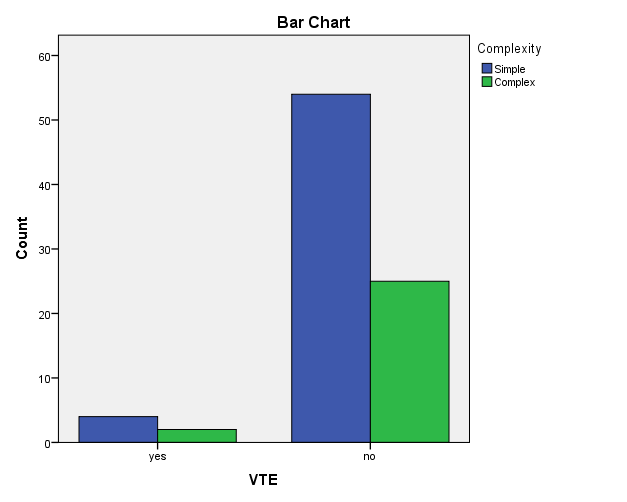


**Crosstabs**

| **Case Processing Summary** | | | | | | |
| --- | --- | --- | --- | --- | --- | --- |
|  | Cases | | | | | |
|  | Valid | | Missing | | Total | |
|  | N | Percent | N | Percent | N | Percent |
| VTE * Blood Tx | 85 | 100.0% | 0 | 0.0% | 85 | 100.0% |

| **VTE * Blood Tx Crosstabulation** | | | | | |
| --- | --- | --- | --- | --- | --- |
|  | | | Blood Tx | | Total |
|  |  |  | yes | no |  |
| VTE | yes | Count | 1 | 5 | 6 |
|  |  | Expected Count | 2.4 | 3.6 | 6.0 |
|  |  | % within Blood Tx | 2.9% | 9.8% | 7.1% |
|  | no | Count | 33 | 46 | 79 |
|  |  | Expected Count | 31.6 | 47.4 | 79.0 |
|  |  | % within Blood Tx | 97.1% | 90.2% | 92.9% |
| Total | | Count | 34 | 51 | 85 |
|  |  | Expected Count | 34.0 | 51.0 | 85.0 |
|  |  | % within Blood Tx | 100.0% | 100.0% | 100.0% |

| **Chi-Square Tests** | | | | | | |
| --- | --- | --- | --- | --- | --- | --- |
|  | Value | df | Asymp. Sig. (2-sided) | Exact Sig. (2-sided) | Exact Sig. (1-sided) | Point Probability |
| Pearson Chi-Square | 1.464^a^ | 1 | .226 | .395 | .224 |  |
| Continuity Correction^b^ | .605 | 1 | .437 |  |  |  |
| Likelihood Ratio | 1.637 | 1 | .201 | .395 | .224 |  |
| Fisher's Exact Test |  |  |  | .395 | .224 |  |
| Linear-by-Linear Association | 1.447^c^ | 1 | .229 | .395 | .224 | .183 |
| N of Valid Cases | 85 |  |  |  |  |  |
| a. 2 cells (50.0%) have expected count less than 5. The minimum expected count is 2.40. | | | | | | |
| b. Computed only for a 2x2 table | | | | | | |
| c. The standardized statistic is -1.203. | | | | | | |

| **Symmetric Measures** | | | | |
| --- | --- | --- | --- | --- |
|  | | Value | Approx. Sig. | Exact Sig. |
| Nominal by Nominal | Phi | -.131 | .226 | .395 |
|  | Cramer's V | .131 | .226 | .395 |
| N of Valid Cases | | 85 |  |  |


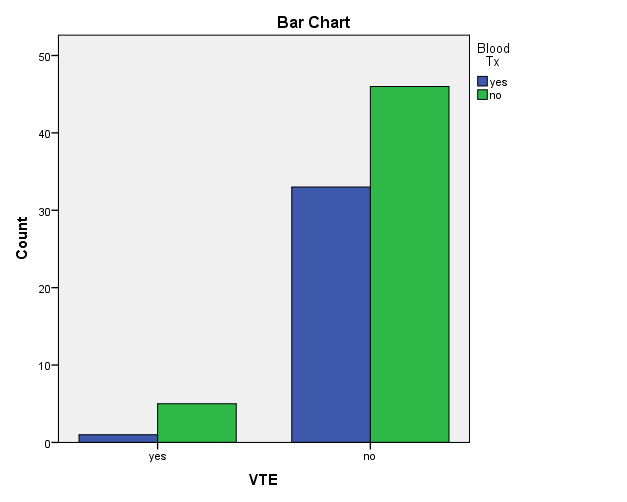


**Crosstabs**

| **Case Processing Summary** | | | | | | | | | | | | | | | | |  |  |
| --- | --- | --- | --- | --- | --- | --- | --- | --- | --- | --- | --- | --- | --- | --- | --- | --- | --- | --- |
|  | | Cases | | | | | | | | | | | | | | |  |  |
|  |  | Valid | | | | | Missing | | | | | | Total | | | |  |  |
|  |  | N | | Percent | | | N | | | Percent | | | N | | | Percent |  |  |
| VTE * Plts Tx | | 85 | | 100.0% | | | 0 | | | 0.0% | | | 85 | | | 100.0% |  |  |
| **VTE * Plts Tx Crosstabulation** | | | | | | | | | | | | | | |  |  |  |  |
|  | | | | | | Plts Tx | | | | | | Total | | |  |  |  |  |
|  |  |  |  |  |  | yes | | | no | | |  |  |  |  |  |  |  |
| VTE | yes | | Count | | | 0 | | | 6 | | | 6 | | |  |  |  |  |
|  |  |  | Expected Count | | | 1.1 | | | 4.9 | | | 6.0 | | |  |  |  |  |
|  |  |  | % within Plts Tx | | | 0.0% | | | 8.6% | | | 7.1% | | |  |  |  |  |
|  | no | | Count | | | 15 | | | 64 | | | 79 | | |  |  |  |  |
|  |  |  | Expected Count | | | 13.9 | | | 65.1 | | | 79.0 | | |  |  |  |  |
|  |  |  | % within Plts Tx | | | 100.0% | | | 91.4% | | | 92.9% | | |  |  |  |  |
| Total | | | Count | | | 15 | | | 70 | | | 85 | | |  |  |  |  |
|  |  |  | Expected Count | | | 15.0 | | | 70.0 | | | 85.0 | | |  |  |  |  |
|  |  |  | % within Plts Tx | | | 100.0% | | | 100.0% | | | 100.0% | | |  |  |  |  |
| **Chi-Square Tests** | | | | | | | | | | | | | | | | | | |
|  | | | | | Value | | | df | | | Asymp. Sig. (2-sided) | | | Exact Sig. (2-sided) | | | Exact Sig. (1-sided) | Point Probability |
| Pearson Chi-Square | | | | | 1.383^a^ | | | 1 | | | .240 | | | .365 | | | .300 |  |
| Continuity Correction^b^ | | | | | .385 | | | 1 | | | .535 | | |  | | |  |  |
| Likelihood Ratio | | | | | 2.426 | | | 1 | | | .119 | | | .365 | | | .300 |  |
| Fisher's Exact Test | | | | |  | | |  | | |  | | | .585 | | | .300 |  |
| Linear-by-Linear Association | | | | | 1.367^c^ | | | 1 | | | .242 | | | .365 | | | .300 | .300 |
| N of Valid Cases | | | | | 85 | | |  | | |  | | |  | | |  |  |
| a. 2 cells (50.0%) have expected count less than 5. The minimum expected count is 1.06. | | | | | | | | | | | | | | | | | | |
| b. Computed only for a 2x2 table | | | | | | | | | | | | | | | | | | |
| c. The standardized statistic is -1.169. | | | | | | | | | | | | | | | | | | |

| **Symmetric Measures** | | | | |
| --- | --- | --- | --- | --- |
|  | | Value | Approx. Sig. | Exact Sig. |
| Nominal by Nominal | Phi | -.128 | .240 | .365 |
|  | Cramer's V | .128 | .240 | .365 |
| N of Valid Cases | | 85 |  |  |


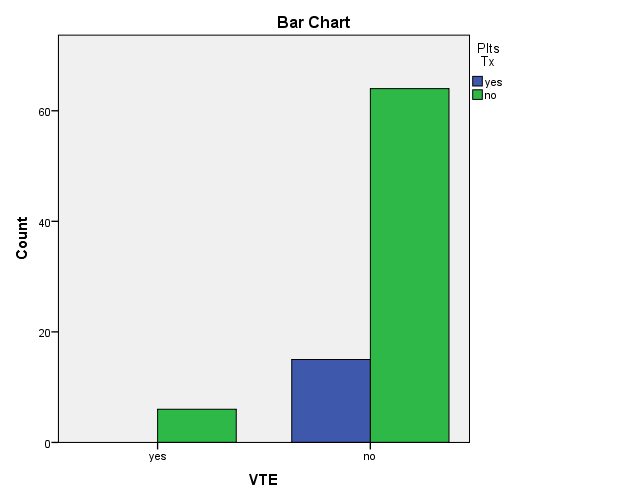


**Crosstabs**

| **Case Processing Summary** | | | | | | | | | | | | |
| --- | --- | --- | --- | --- | --- | --- | --- | --- | --- | --- | --- | --- |
|  | | Cases | | | | | | | | | | |
|  |  | Valid | | | | Missing | | | | Total | | |
|  |  | N | | Percent | | N | | Percent | | N | | Percent |
| VTE * Plasma | | 85 | | 100.0% | | 0 | | 0.0% | | 85 | | 100.0% |
| **VTE * Plasma Crosstabulation** | | | | | | | | | | |  |  |
|  | | | | | Plasma | | | | Total | |  |  |
|  |  |  |  |  | yes | | no | |  |  |  |  |
| VTE | yes | | Count | | 0 | | 6 | | 6 | |  |  |
|  |  |  | Expected Count | | 1.0 | | 5.0 | | 6.0 | |  |  |
|  |  |  | % within Plasma | | 0.0% | | 8.5% | | 7.1% | |  |  |
|  | no | | Count | | 14 | | 65 | | 79 | |  |  |
|  |  |  | Expected Count | | 13.0 | | 66.0 | | 79.0 | |  |  |
|  |  |  | % within Plasma | | 100.0% | | 91.5% | | 92.9% | |  |  |
| Total | | | Count | | 14 | | 71 | | 85 | |  |  |
|  |  |  | Expected Count | | 14.0 | | 71.0 | | 85.0 | |  |  |
|  |  |  | % within Plasma | | 100.0% | | 100.0% | | 100.0% | |  |  |

| **Chi-Square Tests** | | | | | | |
| --- | --- | --- | --- | --- | --- | --- |
|  | Value | df | Asymp. Sig. (2-sided) | Exact Sig. (2-sided) | Exact Sig. (1-sided) | Point Probability |
| Pearson Chi-Square | 1.273^a^ | 1 | .259 | .583 | .327 |  |
| Continuity Correction^b^ | .311 | 1 | .577 |  |  |  |
| Likelihood Ratio | 2.248 | 1 | .134 | .381 | .327 |  |
| Fisher's Exact Test |  |  |  | .583 | .327 |  |
| Linear-by-Linear Association | 1.258^c^ | 1 | .262 | .583 | .327 | .327 |
| N of Valid Cases | 85 |  |  |  |  |  |
| a. 1 cells (25.0%) have expected count less than 5. The minimum expected count is .99. | | | | | | |
| b. Computed only for a 2x2 table | | | | | | |
| c. The standardized statistic is -1.122. | | | | | | |

| **Symmetric Measures** | | | | |
| --- | --- | --- | --- | --- |
|  | | Value | Approx. Sig. | Exact Sig. |
| Nominal by Nominal | Phi | -.122 | .259 | .583 |
|  | Cramer's V | .122 | .259 | .583 |
| N of Valid Cases | | 85 |  |  |


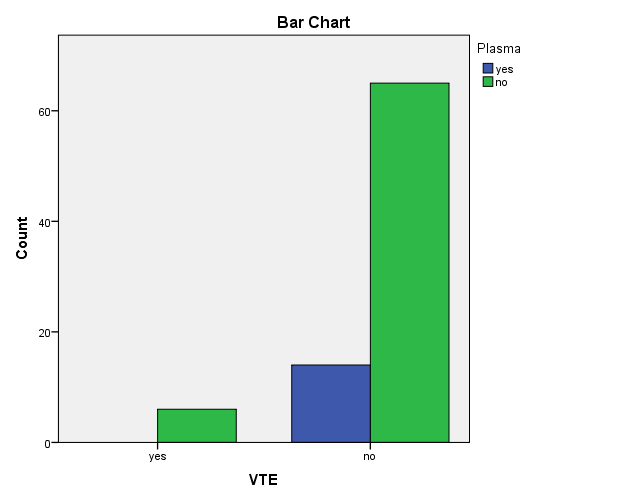


**Crosstabs**

| **Case Processing Summary** | | | | | | |
| --- | --- | --- | --- | --- | --- | --- |
|  | Cases | | | | | |
|  | Valid | | Missing | | Total | |
|  | N | Percent | N | Percent | N | Percent |
| VTE * Fibrinogen | 85 | 100.0% | 0 | 0.0% | 85 | 100.0% |

| **VTE * Fibrinogen Crosstabulation** | | | | | |
| --- | --- | --- | --- | --- | --- |
|  | | | Fibrinogen | | Total |
|  |  |  | yes | no |  |
| VTE | yes | Count | 0 | 6 | 6 |
|  |  | Expected Count | .2 | 5.8 | 6.0 |
|  |  | % within Fibrinogen | 0.0% | 7.3% | 7.1% |
|  | no | Count | 3 | 76 | 79 |
|  |  | Expected Count | 2.8 | 76.2 | 79.0 |
|  |  | % within Fibrinogen | 100.0% | 92.7% | 92.9% |
| Total | | Count | 3 | 82 | 85 |
|  |  | Expected Count | 3.0 | 82.0 | 85.0 |
|  |  | % within Fibrinogen | 100.0% | 100.0% | 100.0% |

| **Chi-Square Tests** | | | | | | |
| --- | --- | --- | --- | --- | --- | --- |
|  | Value | df | Asymp. Sig. (2-sided) | Exact Sig. (2-sided) | Exact Sig. (1-sided) | Point Probability |
| Pearson Chi-Square | .236^a^ | 1 | .627 | 1.000 | .801 |  |
| Continuity Correction^b^ | .000 | 1 | 1.000 |  |  |  |
| Likelihood Ratio | .447 | 1 | .504 | 1.000 | .801 |  |
| Fisher's Exact Test |  |  |  | 1.000 | .801 |  |
| Linear-by-Linear Association | .233^c^ | 1 | .629 | 1.000 | .801 | .801 |
| N of Valid Cases | 85 |  |  |  |  |  |
| a. 2 cells (50.0%) have expected count less than 5. The minimum expected count is .21. | | | | | | |
| b. Computed only for a 2x2 table | | | | | | |
| c. The standardized statistic is -.483. | | | | | | |

| **Symmetric Measures** | | | | |
| --- | --- | --- | --- | --- |
|  | | Value | Approx. Sig. | Exact Sig. |
| Nominal by Nominal | Phi | -.053 | .627 | 1.000 |
|  | Cramer's V | .053 | .627 | 1.000 |
| N of Valid Cases | | 85 |  |  |


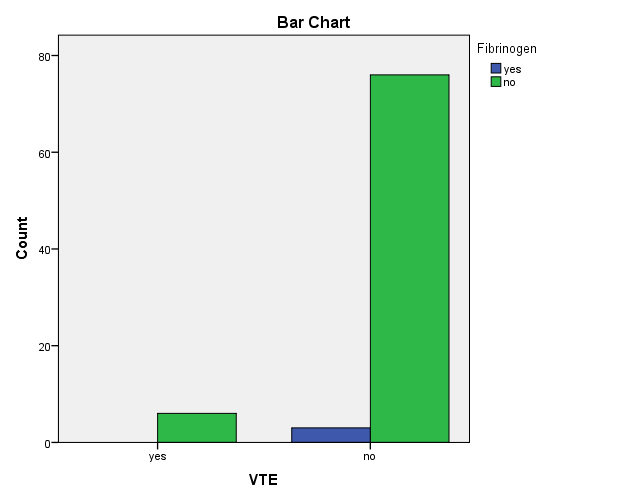


**Crosstabs**

| **Case Processing Summary** | | | | | | | | | | | | | | | | | |  |  |  |
| --- | --- | --- | --- | --- | --- | --- | --- | --- | --- | --- | --- | --- | --- | --- | --- | --- | --- | --- | --- | --- |
|  | | | Cases | | | | | | | | | | | | | | |  |  |  |
|  |  |  | Valid | | | | | Missing | | | | Total | | | | | |  |  |  |
|  |  |  | N | | Percent | | | N | | | Percent | N | | | Percent | | |  |  |  |
| VTE * Spleen size | | | 85 | | 100.0% | | | 0 | | | 0.0% | 85 | | | 100.0% | | |  |  |  |
| **VTE * Spleen size Crosstabulation** | | | | | | | | | | | | | | | | | | |  |  |
|  | | | | | | | Spleen size | | | | | | | | | | Total | |  |  |
|  |  |  |  |  |  |  | Normal | | | Splenomegaly | | | | Massive splenomegaly | | |  |  |  |  |
| VTE | yes | Count | | | | | 3 | | | 2 | | | | 1 | | | 6 | |  |  |
|  |  | Expected Count | | | | | 4.5 | | | 1.0 | | | | .5 | | | 6.0 | |  |  |
|  |  | % within Spleen size | | | | | 4.7% | | | 14.3% | | | | 14.3% | | | 7.1% | |  |  |
|  | no | Count | | | | | 61 | | | 12 | | | | 6 | | | 79 | |  |  |
|  |  | Expected Count | | | | | 59.5 | | | 13.0 | | | | 6.5 | | | 79.0 | |  |  |
|  |  | % within Spleen size | | | | | 95.3% | | | 85.7% | | | | 85.7% | | | 92.9% | |  |  |
| Total | | Count | | | | | 64 | | | 14 | | | | 7 | | | 85 | |  |  |
|  |  | Expected Count | | | | | 64.0 | | | 14.0 | | | | 7.0 | | | 85.0 | |  |  |
|  |  | % within Spleen size | | | | | 100.0% | | | 100.0% | | | | 100.0% | | | 100.0% | |  |  |
| **Chi-Square Tests** | | | | | | | | | | | | | | | | | | | | |
|  | | | | Value | | df | | | Asymp. Sig. (2-sided) | | | | Exact Sig. (2-sided) | | | Exact Sig. (1-sided) | | | | Point Probability |
| Pearson Chi-Square | | | | 2.220^a^ | | 2 | | | .330 | | | | .188 | | |  | | | |  |
| Likelihood Ratio | | | | 1.933 | | 2 | | | .380 | | | | .614 | | |  | | | |  |
| Fisher's Exact Test | | | | 3.075 | |  | | |  | | | | .188 | | |  | | | |  |
| Linear-by-Linear Association | | | | 1.882^b^ | | 1 | | | .170 | | | | .153 | | | .153 | | | | .096 |
| N of Valid Cases | | | | 85 | |  | | |  | | | |  | | |  | | | |  |
| a. 3 cells (50.0%) have expected count less than 5. The minimum expected count is .49. | | | | | | | | | | | | | | | | | | | | |
| b. The standardized statistic is -1.372. | | | | | | | | | | | | | | | | | | | | |

| **Symmetric Measures** | | | | |
| --- | --- | --- | --- | --- |
|  | | Value | Approx. Sig. | Exact Sig. |
| Nominal by Nominal | Phi | .162 | .330 | .188 |
|  | Cramer's V | .162 | .330 | .188 |
| N of Valid Cases | | 85 |  |  |


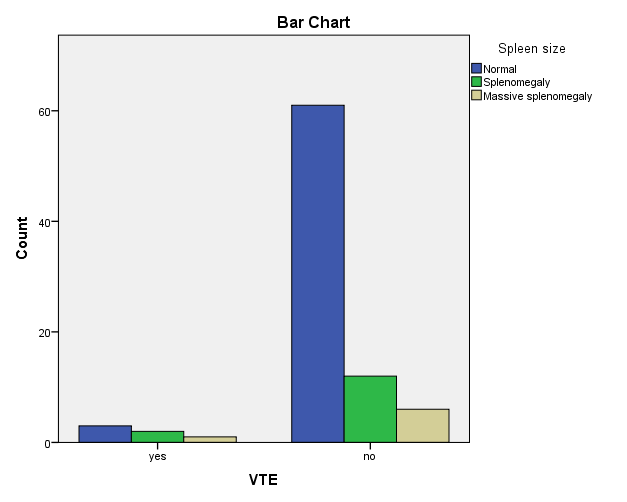


**Crosstabs**

| **Case Processing Summary** | | | | | | | | | | | | |
| --- | --- | --- | --- | --- | --- | --- | --- | --- | --- | --- | --- | --- |
|  | | | Cases | | | | | | | | | |
|  |  |  | Valid | | | Missing | | | | Total | | |
|  |  |  | N | Percent | | N | | Percent | | N | | Percent |
| VTE * Pathology | | | 85 | 100.0% | | 0 | | 0.0% | | 85 | | 100.0% |
| **VTE * Pathology Crosstabulation** | | | | | | | | | | |  |  |
|  | | | | | Pathology | | | | Total | |  |  |
|  |  |  |  |  | Benign | | Malignant | |  |  |  |  |
| VTE | yes | Count | | | 5 | | 1 | | 6 | |  |  |
|  |  | Expected Count | | | 5.1 | | .9 | | 6.0 | |  |  |
|  |  | % within Pathology | | | 6.9% | | 7.7% | | 7.1% | |  |  |
|  | no | Count | | | 67 | | 12 | | 79 | |  |  |
|  |  | Expected Count | | | 66.9 | | 12.1 | | 79.0 | |  |  |
|  |  | % within Pathology | | | 93.1% | | 92.3% | | 92.9% | |  |  |
| Total | | Count | | | 72 | | 13 | | 85 | |  |  |
|  |  | Expected Count | | | 72.0 | | 13.0 | | 85.0 | |  |  |
|  |  | % within Pathology | | | 100.0% | | 100.0% | | 100.0% | |  |  |

| **Chi-Square Tests** | | | | | | |
| --- | --- | --- | --- | --- | --- | --- |
|  | Value | df | Asymp. Sig. (2-sided) | Exact Sig. (2-sided) | Exact Sig. (1-sided) | Point Probability |
| Pearson Chi-Square | .009^a^ | 1 | .923 | 1.000 | .643 |  |
| Continuity Correction^b^ | .000 | 1 | 1.000 |  |  |  |
| Likelihood Ratio | .009 | 1 | .924 | 1.000 | .643 |  |
| Fisher's Exact Test |  |  |  | 1.000 | .643 |  |
| Linear-by-Linear Association | .009^c^ | 1 | .923 | 1.000 | .643 | .416 |
| N of Valid Cases | 85 |  |  |  |  |  |
| a. 1 cells (25.0%) have expected count less than 5. The minimum expected count is .92. | | | | | | |
| b. Computed only for a 2x2 table | | | | | | |
| c. The standardized statistic is -.096. | | | | | | |

| **Symmetric Measures** | | | | |
| --- | --- | --- | --- | --- |
|  | | Value | Approx. Sig. | Exact Sig. |
| Nominal by Nominal | Phi | -.011 | .923 | 1.000 |
|  | Cramer's V | .011 | .923 | 1.000 |
| N of Valid Cases | | 85 |  |  |


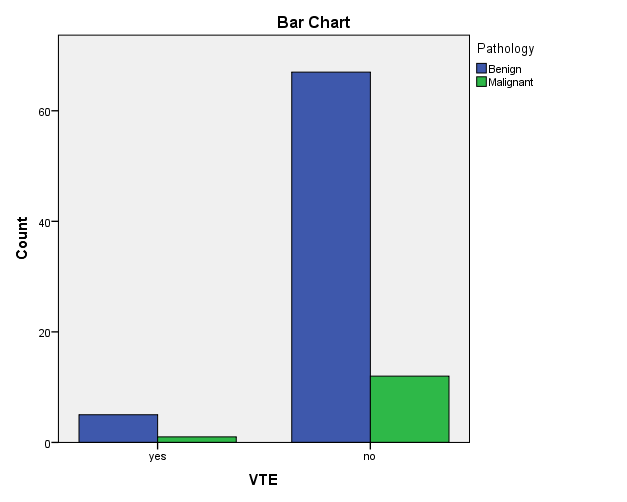


**Crosstabs**

| **Case Processing Summary** | | | | | | |
| --- | --- | --- | --- | --- | --- | --- |
|  | Cases | | | | | |
|  | Valid | | Missing | | Total | |
|  | N | Percent | N | Percent | N | Percent |
| VTE * Prophylaxis AC | 85 | 100.0% | 0 | 0.0% | 85 | 100.0% |

| **VTE * Prophylaxis AC Crosstabulation** | | | | | | | | | | | | | | |  |  |  |
| --- | --- | --- | --- | --- | --- | --- | --- | --- | --- | --- | --- | --- | --- | --- | --- | --- | --- |
|  | | | | | | | Prophylaxis AC | | | | | Total | | |  |  |  |
|  |  |  |  |  |  |  | yes | | | no | |  |  |  |  |  |  |
| VTE | yes | Count | | | | | 5 | | | 1 | | 6 | | |  |  |  |
|  |  | Expected Count | | | | | 4.7 | | | 1.3 | | 6.0 | | |  |  |  |
|  |  | % within Prophylaxis AC | | | | | 7.5% | | | 5.6% | | 7.1% | | |  |  |  |
|  | no | Count | | | | | 62 | | | 17 | | 79 | | |  |  |  |
|  |  | Expected Count | | | | | 62.3 | | | 16.7 | | 79.0 | | |  |  |  |
|  |  | % within Prophylaxis AC | | | | | 92.5% | | | 94.4% | | 92.9% | | |  |  |  |
| Total | | Count | | | | | 67 | | | 18 | | 85 | | |  |  |  |
|  |  | Expected Count | | | | | 67.0 | | | 18.0 | | 85.0 | | |  |  |  |
|  |  | % within Prophylaxis AC | | | | | 100.0% | | | 100.0% | | 100.0% | | |  |  |  |
| **Chi-Square Tests** | | | | | | | | | | | | | | | | | |
|  | | | | Value | | df | | | Asymp. Sig. (2-sided) | | | | Exact Sig. (2-sided) | | | Exact Sig. (1-sided) | Point Probability |
| Pearson Chi-Square | | | | .079^a^ | | 1 | | | .779 | | | | 1.000 | | | .626 |  |
| Continuity Correction^b^ | | | | .000 | | 1 | | | 1.000 | | | |  | | |  |  |
| Likelihood Ratio | | | | .083 | | 1 | | | .773 | | | | 1.000 | | | .626 |  |
| Fisher's Exact Test | | | |  | |  | | |  | | | | 1.000 | | | .626 |  |
| Linear-by-Linear Association | | | | .078^c^ | | 1 | | | .780 | | | | 1.000 | | | .626 | .397 |
| N of Valid Cases | | | | 85 | |  | | |  | | | |  | | |  |  |
| a. 2 cells (50.0%) have expected count less than 5. The minimum expected count is 1.27. | | | | | | | | | | | | | | | | | |
| b. Computed only for a 2x2 table | | | | | | | | | | | | | | | | | |
| c. The standardized statistic is .279. | | | | | | | | | | | | | | | | | |
| **Symmetric Measures** | | | | | | | | | | | | | |  |  |  |  |
|  | | | | | Value | | | Approx. Sig. | | | Exact Sig. | | |  |  |  |  |
| Nominal by Nominal | | | Phi | | .030 | | | .779 | | | 1.000 | | |  |  |  |  |
|  |  |  | Cramer's V | | .030 | | | .779 | | | 1.000 | | |  |  |  |  |
| N of Valid Cases | | | | | 85 | | |  | | |  | | |  |  |  |  |


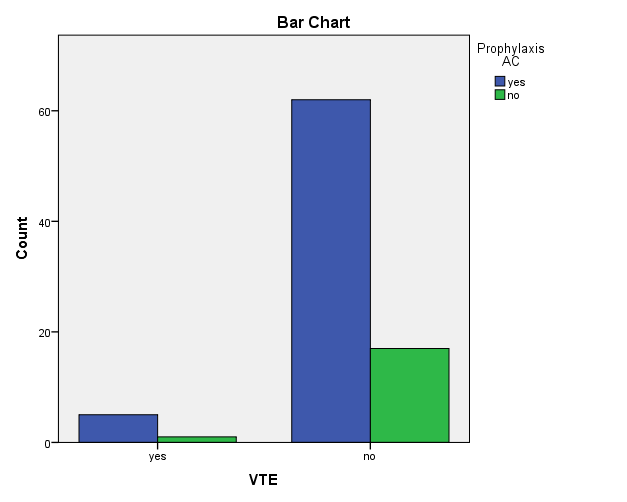


**Crosstabs**

| **Case Processing Summary** | | | | | | | | | | | | | | | | | | | |  |  |
| --- | --- | --- | --- | --- | --- | --- | --- | --- | --- | --- | --- | --- | --- | --- | --- | --- | --- | --- | --- | --- | --- |
|  | | | | Cases | | | | | | | | | | | | | | | |  |  |
|  |  |  |  | Valid | | | | | Missing | | | | | Total | | | | | |  |  |
|  |  |  |  | N | | Percent | | | N | | Percent | | | N | | | | Percent | |  |  |
| VTE * Wound complication/Collection | | | | 85 | | 100.0% | | | 0 | | 0.0% | | | 85 | | | | 100.0% | |  |  |
| **VTE * Wound complication/Collection Crosstabulation** | | | | | | | | | | | | | | | | | | |  |  |  |
|  | | | | | | | Wound complication/Collection | | | | | | | | | Total | | |  |  |  |
|  |  |  |  |  |  |  | yes | | | no | | | | | |  |  |  |  |  |  |
| VTE | yes | Count | | | | | 1 | | | 5 | | | | | | 6 | | |  |  |  |
|  |  | Expected Count | | | | | 1.3 | | | 4.7 | | | | | | 6.0 | | |  |  |  |
|  |  | % within Wound complication/Collection | | | | | 5.3% | | | 7.6% | | | | | | 7.1% | | |  |  |  |
|  | no | Count | | | | | 18 | | | 61 | | | | | | 79 | | |  |  |  |
|  |  | Expected Count | | | | | 17.7 | | | 61.3 | | | | | | 79.0 | | |  |  |  |
|  |  | % within Wound complication/Collection | | | | | 94.7% | | | 92.4% | | | | | | 92.9% | | |  |  |  |
| Total | | Count | | | | | 19 | | | 66 | | | | | | 85 | | |  |  |  |
|  |  | Expected Count | | | | | 19.0 | | | 66.0 | | | | | | 85.0 | | |  |  |  |
|  |  | % within Wound complication/Collection | | | | | 100.0% | | | 100.0% | | | | | | 100.0% | | |  |  |  |
| **Chi-Square Tests** | | | | | | | | | | | | | | | | | | | | | |
|  | | | | Value | | df | | | Asymp. Sig. (2-sided) | | | | Exact Sig. (2-sided) | | | | Exact Sig. (1-sided) | | | | Point Probability |
| Pearson Chi-Square | | | | .120^a^ | | 1 | | | .729 | | | | 1.000 | | | | .596 | | | |  |
| Continuity Correction^b^ | | | | .000 | | 1 | | | 1.000 | | | |  | | | |  | | | |  |
| Likelihood Ratio | | | | .128 | | 1 | | | .720 | | | | 1.000 | | | | .596 | | | |  |
| Fisher's Exact Test | | | |  | |  | | |  | | | | 1.000 | | | | .596 | | | |  |
| Linear-by-Linear Association | | | | .119^c^ | | 1 | | | .730 | | | | 1.000 | | | | .596 | | | | .388 |
| N of Valid Cases | | | | 85 | |  | | |  | | | |  | | | |  | | | |  |
| a. 2 cells (50.0%) have expected count less than 5. The minimum expected count is 1.34. | | | | | | | | | | | | | | | | | | | | | |
| b. Computed only for a 2x2 table | | | | | | | | | | | | | | | | | | | | | |
| c. The standardized statistic is -.345. | | | | | | | | | | | | | | | | | | | | | |
| **Symmetric Measures** | | | | | | | | | | | | | | |  |  |  |  |  |  |  |
|  | | | | | Value | | | Approx. Sig. | | | | Exact Sig. | | |  |  |  |  |  |  |  |
| Nominal by Nominal | | | Phi | | -.038 | | | .729 | | | | 1.000 | | |  |  |  |  |  |  |  |
|  |  |  | Cramer's V | | .038 | | | .729 | | | | 1.000 | | |  |  |  |  |  |  |  |
| N of Valid Cases | | | | | 85 | | |  | | | |  | | |  |  |  |  |  |  |  |


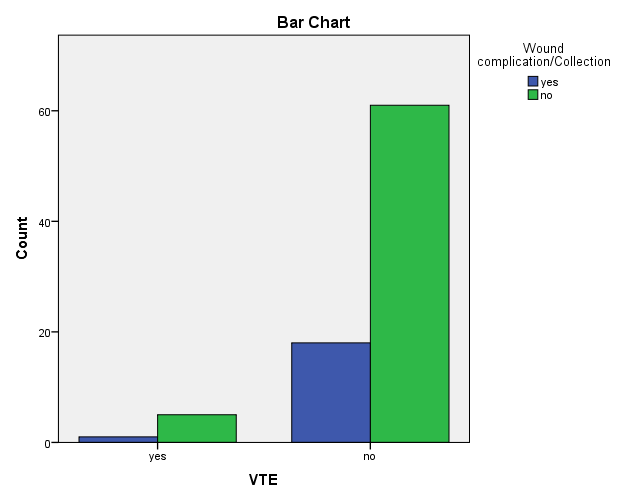


**Crosstabs**

| **Case Processing Summary** | | | | | | | | | | | | | | | |  |  |
| --- | --- | --- | --- | --- | --- | --- | --- | --- | --- | --- | --- | --- | --- | --- | --- | --- | --- |
|  | | | Cases | | | | | | | | | | | | |  |  |
|  |  |  | Valid | | | Missing | | | | | Total | | | | |  |  |
|  |  |  | N | Percent | | N | | Percent | | | N | | | Percent | |  |  |
| VTE * Resurgery / Drainage | | | 85 | 100.0% | | 0 | | 0.0% | | | 85 | | | 100.0% | |  |  |
| **VTE * Resurgery / Drainage Crosstabulation** | | | | | | | | | | | | | | |  |  |  |
|  | | | | | Resurgery / Drainage | | | | | | | Total | | |  |  |  |
|  |  |  |  |  | Re-surgery | | Drainage | | | None | |  |  |  |  |  |  |
| VTE | yes | Count | | | 1 | | 1 | | | 4 | | 6 | | |  |  |  |
|  |  | Expected Count | | | .6 | | .5 | | | 4.9 | | 6.0 | | |  |  |  |
|  |  | % within Resurgery / Drainage | | | 12.5% | | 14.3% | | | 5.7% | | 7.1% | | |  |  |  |
|  | no | Count | | | 7 | | 6 | | | 66 | | 79 | | |  |  |  |
|  |  | Expected Count | | | 7.4 | | 6.5 | | | 65.1 | | 79.0 | | |  |  |  |
|  |  | % within Resurgery / Drainage | | | 87.5% | | 85.7% | | | 94.3% | | 92.9% | | |  |  |  |
| Total | | Count | | | 8 | | 7 | | | 70 | | 85 | | |  |  |  |
|  |  | Expected Count | | | 8.0 | | 7.0 | | | 70.0 | | 85.0 | | |  |  |  |
|  |  | % within Resurgery / Drainage | | | 100.0% | | 100.0% | | | 100.0% | | 100.0% | | |  |  |  |
| **Chi-Square Tests** | | | | | | | | | | | | | | | | | |
|  | | | Value | df | | Asymp. Sig. (2-sided) | | | Exact Sig. (2-sided) | | | | Exact Sig. (1-sided) | | | | Point Probability |
| Pearson Chi-Square | | | 1.111^a^ | 2 | | .574 | | | .779 | | | |  | | | |  |
| Likelihood Ratio | | | .942 | 2 | | .624 | | | 1.000 | | | |  | | | |  |
| Fisher's Exact Test | | | 2.226 |  | |  | | | .285 | | | |  | | | |  |
| Linear-by-Linear Association | | | .871^b^ | 1 | | .351 | | | .541 | | | | .241 | | | | .122 |
| N of Valid Cases | | | 85 |  | |  | | |  | | | |  | | | |  |
| a. 3 cells (50.0%) have expected count less than 5. The minimum expected count is .49. | | | | | | | | | | | | | | | | | |
| b. The standardized statistic is .933. | | | | | | | | | | | | | | | | | |

| **Symmetric Measures** | | | | |
| --- | --- | --- | --- | --- |
|  | | Value | Approx. Sig. | Exact Sig. |
| Nominal by Nominal | Phi | .114 | .574 | .779 |
|  | Cramer's V | .114 | .574 | .779 |
| N of Valid Cases | | 85 |  |  |


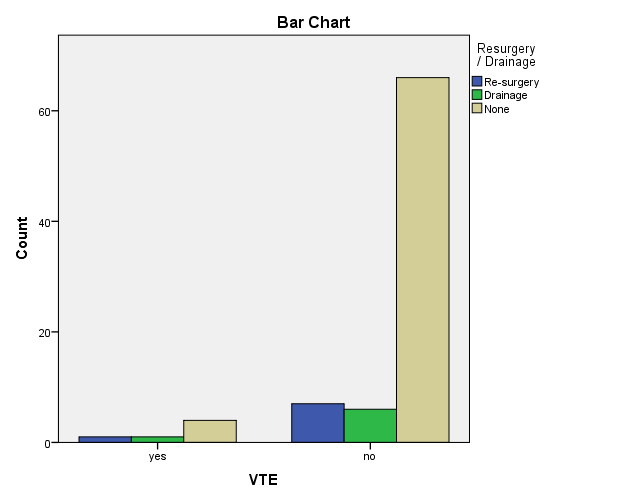


**Crosstabs**

| **Case Processing Summary** | | | | | | | | | | | | | |
| --- | --- | --- | --- | --- | --- | --- | --- | --- | --- | --- | --- | --- | --- |
|  | | | Cases | | | | | | | | | | |
|  |  |  | Valid | | | Missing | | | | Total | | | |
|  |  |  | N | Percent | | N | | Percent | | N | | Percent | |
| VTE * Anticoagulation | | | 85 | 100.0% | | 0 | | 0.0% | | 85 | | 100.0% | |
| **VTE * Anticoagulation Crosstabulation** | | | | | | | | | | | | |  |
|  | | | | | Anticoagulation | | | | | | Total | |  |
|  |  |  |  |  | yes | | no | | N/A | |  |  |  |
| VTE | yes | Count | | | 0 | | 4 | | 2 | | 6 | |  |
|  |  | Expected Count | | | .6 | | 5.3 | | .1 | | 6.0 | |  |
|  |  | % within Anticoagulation | | | 0.0% | | 5.3% | | 100.0% | | 7.1% | |  |
|  | no | Count | | | 8 | | 71 | | 0 | | 79 | |  |
|  |  | Expected Count | | | 7.4 | | 69.7 | | 1.9 | | 79.0 | |  |
|  |  | % within Anticoagulation | | | 100.0% | | 94.7% | | 0.0% | | 92.9% | |  |
| Total | | Count | | | 8 | | 75 | | 2 | | 85 | |  |
|  |  | Expected Count | | | 8.0 | | 75.0 | | 2.0 | | 85.0 | |  |
|  |  | % within Anticoagulation | | | 100.0% | | 100.0% | | 100.0% | | 100.0% | |  |

| **Chi-Square Tests** | | | | | | |
| --- | --- | --- | --- | --- | --- | --- |
|  | Value | df | Asymp. Sig. (2-sided) | Exact Sig. (2-sided) | Exact Sig. (1-sided) | Point Probability |
| Pearson Chi-Square | 27.281^a^ | 2 | .000 | .004 |  |  |
| Likelihood Ratio | 12.145 | 2 | .002 | .005 |  |  |
| Fisher's Exact Test | 10.896 |  |  | .005 |  |  |
| Linear-by-Linear Association | 9.239^b^ | 1 | .002 | .012 | .003 | .003 |
| N of Valid Cases | 85 |  |  |  |  |  |
| a. 3 cells (50.0%) have expected count less than 5. The minimum expected count is .14. | | | | | | |
| b. The standardized statistic is -3.040. | | | | | | |

| **Symmetric Measures** | | | | |
| --- | --- | --- | --- | --- |
|  | | Value | Approx. Sig. | Exact Sig. |
| Nominal by Nominal | Phi | .567 | .000 | .004 |
|  | Cramer's V | .567 | .000 | .004 |
| N of Valid Cases | | 85 |  |  |


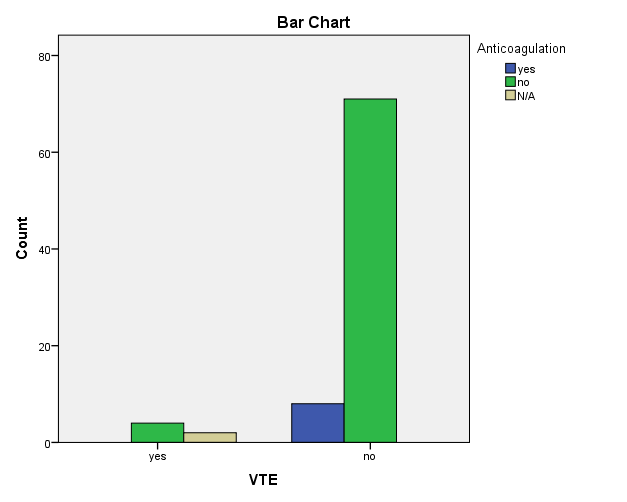


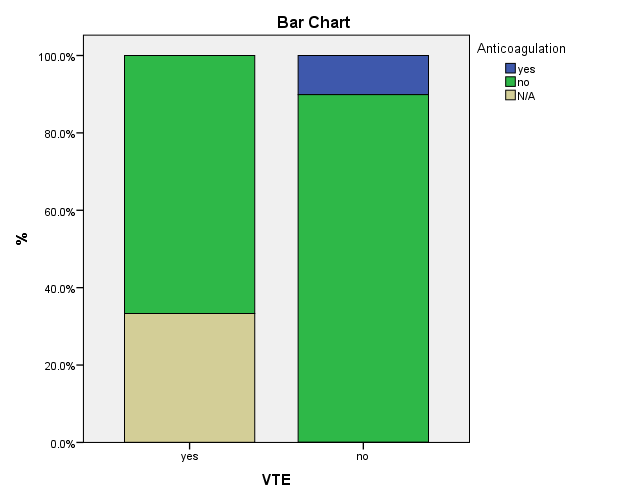


**Crosstabs**

| **Case Processing Summary** | | | | | | | | | | | | | | | | | |  |  |
| --- | --- | --- | --- | --- | --- | --- | --- | --- | --- | --- | --- | --- | --- | --- | --- | --- | --- | --- | --- |
|  | | | Cases | | | | | | | | | | | | | | |  |  |
|  |  |  | Valid | | | | | Missing | | | | | Total | | | | |  |  |
|  |  |  | N | | Percent | | | N | | | Percent | | N | | | Percent | |  |  |
| VTE * Antiplatelets | | | 85 | | 100.0% | | | 0 | | | 0.0% | | 85 | | | 100.0% | |  |  |
| **VTE * Antiplatelets Crosstabulation** | | | | | | | | | | | | | | |  |  |  |  |  |
|  | | | | | | | Antiplatelets | | | | | Total | | |  |  |  |  |  |
|  |  |  |  |  |  |  | yes | | | no | |  |  |  |  |  |  |  |  |
| VTE | yes | Count | | | | | 1 | | | 5 | | 6 | | |  |  |  |  |  |
|  |  | Expected Count | | | | | 1.2 | | | 4.8 | | 6.0 | | |  |  |  |  |  |
|  |  | % within Antiplatelets | | | | | 5.9% | | | 7.4% | | 7.1% | | |  |  |  |  |  |
|  | no | Count | | | | | 16 | | | 63 | | 79 | | |  |  |  |  |  |
|  |  | Expected Count | | | | | 15.8 | | | 63.2 | | 79.0 | | |  |  |  |  |  |
|  |  | % within Antiplatelets | | | | | 94.1% | | | 92.6% | | 92.9% | | |  |  |  |  |  |
| Total | | Count | | | | | 17 | | | 68 | | 85 | | |  |  |  |  |  |
|  |  | Expected Count | | | | | 17.0 | | | 68.0 | | 85.0 | | |  |  |  |  |  |
|  |  | % within Antiplatelets | | | | | 100.0% | | | 100.0% | | 100.0% | | |  |  |  |  |  |
| **Chi-Square Tests** | | | | | | | | | | | | | | | | | | | |
|  | | | | Value | | df | | | Asymp. Sig. (2-sided) | | | | | Exact Sig. (2-sided) | | | Exact Sig. (1-sided) | | Point Probability |
| Pearson Chi-Square | | | | .045^a^ | | 1 | | | .832 | | | | | 1.000 | | | .655 | |  |
| Continuity Correction^b^ | | | | .000 | | 1 | | | 1.000 | | | | |  | | |  | |  |
| Likelihood Ratio | | | | .047 | | 1 | | | .829 | | | | | 1.000 | | | .655 | |  |
| Fisher's Exact Test | | | |  | |  | | |  | | | | | 1.000 | | | .655 | |  |
| Linear-by-Linear Association | | | | .044^c^ | | 1 | | | .833 | | | | | 1.000 | | | .655 | | .405 |
| N of Valid Cases | | | | 85 | |  | | |  | | | | |  | | |  | |  |
| a. 2 cells (50.0%) have expected count less than 5. The minimum expected count is 1.20. | | | | | | | | | | | | | | | | | | | |
| b. Computed only for a 2x2 table | | | | | | | | | | | | | | | | | | | |
| c. The standardized statistic is -.210. | | | | | | | | | | | | | | | | | | | |

| **Symmetric Measures** | | | | |
| --- | --- | --- | --- | --- |
|  | | Value | Approx. Sig. | Exact Sig. |
| Nominal by Nominal | Phi | -.023 | .832 | 1.000 |
|  | Cramer's V | .023 | .832 | 1.000 |
| N of Valid Cases | | 85 |  |  |


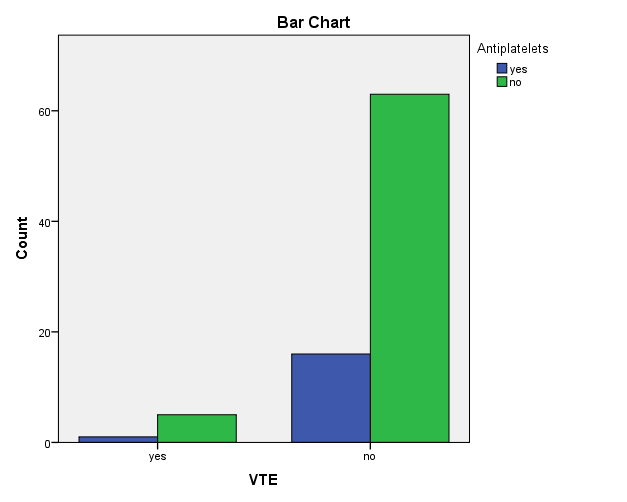


**Crosstabs**

| **Case Processing Summary** | | | | | | | | | | | | |
| --- | --- | --- | --- | --- | --- | --- | --- | --- | --- | --- | --- | --- |
|  | | | Cases | | | | | | | | | |
|  |  |  | Valid | | | Missing | | | | Total | | |
|  |  |  | N | Percent | | N | | Percent | | N | | Percent |
| VTE * Penicillin | | | 85 | 100.0% | | 0 | | 0.0% | | 85 | | 100.0% |
| **VTE * Penicillin Crosstabulation** | | | | | | | | | | |  |  |
|  | | | | | Penicillin | | | | Total | |  |  |
|  |  |  |  |  | yes | | no | |  |  |  |  |
| VTE | yes | Count | | | 5 | | 1 | | 6 | |  |  |
|  |  | Expected Count | | | 5.9 | | .1 | | 6.0 | |  |  |
|  |  | % within Penicillin | | | 6.0% | | 50.0% | | 7.1% | |  |  |
|  | no | Count | | | 78 | | 1 | | 79 | |  |  |
|  |  | Expected Count | | | 77.1 | | 1.9 | | 79.0 | |  |  |
|  |  | % within Penicillin | | | 94.0% | | 50.0% | | 92.9% | |  |  |
| Total | | Count | | | 83 | | 2 | | 85 | |  |  |
|  |  | Expected Count | | | 83.0 | | 2.0 | | 85.0 | |  |  |
|  |  | % within Penicillin | | | 100.0% | | 100.0% | | 100.0% | |  |  |

| **Chi-Square Tests** | | | | | | |
| --- | --- | --- | --- | --- | --- | --- |
|  | Value | df | Asymp. Sig. (2-sided) | Exact Sig. (2-sided) | Exact Sig. (1-sided) | Point Probability |
| Pearson Chi-Square | 5.757^a^ | 1 | .016 | .137 | .137 |  |
| Continuity Correction^b^ | 1.005 | 1 | .316 |  |  |  |
| Likelihood Ratio | 2.818 | 1 | .093 | .137 | .137 |  |
| Fisher's Exact Test |  |  |  | .137 | .137 |  |
| Linear-by-Linear Association | 5.689^c^ | 1 | .017 | .137 | .137 | .133 |
| N of Valid Cases | 85 |  |  |  |  |  |
| a. 2 cells (50.0%) have expected count less than 5. The minimum expected count is .14. | | | | | | |
| b. Computed only for a 2x2 table | | | | | | |
| c. The standardized statistic is -2.385. | | | | | | |

| **Symmetric Measures** | | | | |
| --- | --- | --- | --- | --- |
|  | | Value | Approx. Sig. | Exact Sig. |
| Nominal by Nominal | Phi | -.260 | .016 | .137 |
|  | Cramer's V | .260 | .016 | .137 |
| N of Valid Cases | | 85 |  |  |


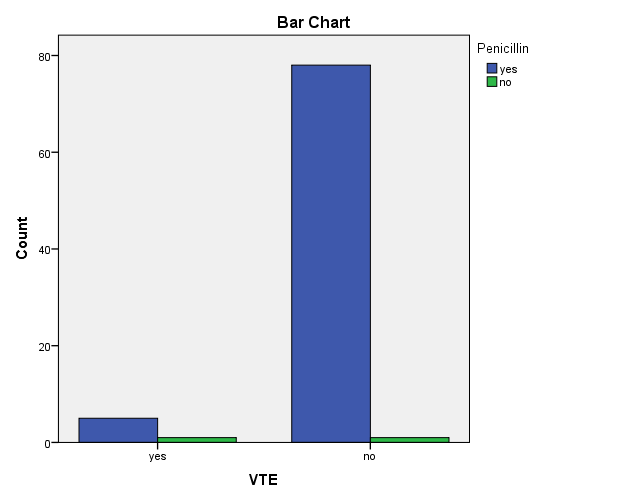


**Crosstabs**

| **Case Processing Summary** | | | | | | |
| --- | --- | --- | --- | --- | --- | --- |
|  | Cases | | | | | |
|  | Valid | | Missing | | Total | |
|  | N | Percent | N | Percent | N | Percent |
| VTE * Post -op PLT | 85 | 100.0% | 0 | 0.0% | 85 | 100.0% |

| **VTE * Post -op PLT Crosstabulation** | | | | | | |
| --- | --- | --- | --- | --- | --- | --- |
|  | | | Post -op PLT | | | Total |
|  |  |  | <450 x 10^3 | >450 x 10^3 | >1000 x 10^3 |  |
| VTE | yes | Count | 2 | 3 | 1 | 6 |
|  |  | Expected Count | 1.6 | 2.9 | 1.5 | 6.0 |
|  |  | % within Post -op PLT | 8.7% | 7.3% | 4.8% | 7.1% |
|  | no | Count | 21 | 38 | 20 | 79 |
|  |  | Expected Count | 21.4 | 38.1 | 19.5 | 79.0 |
|  |  | % within Post -op PLT | 91.3% | 92.7% | 95.2% | 92.9% |
| Total | | Count | 23 | 41 | 21 | 85 |
|  |  | Expected Count | 23.0 | 41.0 | 21.0 | 85.0 |
|  |  | % within Post -op PLT | 100.0% | 100.0% | 100.0% | 100.0% |

| **Chi-Square Tests** | | | | | | |
| --- | --- | --- | --- | --- | --- | --- |
|  | Value | df | Asymp. Sig. (2-sided) | Exact Sig. (2-sided) | Exact Sig. (1-sided) | Point Probability |
| Pearson Chi-Square | .267^a^ | 2 | .875 | 1.000 |  |  |
| Likelihood Ratio | .281 | 2 | .869 | 1.000 |  |  |
| Fisher's Exact Test | .410 |  |  | 1.000 |  |  |
| Linear-by-Linear Association | .253^b^ | 1 | .615 | .773 | .419 | .204 |
| N of Valid Cases | 85 |  |  |  |  |  |
| a. 3 cells (50.0%) have expected count less than 5. The minimum expected count is 1.48. | | | | | | |
| b. The standardized statistic is .503. | | | | | | |

| **Symmetric Measures** | | | | |
| --- | --- | --- | --- | --- |
|  | | Value | Approx. Sig. | Exact Sig. |
| Nominal by Nominal | Phi | .056 | .875 | 1.000 |
|  | Cramer's V | .056 | .875 | 1.000 |
| N of Valid Cases | | 85 |  |  |


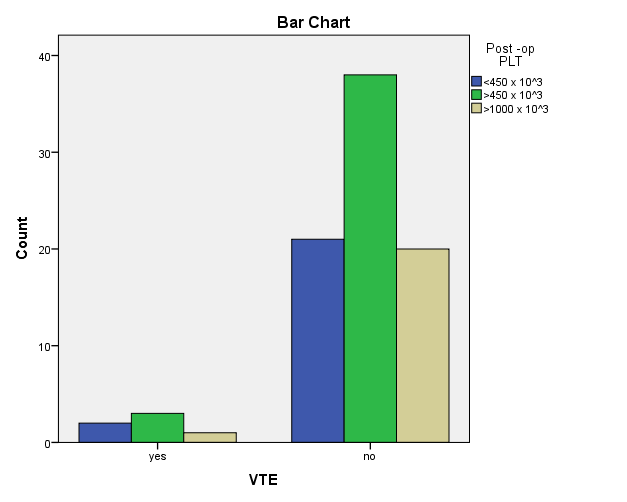


**Nonparametric Tests**


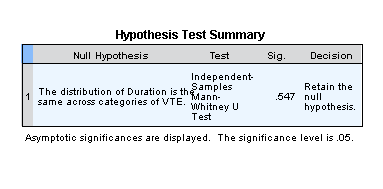


**Explore**

**VTE**

| **Case Processing Summary** | | | | | | | | | | | | | |  |  |  |  |
| --- | --- | --- | --- | --- | --- | --- | --- | --- | --- | --- | --- | --- | --- | --- | --- | --- | --- |
|  | VTE | Cases | | | | | | | | | | | |  |  |  |  |
|  |  | Valid | | | | Missing | | | | Total | | | |  |  |  |  |
|  |  | N | | Percent | | N | | Percent | | N | | Percent | |  |  |  |  |
| Duration | yes | 6 | | 100.0% | | 0 | | 0.0% | | 6 | | 100.0% | |  |  |  |  |
|  | no | 78 | | 98.7% | | 1 | | 1.3% | | 79 | | 100.0% | |  |  |  |  |
| **Percentiles** | | | | | | | | | | | | | | | | | |
|  | | |  | | VTE | | Percentiles | | | | | | | | | | |
|  | | |  | |  |  | 5 | | 10 | | 25 | | 50 | | 75 | 90 | 95 |
| Weighted Average(Definition 1) | | | Duration | | yes | | 2.5000 | | 2.5000 | | 2.5000 | | 2.8750 | | 4.0625 | . | . |
|  |  |  |  |  | no | | 1.2375 | | 1.7250 | | 2.0000 | | 2.8750 | | 3.5000 | 4.7500 | 5.0625 |
| Tukey's Hinges | | | Duration | | yes | |  | |  | | 2.5000 | | 2.8750 | | 4.0000 |  |  |
|  |  |  |  |  | no | |  | |  | | 2.0000 | | 2.8750 | | 3.5000 |  |  |

**Duration**


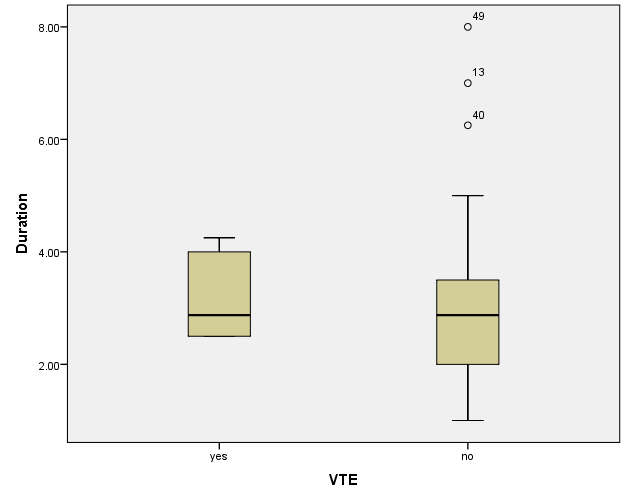


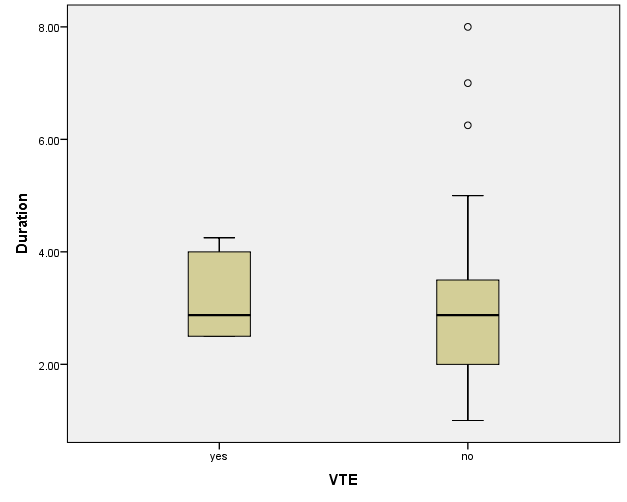


**Nonparametric Tests**


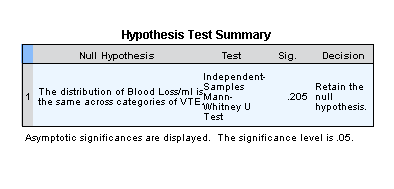


**Explore**

**VTE**

| **Case Processing Summary** | | | | | | | | | | | | | |  |  |  |  |
| --- | --- | --- | --- | --- | --- | --- | --- | --- | --- | --- | --- | --- | --- | --- | --- | --- | --- |
|  | VTE | Cases | | | | | | | | | | | |  |  |  |  |
|  |  | Valid | | | | Missing | | | | Total | | | |  |  |  |  |
|  |  | N | | Percent | | N | | Percent | | N | | Percent | |  |  |  |  |
| Blood Loss/ml | yes | 6 | | 100.0% | | 0 | | 0.0% | | 6 | | 100.0% | |  |  |  |  |
|  | no | 78 | | 98.7% | | 1 | | 1.3% | | 79 | | 100.0% | |  |  |  |  |
| **Percentiles** | | | | | | | | | | | | | | | | | |
|  | | |  | | VTE | | Percentiles | | | | | | | | | | |
|  | | |  | |  |  | 5 | | 10 | | 25 | | 50 | | 75 | 90 | 95 |
| Weighted Average(Definition 1) | | | Blood Loss/ml | | yes | | 50.00 | | 50.00 | | 50.00 | | 300.00 | | 650.00 | . | . |
|  |  |  |  |  | no | | 50.00 | | 50.00 | | 80.00 | | 575.00 | | 2152.50 | 4172.00 | 7525.00 |
| Tukey's Hinges | | | Blood Loss/ml | | yes | |  | |  | | 50.00 | | 300.00 | | 500.00 |  |  |
|  |  |  |  |  | no | |  | |  | | 80.00 | | 575.00 | | 2100.00 |  |  |

**Blood Loss/ml**


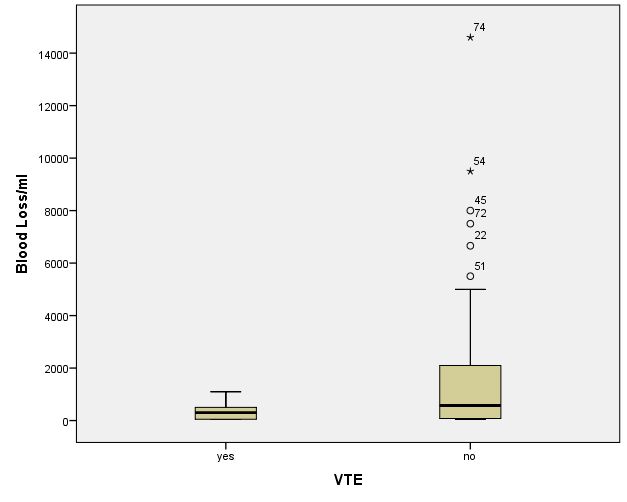


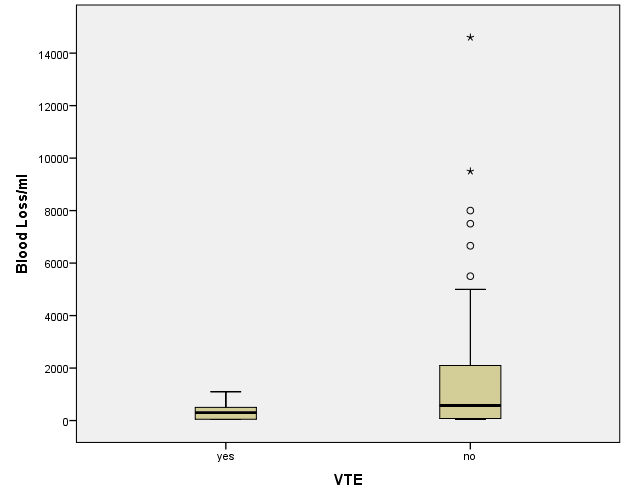


**Nonparametric Tests**


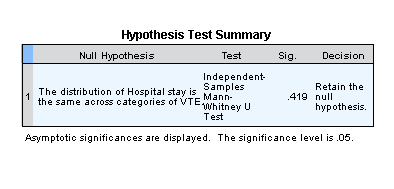


**Explore**

**VTE**

| **Case Processing Summary** | | | | | | | | | | | | | |  |  |  |  |
| --- | --- | --- | --- | --- | --- | --- | --- | --- | --- | --- | --- | --- | --- | --- | --- | --- | --- |
|  | VTE | Cases | | | | | | | | | | | |  |  |  |  |
|  |  | Valid | | | | Missing | | | | Total | | | |  |  |  |  |
|  |  | N | | Percent | | N | | Percent | | N | | Percent | |  |  |  |  |
| Hospital stay | yes | 6 | | 100.0% | | 0 | | 0.0% | | 6 | | 100.0% | |  |  |  |  |
|  | no | 79 | | 100.0% | | 0 | | 0.0% | | 79 | | 100.0% | |  |  |  |  |
| **Percentiles** | | | | | | | | | | | | | | | | | |
|  | | |  | | VTE | | Percentiles | | | | | | | | | | |
|  | | |  | |  |  | 5 | | 10 | | 25 | | 50 | | 75 | 90 | 95 |
| Weighted Average(Definition 1) | | | Hospital stay | | yes | | 5.00 | | 5.00 | | 5.75 | | 9.50 | | 19.00 | . | . |
|  |  |  |  |  | no | | 4.00 | | 5.00 | | 7.00 | | 11.00 | | 23.00 | 60.00 | 106.00 |
| Tukey's Hinges | | | Hospital stay | | yes | |  | |  | | 6.00 | | 9.50 | | 12.00 |  |  |
|  |  |  |  |  | no | |  | |  | | 7.00 | | 11.00 | | 22.50 |  |  |

**Hospital stay**


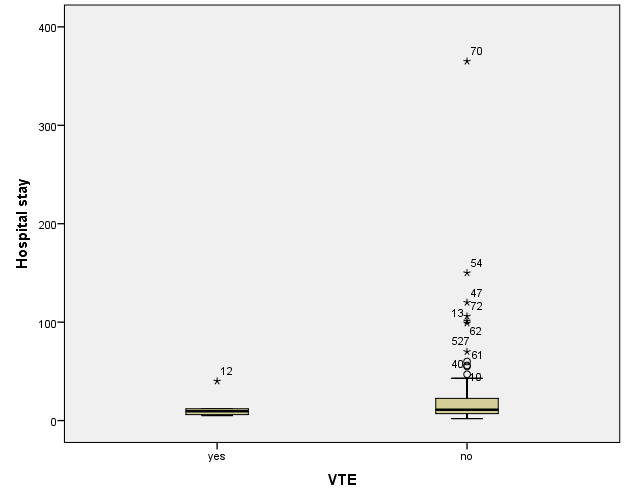


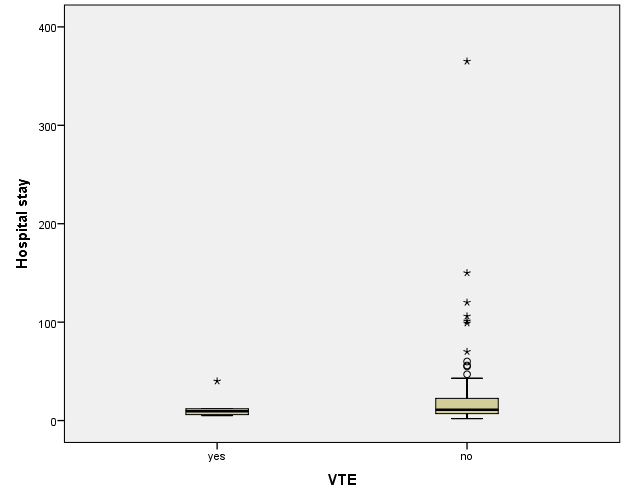


**Nonparametric Tests**


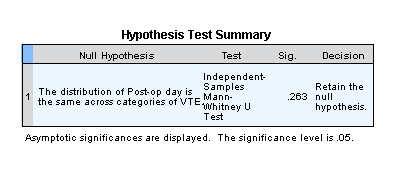


**Explore**

**VTE**

| **Case Processing Summary** | | | | | | | | | | | | | |  |  |  |  |
| --- | --- | --- | --- | --- | --- | --- | --- | --- | --- | --- | --- | --- | --- | --- | --- | --- | --- |
|  | VTE | Cases | | | | | | | | | | | |  |  |  |  |
|  |  | Valid | | | | Missing | | | | Total | | | |  |  |  |  |
|  |  | N | | Percent | | N | | Percent | | N | | Percent | |  |  |  |  |
| Post-op day | yes | 6 | | 100.0% | | 0 | | 0.0% | | 6 | | 100.0% | |  |  |  |  |
|  | no | 79 | | 100.0% | | 0 | | 0.0% | | 79 | | 100.0% | |  |  |  |  |
| **Percentiles** | | | | | | | | | | | | | | | | | |
|  | | |  | | VTE | | Percentiles | | | | | | | | | | |
|  | | |  | |  |  | 5 | | 10 | | 25 | | 50 | | 75 | 90 | 95 |
| Weighted Average(Definition 1) | | | Post-op day | | yes | | 2.000 | | 2.000 | | 4.250 | | 8.000 | | 10.250 | . | . |
|  |  |  |  |  | no | | 2.000 | | 3.000 | | 7.000 | | 11.000 | | 14.000 | 19.000 | 23.000 |
| Tukey's Hinges | | | Post-op day | | yes | |  | |  | | 5.000 | | 8.000 | | 8.000 |  |  |
|  |  |  |  |  | no | |  | |  | | 7.000 | | 11.000 | | 14.000 |  |  |

**Post-op day**


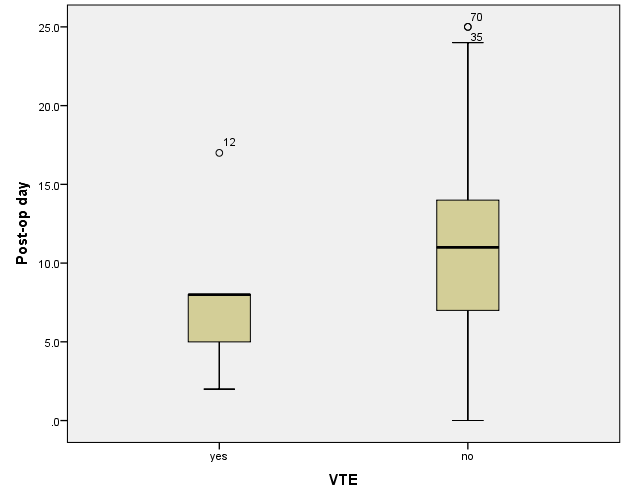


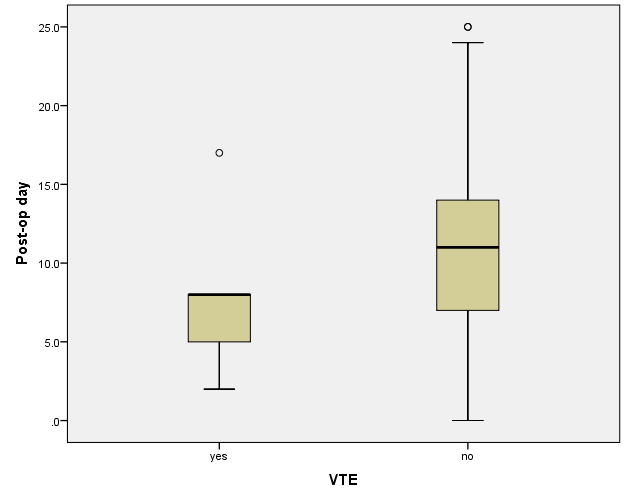


**Nonparametric Tests**


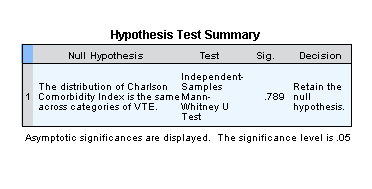


**Explore**

**VTE**

| **Case Processing Summary** | | | | | | | | | | | | |  |  |  |  |
| --- | --- | --- | --- | --- | --- | --- | --- | --- | --- | --- | --- | --- | --- | --- | --- | --- |
|  | VTE | Cases | | | | | | | | | | |  |  |  |  |
|  |  | Valid | | | Missing | | | | Total | | | |  |  |  |  |
|  |  | N | Percent | | N | | Percent | | N | | Percent | |  |  |  |  |
| Charlson Comorbidity Index | yes | 6 | 100.0% | | 0 | | 0.0% | | 6 | | 100.0% | |  |  |  |  |
|  | no | 79 | 100.0% | | 0 | | 0.0% | | 79 | | 100.0% | |  |  |  |  |
| **Percentiles** | | | | | | | | | | | | | | | | |
|  |  | | | VTE | | Percentiles | | | | | | | | | | |
|  |  | | |  |  | 5 | | 10 | | 25 | | 50 | | 75 | 90 | 95 |
| Weighted Average(Definition 1) | Charlson Comorbidity Index | | | yes | | .0000 | | .0000 | | .0000 | | 1.0000 | | 3.5000 | . | . |
|  |  |  |  | no | | .0000 | | .0000 | | .0000 | | 1.0000 | | 3.0000 | 5.0000 | 7.0000 |
| Tukey's Hinges | Charlson Comorbidity Index | | | yes | |  | |  | | .0000 | | 1.0000 | | 3.0000 |  |  |
|  |  |  |  | no | |  | |  | | .0000 | | 1.0000 | | 3.0000 |  |  |

**Charlson Comorbidity Index**


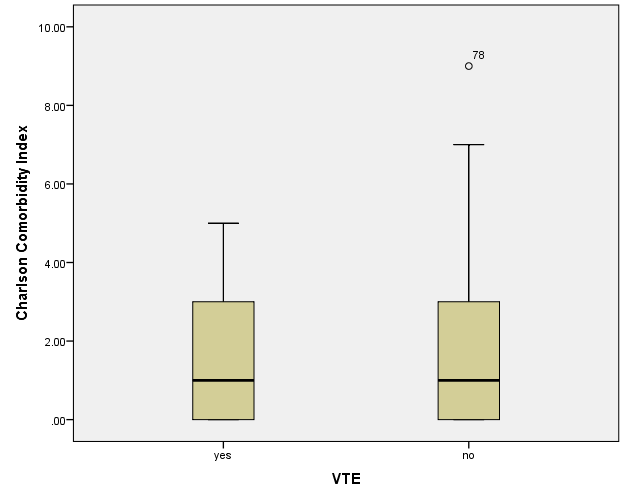


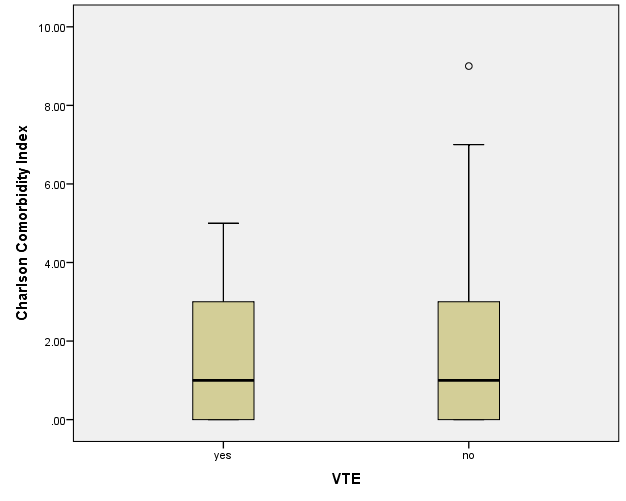


**Logistic Regression**

| **Case Processing Summary** | | | |
| --- | --- | --- | --- |
| Unweighted Cases^a^ | | N | Percent |
| Selected Cases | Included in Analysis | 84 | 98.8 |
|  | Missing Cases | 1 | 1.2 |
|  | Total | 85 | 100.0 |
| Unselected Cases | | 0 | .0 |
| Total | | 85 | 100.0 |
| a. If weight is in effect, see classification table for the total number of cases. | | | |

| **Dependent Variable Encoding** | |
| --- | --- |
| Original Value | Internal Value |
| yes | 0 |
| no | 1 |

| **Categorical Variables Codings** | | | | | | |
| --- | --- | --- | --- | --- | --- | --- |
|  | | Frequency | Parameter coding | | | |
|  |  |  | (1) | (2) | (3) | (4) |
| Age | <18 | 2 | .000 | .000 | .000 | .000 |
|  | 18-29 | 23 | 1.000 | .000 | .000 | .000 |
|  | 30-49 | 21 | .000 | 1.000 | .000 | .000 |
|  | 50-65 | 22 | .000 | .000 | 1.000 | .000 |
|  | >65 | 16 | .000 | .000 | .000 | 1.000 |
| OCP | yes | 3 | .000 | .000 |  |  |
|  | no | 38 | 1.000 | .000 |  |  |
|  | N/A | 43 | .000 | 1.000 |  |  |
| BMI | <30 | 24 | .000 | .000 |  |  |
|  | >=30 | 9 | 1.000 | .000 |  |  |
|  | not mentioned | 51 | .000 | 1.000 |  |  |
| Anticoagulation | yes | 15 | .000 |  |  |  |
|  | no | 69 | 1.000 |  |  |  |
| Type | Emergency | 30 | .000 |  |  |  |
|  | Elective | 54 | 1.000 |  |  |  |
| Gender | male | 44 | .000 |  |  |  |
|  | female | 40 | 1.000 |  |  |  |

**Block 0: Beginning Block**

| **Classification Table^a,b^** | | | | | |
| --- | --- | --- | --- | --- | --- |
|  | Observed | | Predicted | | |
|  |  |  | VTE | | Percentage Correct |
|  |  |  | yes | no |  |
| Step 0 | VTE | yes | 0 | 6 | .0 |
|  |  | no | 0 | 78 | 100.0 |
|  | Overall Percentage | |  |  | 92.9 |
| a. Constant is included in the model. | | | | | |
| b. The cut value is .500 | | | | | |

| **Variables in the Equation** | | | | | | | | | | | | |
| --- | --- | --- | --- | --- | --- | --- | --- | --- | --- | --- | --- | --- |
|  | | B | | S.E. | | Wald | | df | | Sig. | | Exp(B) |
| Step 0 | Constant | 2.565 | | .424 | | 36.654 | | 1 | | .000 | | 13.000 |
| **Variables not in the Equation** | | | | | | | | | | |  |  |
|  | | | | | Score | | df | | Sig. | |  |  |
| Step 0 | Variables | | Age | | 1.520 | | 4 | | .823 | |  |  |
|  |  |  | Age(1) | | .373 | | 1 | | .541 | |  |  |
|  |  |  | Age(2) | | .239 | | 1 | | .625 | |  |  |
|  |  |  | Age(3) | | .303 | | 1 | | .582 | |  |  |
|  |  |  | Age(4) | | .855 | | 1 | | .355 | |  |  |
|  |  |  | Gender(1) | | 3.304 | | 1 | | .069 | |  |  |
|  |  |  | BMI | | 11.424 | | 2 | | .003 | |  |  |
|  |  |  | BMI(1) | | 10.425 | | 1 | | .001 | |  |  |
|  |  |  | BMI(2) | | 5.256 | | 1 | | .022 | |  |  |
|  | Overall Statistics | | | | 17.081 | | 7 | | .017 | |  |  |

**Block 1: Method = Enter**

| **Omnibus Tests of Model Coefficients** | | | | | | | | | | | | | | | | | | |  |  |  |  |
| --- | --- | --- | --- | --- | --- | --- | --- | --- | --- | --- | --- | --- | --- | --- | --- | --- | --- | --- | --- | --- | --- | --- |
|  | | | | Chi-square | | | | | | df | | | | | Sig. | | | |  |  |  |  |
| Step 1 | | Step | | 13.960 | | | | | | 7 | | | | | .052 | | | |  |  |  |  |
|  |  | Block | | 13.960 | | | | | | 7 | | | | | .052 | | | |  |  |  |  |
|  |  | Model | | 13.960 | | | | | | 7 | | | | | .052 | | | |  |  |  |  |
| **Model Summary** | | | | | | | | | | | | | | | | | | | |  |  |  |
| Step | -2 Log likelihood | | | | | | Cox & Snell R Square | | | | | Nagelkerke R Square | | | | | | | |  |  |  |
| 1 | 29.270^a^ | | | | | | .153 | | | | | .381 | | | | | | | |  |  |  |
| a. Estimation terminated at iteration number 20 because maximum iterations has been reached. Final solution cannot be found. | | | | | | | | | | | | | | | | | | | |  |  |  |
| **Hosmer and Lemeshow Test** | | | | | | | | | | | | | | | |  |  |  |  |  |  |  |
| Step | Chi-square | | | | | df | | | | | Sig. | | | | |  |  |  |  |  |  |  |
| 1 | 6.278 | | | | | 8 | | | | | .616 | | | | |  |  |  |  |  |  |  |
| **Contingency Table for Hosmer and Lemeshow Test** | | | | | | | | | | | | | | | | | | | | | | |
|  | | | VTE = yes | | | | | | | | | | VTE = no | | | | | | | | Total | |
|  |  |  | Observed | | | | | Expected | | | | | Observed | | | | | Expected | | |  |  |
| Step 1 | | 1 | 3 | | | | | 3.281 | | | | | 5 | | | | | 4.719 | | | 8 | |
|  |  | 2 | 1 | | | | | 1.135 | | | | | 7 | | | | | 6.865 | | | 8 | |
|  |  | 3 | 0 | | | | | .469 | | | | | 9 | | | | | 8.531 | | | 9 | |
|  |  | 4 | 0 | | | | | .284 | | | | | 6 | | | | | 5.716 | | | 6 | |
|  |  | 5 | 1 | | | | | .340 | | | | | 7 | | | | | 7.660 | | | 8 | |
|  |  | 6 | 1 | | | | | .182 | | | | | 7 | | | | | 7.818 | | | 8 | |
|  |  | 7 | 0 | | | | | .056 | | | | | 4 | | | | | 3.944 | | | 4 | |
|  |  | 8 | 0 | | | | | .151 | | | | | 12 | | | | | 11.849 | | | 12 | |
|  |  | 9 | 0 | | | | | .066 | | | | | 8 | | | | | 7.934 | | | 8 | |
|  |  | 10 | 0 | | | | | .036 | | | | | 13 | | | | | 12.964 | | | 13 | |
| **Classification Table^a^** | | | | | | | | | | | | | | | | | | | | | |  |
|  | | Observed | | | | | | | Predicted | | | | | | | | | | | | |  |
|  | |  |  |  |  |  |  |  | VTE | | | | | | | | Percentage Correct | | | | |  |
|  | |  |  |  |  |  |  |  | yes | | | | | no | | |  |  |  |  |  |  |
| Step 1 | | VTE | | | yes | | | | 3 | | | | | 3 | | | 50.0 | | | | |  |
|  |  |  |  |  | no | | | | 0 | | | | | 78 | | | 100.0 | | | | |  |
|  |  | Overall Percentage | | | | | | |  | | | | |  | | | 96.4 | | | | |  |
| a. The cut value is .500 | | | | | | | | | | | | | | | | | | | | | |  |

| **Variables in the Equation** | | | | | | | | | |
| --- | --- | --- | --- | --- | --- | --- | --- | --- | --- |
|  | | B | S.E. | Wald | df | Sig. | Exp(B) | 95% C.I.for EXP(B) | |
|  |  |  |  |  |  |  |  | Lower | Upper |
| Step 1^a^ | Age |  |  | 2.768 | 4 | .597 |  |  |  |
|  | Age(1) | -16.840 | 28420.712 | .000 | 1 | 1.000 | .000 | .000 | . |
|  | Age(2) | -16.410 | 28420.712 | .000 | 1 | 1.000 | .000 | .000 | . |
|  | Age(3) | -14.201 | 28420.712 | .000 | 1 | 1.000 | .000 | .000 | . |
|  | Age(4) | -16.954 | 28420.712 | .000 | 1 | 1.000 | .000 | .000 | . |
|  | Gender(1) | -1.248 | 1.270 | .965 | 1 | .326 | .287 | .024 | 3.460 |
|  | BMI |  |  | 5.754 | 2 | .056 |  |  |  |
|  | BMI(1) | -2.442 | 1.515 | 2.600 | 1 | .107 | .087 | .004 | 1.693 |
|  | BMI(2) | 1.835 | 1.324 | 1.923 | 1 | .166 | 6.268 | .468 | 83.894 |
|  | Constant | 19.367 | 28420.712 | .000 | 1 | .999 | 257735197.559 |  |  |
| a. Variable(s) entered on step 1: Age, Gender, BMI. | | | | | | | | | |
