# Supplementary material for: Evaluation of the risk factors for venous thromboembolism post splenectomy – A ten year retrospective cohort study in St James’s hospital
Source: Ann Med Surg (Lond). 2021 May 8;66:102381. doi: 10.1016/j.amsu.2021.102381 (PMC8131975; doi:10.1016/j.amsu.2021.102381)

**Crosstabs females only**

| **Case Processing Summary** | | | | | | |
| --- | --- | --- | --- | --- | --- | --- |
|  | Cases | | | | | |
|  | Valid | | Missing | | Total | |
|  | N | Percent | N | Percent | N | Percent |
| VTE * OCP | 41 | 100.0% | 0 | 0.0% | 41 | 100.0% |

| **VTE * OCP Crosstabulation** | | | | | | |
| --- | --- | --- | --- | --- | --- | --- |
|  | | | OCP | | | Total |
|  |  |  | yes | no | N/A |  |
| VTE | yes | Count | 0 | 5 | 0 | 5 |
|  |  | Expected Count | .4 | 4.5 | .1 | 5.0 |
|  |  | % within OCP | 0.0% | 13.5% | 0.0% | 12.2% |
|  | no | Count | 3 | 32 | 1 | 36 |
|  |  | Expected Count | 2.6 | 32.5 | .9 | 36.0 |
|  |  | % within OCP | 100.0% | 86.5% | 100.0% | 87.8% |
| Total | | Count | 3 | 37 | 1 | 41 |
|  |  | Expected Count | 3.0 | 37.0 | 1.0 | 41.0 |
|  |  | % within OCP | 100.0% | 100.0% | 100.0% | 100.0% |

| **Chi-Square Tests** | | | | | | | | | | | |
| --- | --- | --- | --- | --- | --- | --- | --- | --- | --- | --- | --- |
|  | | Value | | df | | Asymp. Sig. (2-sided) | | Exact Sig. (2-sided) | | Exact Sig. (1-sided) | Point Probability |
| Pearson Chi-Square | | .616^a^ | | 2 | | .735 | | 1.000 | |  |  |
| Likelihood Ratio | | 1.099 | | 2 | | .577 | | 1.000 | |  |  |
| Fisher's Exact Test | | .880 | |  | |  | | 1.000 | |  |  |
| Linear-by-Linear Association | | .139^b^ | | 1 | | .709 | | 1.000 | | .701 | .613 |
| N of Valid Cases | | 41 | |  | |  | |  | |  |  |
| a. 5 cells (83.3%) have expected count less than 5. The minimum expected count is .12. | | | | | | | | | | | |
| b. The standardized statistic is -.373. | | | | | | | | | | | |
| **Symmetric Measures** | | | | | | | | |  |  |  |
|  | | | Value | | Approx. Sig. | | Exact Sig. | |  |  |  |
| Nominal by Nominal | Phi | | .123 | | .735 | | 1.000 | |  |  |  |
|  | Cramer's V | | .123 | | .735 | | 1.000 | |  |  |  |
| N of Valid Cases | | | 41 | |  | |  | |  |  |  |
| **Risk Estimate** | | | |  |  |  |  |  |  |  |  |
|  | | Value | |  |  |  |  |  |  |  |  |
| Odds Ratio for VTE (yes / no) | | ^a^ | |  |  |  |  |  |  |  |  |
| a. Risk Estimate statistics cannot be computed. They are only computed for a 2*2 table without empty cells. | | | |  |  |  |  |  |  |  |  |


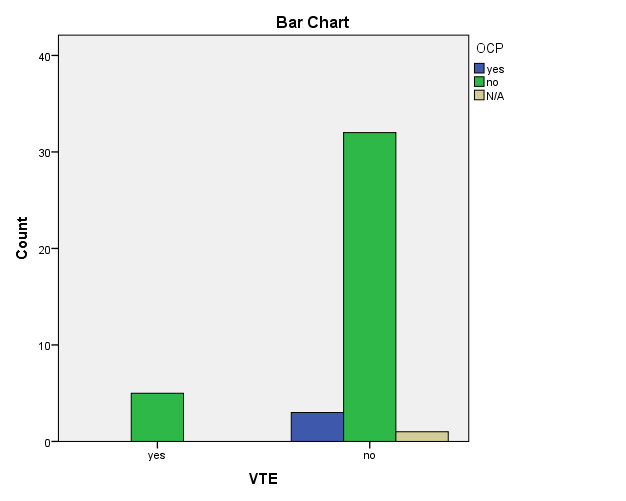


**Crosstabs**

| **Case Processing Summary** | | | | | | |
| --- | --- | --- | --- | --- | --- | --- |
|  | Cases | | | | | |
|  | Valid | | Missing | | Total | |
|  | N | Percent | N | Percent | N | Percent |
| Gender * BMI | 85 | 100.0% | 0 | 0.0% | 85 | 100.0% |

| **Gender * BMI Crosstabulation** | | | | | | |
| --- | --- | --- | --- | --- | --- | --- |
|  | | | BMI | | | Total |
|  |  |  | <30 | >=30 | not mentioned |  |
| Gender | male | Count | 13 | 2 | 29 | 44 |
|  |  | Expected Count | 12.9 | 4.7 | 26.4 | 44.0 |
|  |  | % within BMI | 52.0% | 22.2% | 56.9% | 51.8% |
|  | female | Count | 12 | 7 | 22 | 41 |
|  |  | Expected Count | 12.1 | 4.3 | 24.6 | 41.0 |
|  |  | % within BMI | 48.0% | 77.8% | 43.1% | 48.2% |
| Total | | Count | 25 | 9 | 51 | 85 |
|  |  | Expected Count | 25.0 | 9.0 | 51.0 | 85.0 |
|  |  | % within BMI | 100.0% | 100.0% | 100.0% | 100.0% |

| **Chi-Square Tests** | | | | | | |
| --- | --- | --- | --- | --- | --- | --- |
|  | Value | df | Asymp. Sig. (2-sided) | Exact Sig. (2-sided) | Exact Sig. (1-sided) | Point Probability |
| Pearson Chi-Square | 3.677^a^ | 2 | .159 | .180 |  |  |
| Likelihood Ratio | 3.840 | 2 | .147 | .171 |  |  |
| Fisher's Exact Test | 3.566 |  |  | .180 |  |  |
| Linear-by-Linear Association | .376^b^ | 1 | .540 | .550 | .311 | .080 |
| N of Valid Cases | 85 |  |  |  |  |  |
| a. 2 cells (33.3%) have expected count less than 5. The minimum expected count is 4.34. | | | | | | |
| b. The standardized statistic is -.613. | | | | | | |

| **Symmetric Measures** | | | | |
| --- | --- | --- | --- | --- |
|  | | Value | Approx. Sig. | Exact Sig. |
| Nominal by Nominal | Phi | .208 | .159 | .180 |
|  | Cramer's V | .208 | .159 | .180 |
| N of Valid Cases | | 85 |  |  |

| **Risk Estimate** | |
| --- | --- |
|  | Value |
| Odds Ratio for Gender (male / female) | ^a^ |
| a. Risk Estimate statistics cannot be computed. They are only computed for a 2*2 table without empty cells. | |


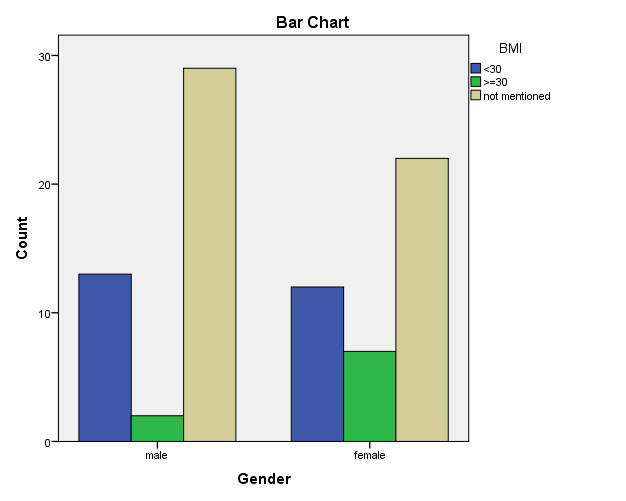


**Crosstabs**

| **Case Processing Summary** | | | | | | |
| --- | --- | --- | --- | --- | --- | --- |
|  | Cases | | | | | |
|  | Valid | | Missing | | Total | |
|  | N | Percent | N | Percent | N | Percent |
| VTE * BMI | 34 | 100.0% | 0 | 0.0% | 34 | 100.0% |

| **VTE * BMI Crosstabulation** | | | | | |
| --- | --- | --- | --- | --- | --- |
|  | | | BMI | | Total |
|  |  |  | <30 | >=30 |  |
| VTE | yes | Count | 2 | 3 | 5 |
|  |  | Expected Count | 3.7 | 1.3 | 5.0 |
|  |  | % within BMI | 8.0% | 33.3% | 14.7% |
|  | no | Count | 23 | 6 | 29 |
|  |  | Expected Count | 21.3 | 7.7 | 29.0 |
|  |  | % within BMI | 92.0% | 66.7% | 85.3% |
| Total | | Count | 25 | 9 | 34 |
|  |  | Expected Count | 25.0 | 9.0 | 34.0 |
|  |  | % within BMI | 100.0% | 100.0% | 100.0% |

| **Chi-Square Tests** | | | | | | | | | | | | | |
| --- | --- | --- | --- | --- | --- | --- | --- | --- | --- | --- | --- | --- | --- |
|  | | Value | | df | | Asymp. Sig. (2-sided) | | | | Exact Sig. (2-sided) | | Exact Sig. (1-sided) | Point Probability |
| Pearson Chi-Square | | 3.386^a^ | | 1 | | .066 | | | | .102 | | .102 |  |
| Continuity Correction^b^ | | 1.667 | | 1 | | .197 | | | |  | |  |  |
| Likelihood Ratio | | 2.999 | | 1 | | .083 | | | | .293 | | .102 |  |
| Fisher's Exact Test | |  | |  | |  | | | | .102 | | .102 |  |
| Linear-by-Linear Association | | 3.286^c^ | | 1 | | .070 | | | | .102 | | .102 | .091 |
| N of Valid Cases | | 34 | |  | |  | | | |  | |  |  |
| a. 2 cells (50.0%) have expected count less than 5. The minimum expected count is 1.32. | | | | | | | | | | | | | |
| b. Computed only for a 2x2 table | | | | | | | | | | | | | |
| c. The standardized statistic is -1.813. | | | | | | | | | | | | | |
| **Symmetric Measures** | | | | | | | | | | |  |  |  |
|  | | | Value | | Approx. Sig. | | | Exact Sig. | | |  |  |  |
| Nominal by Nominal | Phi | | -.316 | | .066 | | | .102 | | |  |  |  |
|  | Cramer's V | | .316 | | .066 | | | .102 | | |  |  |  |
| N of Valid Cases | | | 34 | |  | | |  | | |  |  |  |
| **Risk Estimate** | | | | | | | | |  |  |  |  |  |
|  | | Value | | 95% Confidence Interval | | | | |  |  |  |  |  |
|  |  |  |  | Lower | | | Upper | |  |  |  |  |  |
| Odds Ratio for VTE (yes / no) | | .174 | | .023 | | | 1.288 | |  |  |  |  |  |
| For cohort BMI = <30 | | .504 | | .170 | | | 1.499 | |  |  |  |  |  |
| For cohort BMI = >=30 | | 2.900 | | 1.056 | | | 7.962 | |  |  |  |  |  |
| N of Valid Cases | | 34 | |  | | |  | |  |  |  |  |  |


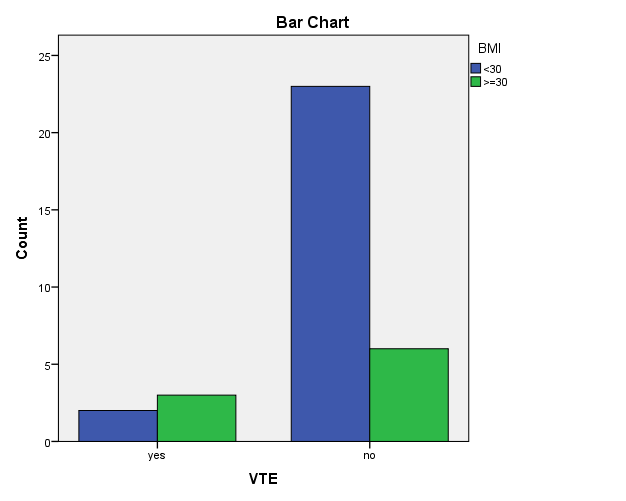


**Frequencies**

| **Statistics** | | |
| --- | --- | --- |
| Age | | |
| N | Valid | 85 |
|  | Missing | 0 |

| **Age** | | | | | |
| --- | --- | --- | --- | --- | --- |
|  | | Frequency | Percent | Valid Percent | Cumulative Percent |
| Valid | <18 | 2 | 2.4 | 2.4 | 2.4 |
|  | 18-29 | 23 | 27.1 | 27.1 | 29.4 |
|  | 30-49 | 21 | 24.7 | 24.7 | 54.1 |
|  | 50-65 | 23 | 27.1 | 27.1 | 81.2 |
|  | >65 | 16 | 18.8 | 18.8 | 100.0 |
|  | Total | 85 | 100.0 | 100.0 |  |


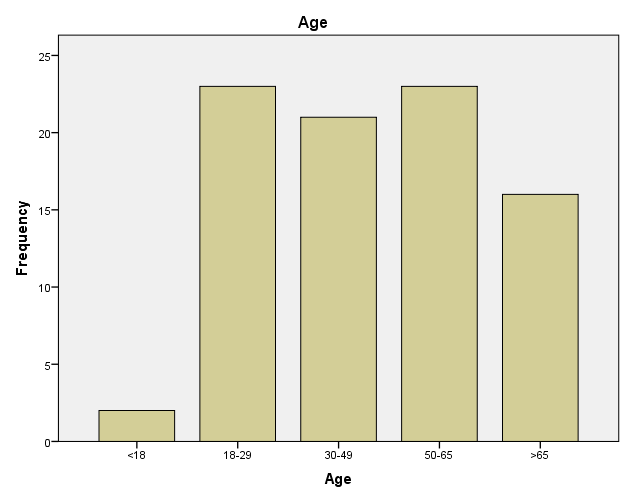


**Crosstabs**

| **Case Processing Summary** | | | | | | |
| --- | --- | --- | --- | --- | --- | --- |
|  | Cases | | | | | |
|  | Valid | | Missing | | Total | |
|  | N | Percent | N | Percent | N | Percent |
| VTE * merged ages | 85 | 100.0% | 0 | 0.0% | 85 | 100.0% |

| **VTE * merged ages Crosstabulation** | | | | | |
| --- | --- | --- | --- | --- | --- |
|  | | | merged ages | | Total |
|  |  |  | <50 | >=50 |  |
| VTE | yes | Count | 3 | 3 | 6 |
|  |  | Expected Count | 3.2 | 2.8 | 6.0 |
|  |  | % within merged ages | 6.5% | 7.7% | 7.1% |
|  | no | Count | 43 | 36 | 79 |
|  |  | Expected Count | 42.8 | 36.2 | 79.0 |
|  |  | % within merged ages | 93.5% | 92.3% | 92.9% |
| Total | | Count | 46 | 39 | 85 |
|  |  | Expected Count | 46.0 | 39.0 | 85.0 |
|  |  | % within merged ages | 100.0% | 100.0% | 100.0% |

| **Chi-Square Tests** | | | | | | |
| --- | --- | --- | --- | --- | --- | --- |
|  | Value | df | Asymp. Sig. (2-sided) | Exact Sig. (2-sided) | Exact Sig. (1-sided) | Point Probability |
| Pearson Chi-Square | .044^a^ | 1 | .834 | 1.000 | .580 |  |
| Continuity Correction^b^ | .000 | 1 | 1.000 |  |  |  |
| Likelihood Ratio | .044 | 1 | .834 | 1.000 | .580 |  |
| Fisher's Exact Test |  |  |  | 1.000 | .580 |  |
| Linear-by-Linear Association | .044^c^ | 1 | .835 | 1.000 | .580 | .317 |
| N of Valid Cases | 85 |  |  |  |  |  |
| a. 2 cells (50.0%) have expected count less than 5. The minimum expected count is 2.75. | | | | | | |
| b. Computed only for a 2x2 table | | | | | | |
| c. The standardized statistic is -.209. | | | | | | |

| **Symmetric Measures** | | | | |
| --- | --- | --- | --- | --- |
|  | | Value | Approx. Sig. | Exact Sig. |
| Nominal by Nominal | Phi | -.023 | .834 | 1.000 |
|  | Cramer's V | .023 | .834 | 1.000 |
| N of Valid Cases | | 85 |  |  |

| **Risk Estimate** | | | |
| --- | --- | --- | --- |
|  | Value | 95% Confidence Interval | |
|  |  | Lower | Upper |
| Odds Ratio for VTE (yes / no) | .837 | .159 | 4.405 |
| For cohort merged ages = <50 | .919 | .402 | 2.097 |
| For cohort merged ages = >=50 | 1.097 | .476 | 2.531 |
| N of Valid Cases | 85 |  |  |


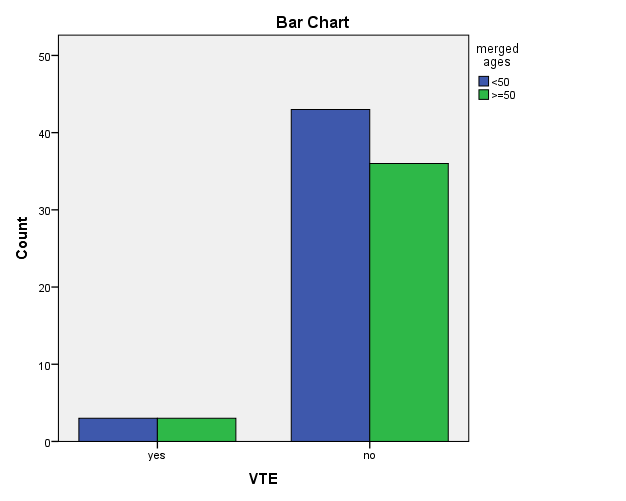


**Frequencies**

| **Statistics** | | |
| --- | --- | --- |
| Pre-operative platelets | | |
| N | Valid | 85 |
|  | Missing | 0 |

| **Pre-operative platelets** | | | | | |
| --- | --- | --- | --- | --- | --- |
|  | | Frequency | Percent | Valid Percent | Cumulative Percent |
| Valid | 50-99 | 11 | 12.9 | 12.9 | 12.9 |
|  | 100-450 | 68 | 80.0 | 80.0 | 92.9 |
|  | >450 | 6 | 7.1 | 7.1 | 100.0 |
|  | Total | 85 | 100.0 | 100.0 |  |


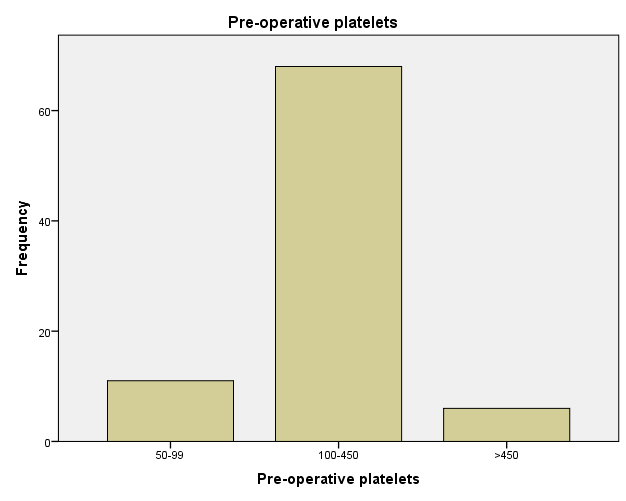


**Crosstabs**

| **Case Processing Summary** | | | | | | |
| --- | --- | --- | --- | --- | --- | --- |
|  | Cases | | | | | |
|  | Valid | | Missing | | Total | |
|  | N | Percent | N | Percent | N | Percent |
| VTE * preop plt changed categories | 85 | 100.0% | 0 | 0.0% | 85 | 100.0% |

| **VTE * preop plt changed categories Crosstabulation** | | | | | |
| --- | --- | --- | --- | --- | --- |
|  | | | preop plt changed categories | | Total |
|  |  |  | 50-99 | >100 |  |
| VTE | yes | Count | 1 | 5 | 6 |
|  |  | Expected Count | .8 | 5.2 | 6.0 |
|  |  | % within preop plt changed categories | 9.1% | 6.8% | 7.1% |
|  | no | Count | 10 | 69 | 79 |
|  |  | Expected Count | 10.2 | 68.8 | 79.0 |
|  |  | % within preop plt changed categories | 90.9% | 93.2% | 92.9% |
| Total | | Count | 11 | 74 | 85 |
|  |  | Expected Count | 11.0 | 74.0 | 85.0 |
|  |  | % within preop plt changed categories | 100.0% | 100.0% | 100.0% |

| **Chi-Square Tests** | | | | | | | | | | | | | |
| --- | --- | --- | --- | --- | --- | --- | --- | --- | --- | --- | --- | --- | --- |
|  | | Value | | df | | Asymp. Sig. (2-sided) | | | | Exact Sig. (2-sided) | | Exact Sig. (1-sided) | Point Probability |
| Pearson Chi-Square | | .080^a^ | | 1 | | .778 | | | | 1.000 | | .576 |  |
| Continuity Correction^b^ | | .000 | | 1 | | 1.000 | | | |  | |  |  |
| Likelihood Ratio | | .074 | | 1 | | .785 | | | | 1.000 | | .576 |  |
| Fisher's Exact Test | |  | |  | |  | | | | .576 | | .576 |  |
| Linear-by-Linear Association | | .079^c^ | | 1 | | .779 | | | | 1.000 | | .576 | .405 |
| N of Valid Cases | | 85 | |  | |  | | | |  | |  |  |
| a. 1 cells (25.0%) have expected count less than 5. The minimum expected count is .78. | | | | | | | | | | | | | |
| b. Computed only for a 2x2 table | | | | | | | | | | | | | |
| c. The standardized statistic is .280. | | | | | | | | | | | | | |
| **Symmetric Measures** | | | | | | | | | | |  |  |  |
|  | | | Value | | Approx. Sig. | | | Exact Sig. | | |  |  |  |
| Nominal by Nominal | Phi | | .031 | | .778 | | | 1.000 | | |  |  |  |
|  | Cramer's V | | .031 | | .778 | | | 1.000 | | |  |  |  |
| N of Valid Cases | | | 85 | |  | | |  | | |  |  |  |
| **Risk Estimate** | | | | | | | | |  |  |  |  |  |
|  | | Value | | 95% Confidence Interval | | | | |  |  |  |  |  |
|  |  |  |  | Lower | | | Upper | |  |  |  |  |  |
| Odds Ratio for VTE (yes / no) | | 1.380 | | .146 | | | 13.055 | |  |  |  |  |  |
| For cohort preop plt changed categories = 50-99 | | 1.317 | | .201 | | | 8.634 | |  |  |  |  |  |
| For cohort preop plt changed categories = >100 | | .954 | | .661 | | | 1.378 | |  |  |  |  |  |
| N of Valid Cases | | 85 | |  | | |  | |  |  |  |  |  |


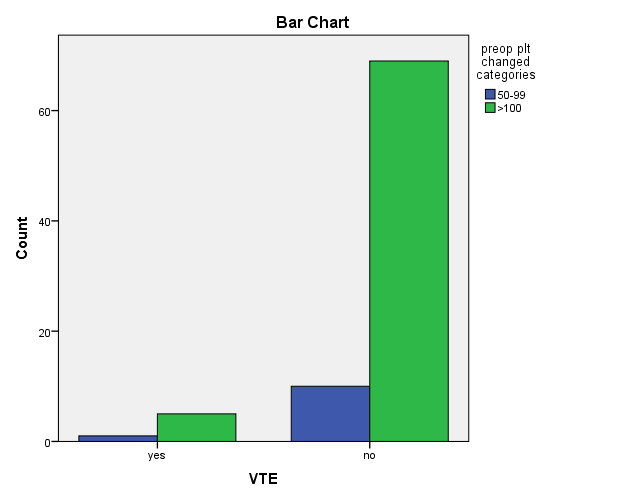


**Crosstabs**

| **Case Processing Summary** | | | | | | | | | | | | |
| --- | --- | --- | --- | --- | --- | --- | --- | --- | --- | --- | --- | --- |
|  | | | Cases | | | | | | | | | |
|  |  |  | Valid | | | Missing | | | Total | | | |
|  |  |  | N | Percent | | N | | Percent | N | | Percent | |
| VTE * preop plt changed categories | | | 85 | 100.0% | | 0 | | 0.0% | 85 | | 100.0% | |
| **VTE * preop plt changed categories Crosstabulation** | | | | | | | | | | | |  |
|  | | | | | preop plt changed categories | | | | | Total | |  |
|  |  |  |  |  | <450 | | >450 | | |  |  |  |
| VTE | yes | Count | | | 5 | | 1 | | | 6 | |  |
|  |  | Expected Count | | | 5.6 | | .4 | | | 6.0 | |  |
|  |  | % within preop plt changed categories | | | 6.3% | | 16.7% | | | 7.1% | |  |
|  | no | Count | | | 74 | | 5 | | | 79 | |  |
|  |  | Expected Count | | | 73.4 | | 5.6 | | | 79.0 | |  |
|  |  | % within preop plt changed categories | | | 93.7% | | 83.3% | | | 92.9% | |  |
| Total | | Count | | | 79 | | 6 | | | 85 | |  |
|  |  | Expected Count | | | 79.0 | | 6.0 | | | 85.0 | |  |
|  |  | % within preop plt changed categories | | | 100.0% | | 100.0% | | | 100.0% | |  |

| **Chi-Square Tests** | | | | | | |
| --- | --- | --- | --- | --- | --- | --- |
|  | Value | df | Asymp. Sig. (2-sided) | Exact Sig. (2-sided) | Exact Sig. (1-sided) | Point Probability |
| Pearson Chi-Square | .908^a^ | 1 | .341 | .364 | .364 |  |
| Continuity Correction^b^ | .016 | 1 | .899 |  |  |  |
| Likelihood Ratio | .693 | 1 | .405 | 1.000 | .364 |  |
| Fisher's Exact Test |  |  |  | .364 | .364 |  |
| Linear-by-Linear Association | .898^c^ | 1 | .343 | .364 | .364 | .309 |
| N of Valid Cases | 85 |  |  |  |  |  |
| a. 1 cells (25.0%) have expected count less than 5. The minimum expected count is .42. | | | | | | |
| b. Computed only for a 2x2 table | | | | | | |
| c. The standardized statistic is -.947. | | | | | | |

| **Symmetric Measures** | | | | |
| --- | --- | --- | --- | --- |
|  | | Value | Approx. Sig. | Exact Sig. |
| Nominal by Nominal | Phi | -.103 | .341 | .364 |
|  | Cramer's V | .103 | .341 | .364 |
| N of Valid Cases | | 85 |  |  |

| **Risk Estimate** | | | |
| --- | --- | --- | --- |
|  | Value | 95% Confidence Interval | |
|  |  | Lower | Upper |
| Odds Ratio for VTE (yes / no) | .338 | .033 | 3.473 |
| For cohort preop plt changed categories = <450 | .890 | .619 | 1.278 |
| For cohort preop plt changed categories = >450 | 2.633 | .364 | 19.075 |
| N of Valid Cases | 85 |  |  |


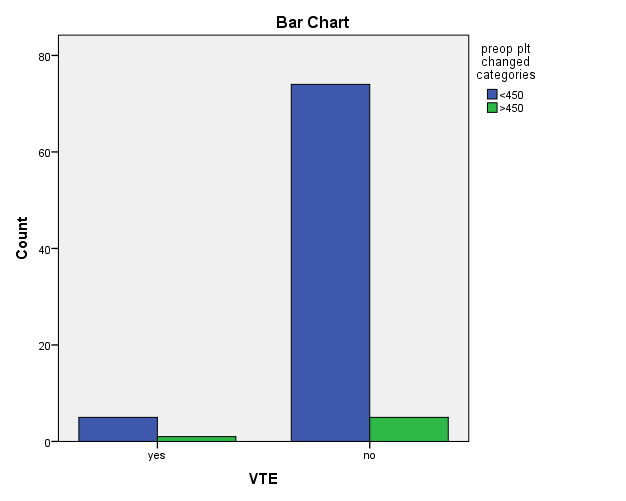


**Frequencies**

| **Statistics** | | |
| --- | --- | --- |
| Spleen size | | |
| N | Valid | 85 |
|  | Missing | 0 |

| **Spleen size** | | | | | |
| --- | --- | --- | --- | --- | --- |
|  | | Frequency | Percent | Valid Percent | Cumulative Percent |
| Valid | Normal | 64 | 75.3 | 75.3 | 75.3 |
|  | Splenomegaly | 14 | 16.5 | 16.5 | 91.8 |
|  | Massive splenomegaly | 7 | 8.2 | 8.2 | 100.0 |
|  | Total | 85 | 100.0 | 100.0 |  |


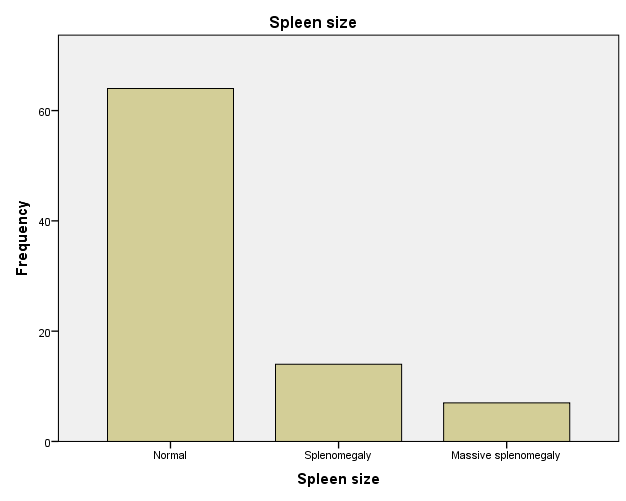


**Crosstabs**

| **Case Processing Summary** | | | | | | |
| --- | --- | --- | --- | --- | --- | --- |
|  | Cases | | | | | |
|  | Valid | | Missing | | Total | |
|  | N | Percent | N | Percent | N | Percent |
| VTE * spleen size 2 categories | 85 | 100.0% | 0 | 0.0% | 85 | 100.0% |

| **VTE * spleen size 2 categories Crosstabulation** | | | | | |
| --- | --- | --- | --- | --- | --- |
|  | | | spleen size 2 categories | | Total |
|  |  |  | Normal | Splenomegaly |  |
| VTE | yes | Count | 3 | 3 | 6 |
|  |  | Expected Count | 4.5 | 1.5 | 6.0 |
|  |  | % within spleen size 2 categories | 4.7% | 14.3% | 7.1% |
|  | no | Count | 61 | 18 | 79 |
|  |  | Expected Count | 59.5 | 19.5 | 79.0 |
|  |  | % within spleen size 2 categories | 95.3% | 85.7% | 92.9% |
| Total | | Count | 64 | 21 | 85 |
|  |  | Expected Count | 64.0 | 21.0 | 85.0 |
|  |  | % within spleen size 2 categories | 100.0% | 100.0% | 100.0% |

| **Chi-Square Tests** | | | | | | |
| --- | --- | --- | --- | --- | --- | --- |
|  | Value | df | Asymp. Sig. (2-sided) | Exact Sig. (2-sided) | Exact Sig. (1-sided) | Point Probability |
| Pearson Chi-Square | 2.220^a^ | 1 | .136 | .157 | .157 |  |
| Continuity Correction^b^ | .998 | 1 | .318 |  |  |  |
| Likelihood Ratio | 1.933 | 1 | .164 | .329 | .157 |  |
| Fisher's Exact Test |  |  |  | .157 | .157 |  |
| Linear-by-Linear Association | 2.194^c^ | 1 | .139 | .157 | .157 | .127 |
| N of Valid Cases | 85 |  |  |  |  |  |
| a. 2 cells (50.0%) have expected count less than 5. The minimum expected count is 1.48. | | | | | | |
| b. Computed only for a 2x2 table | | | | | | |
| c. The standardized statistic is -1.481. | | | | | | |

| **Symmetric Measures** | | | | |
| --- | --- | --- | --- | --- |
|  | | Value | Approx. Sig. | Exact Sig. |
| Nominal by Nominal | Phi | -.162 | .136 | .157 |
|  | Cramer's V | .162 | .136 | .157 |
| N of Valid Cases | | 85 |  |  |

| **Risk Estimate** | | | |
| --- | --- | --- | --- |
|  | Value | 95% Confidence Interval | |
|  |  | Lower | Upper |
| Odds Ratio for VTE (yes / no) | .295 | .055 | 1.590 |
| For cohort spleen size 2 categories = Normal | .648 | .288 | 1.454 |
| For cohort spleen size 2 categories = Splenomegaly | 2.194 | .895 | 5.383 |
| N of Valid Cases | 85 |  |  |


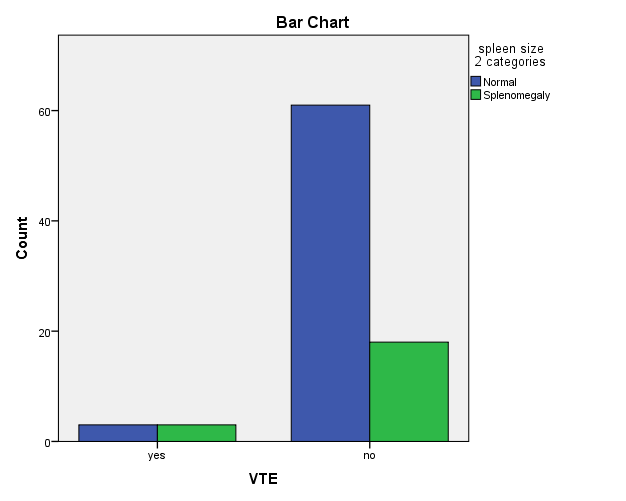


**Frequencies**

| **Statistics** | | |
| --- | --- | --- |
| Wound complication/Collection | | |
| N | Valid | 85 |
|  | Missing | 0 |

| **Wound complication/Collection** | | | | | |
| --- | --- | --- | --- | --- | --- |
|  | | Frequency | Percent | Valid Percent | Cumulative Percent |
| Valid | yes | 19 | 22.4 | 22.4 | 22.4 |
|  | no | 66 | 77.6 | 77.6 | 100.0 |
|  | Total | 85 | 100.0 | 100.0 |  |


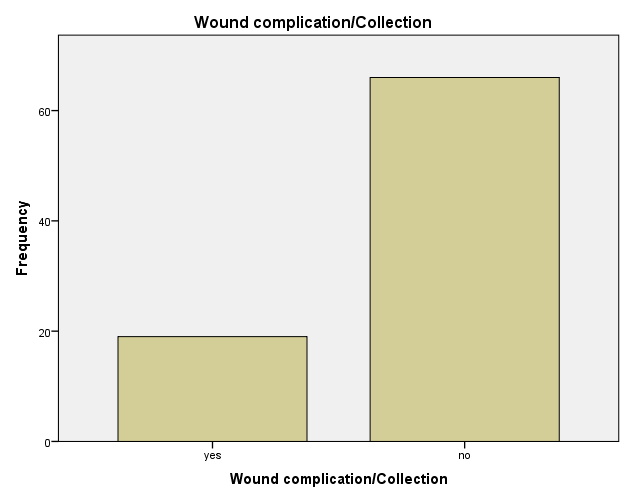


**Frequencies**

| **Statistics** | | |
| --- | --- | --- |
| Post -op PLT | | |
| N | Valid | 85 |
|  | Missing | 0 |

| **Post -op PLT** | | | | | |
| --- | --- | --- | --- | --- | --- |
|  | | Frequency | Percent | Valid Percent | Cumulative Percent |
| Valid | <450 x 10^3 | 23 | 27.1 | 27.1 | 27.1 |
|  | >450 x 10^3 | 41 | 48.2 | 48.2 | 75.3 |
|  | >1000 x 10^3 | 21 | 24.7 | 24.7 | 100.0 |
|  | Total | 85 | 100.0 | 100.0 |  |


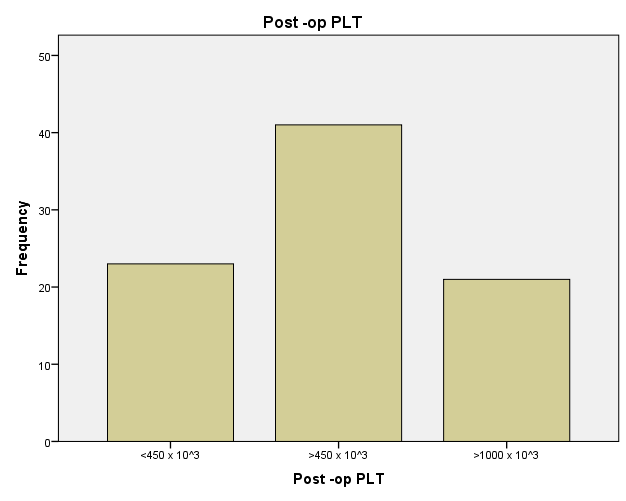


**Crosstabs**

| **Case Processing Summary** | | | | | | |
| --- | --- | --- | --- | --- | --- | --- |
|  | Cases | | | | | |
|  | Valid | | Missing | | Total | |
|  | N | Percent | N | Percent | N | Percent |
| VTE * post-op plt changed | 85 | 100.0% | 0 | 0.0% | 85 | 100.0% |

| **VTE * post-op plt changed Crosstabulation** | | | | | |
| --- | --- | --- | --- | --- | --- |
|  | | | post-op plt changed | | Total |
|  |  |  | <450 x 10^3 | >450 x 10^3 |  |
| VTE | yes | Count | 2 | 4 | 6 |
|  |  | Expected Count | 1.6 | 4.4 | 6.0 |
|  |  | % within post-op plt changed | 8.7% | 6.5% | 7.1% |
|  | no | Count | 21 | 58 | 79 |
|  |  | Expected Count | 21.4 | 57.6 | 79.0 |
|  |  | % within post-op plt changed | 91.3% | 93.5% | 92.9% |
| Total | | Count | 23 | 62 | 85 |
|  |  | Expected Count | 23.0 | 62.0 | 85.0 |
|  |  | % within post-op plt changed | 100.0% | 100.0% | 100.0% |

| **Chi-Square Tests** | | | | | | |
| --- | --- | --- | --- | --- | --- | --- |
|  | Value | df | Asymp. Sig. (2-sided) | Exact Sig. (2-sided) | Exact Sig. (1-sided) | Point Probability |
| Pearson Chi-Square | .129^a^ | 1 | .720 | 1.000 | .519 |  |
| Continuity Correction^b^ | .000 | 1 | 1.000 |  |  |  |
| Likelihood Ratio | .124 | 1 | .725 | 1.000 | .519 |  |
| Fisher's Exact Test |  |  |  | .660 | .519 |  |
| Linear-by-Linear Association | .127^c^ | 1 | .721 | 1.000 | .519 | .323 |
| N of Valid Cases | 85 |  |  |  |  |  |
| a. 2 cells (50.0%) have expected count less than 5. The minimum expected count is 1.62. | | | | | | |
| b. Computed only for a 2x2 table | | | | | | |
| c. The standardized statistic is .357. | | | | | | |

| **Symmetric Measures** | | | | |
| --- | --- | --- | --- | --- |
|  | | Value | Approx. Sig. | Exact Sig. |
| Nominal by Nominal | Phi | .039 | .720 | 1.000 |
|  | Cramer's V | .039 | .720 | 1.000 |
| N of Valid Cases | | 85 |  |  |

| **Risk Estimate** | | | |
| --- | --- | --- | --- |
|  | Value | 95% Confidence Interval | |
|  |  | Lower | Upper |
| Odds Ratio for VTE (yes / no) | 1.381 | .235 | 8.101 |
| For cohort post-op plt changed = <450 x 10^3 | 1.254 | .382 | 4.120 |
| For cohort post-op plt changed = >450 x 10^3 | .908 | .508 | 1.624 |
| N of Valid Cases | 85 |  |  |


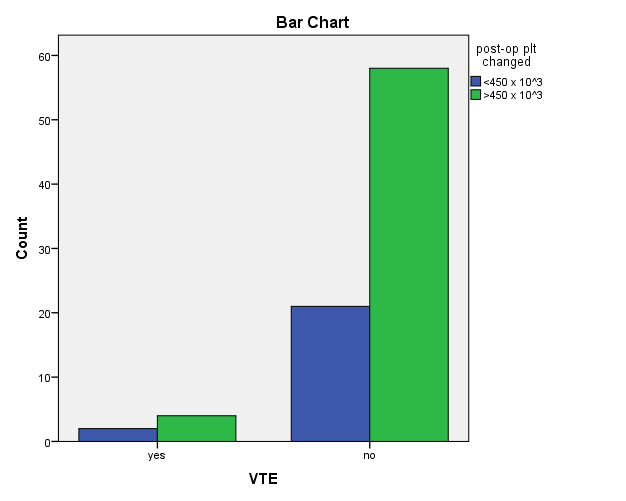


**Crosstabs**

| **Case Processing Summary** | | | | | | |
| --- | --- | --- | --- | --- | --- | --- |
|  | Cases | | | | | |
|  | Valid | | Missing | | Total | |
|  | N | Percent | N | Percent | N | Percent |
| VTE * post-op plt changed | 85 | 100.0% | 0 | 0.0% | 85 | 100.0% |

| **VTE * post-op plt changed Crosstabulation** | | | | | |
| --- | --- | --- | --- | --- | --- |
|  | | | post-op plt changed | | Total |
|  |  |  | <1000 x 10^3 | >1000 x 10^3 |  |
| VTE | yes | Count | 5 | 1 | 6 |
|  |  | Expected Count | 4.5 | 1.5 | 6.0 |
|  |  | % within post-op plt changed | 7.8% | 4.8% | 7.1% |
|  | no | Count | 59 | 20 | 79 |
|  |  | Expected Count | 59.5 | 19.5 | 79.0 |
|  |  | % within post-op plt changed | 92.2% | 95.2% | 92.9% |
| Total | | Count | 64 | 21 | 85 |
|  |  | Expected Count | 64.0 | 21.0 | 85.0 |
|  |  | % within post-op plt changed | 100.0% | 100.0% | 100.0% |

| **Chi-Square Tests** | | | | | | |
| --- | --- | --- | --- | --- | --- | --- |
|  | Value | df | Asymp. Sig. (2-sided) | Exact Sig. (2-sided) | Exact Sig. (1-sided) | Point Probability |
| Pearson Chi-Square | .224^a^ | 1 | .636 | 1.000 | .538 |  |
| Continuity Correction^b^ | .000 | 1 | 1.000 |  |  |  |
| Likelihood Ratio | .243 | 1 | .622 | .695 | .538 |  |
| Fisher's Exact Test |  |  |  | 1.000 | .538 |  |
| Linear-by-Linear Association | .222^c^ | 1 | .638 | 1.000 | .538 | .366 |
| N of Valid Cases | 85 |  |  |  |  |  |
| a. 2 cells (50.0%) have expected count less than 5. The minimum expected count is 1.48. | | | | | | |
| b. Computed only for a 2x2 table | | | | | | |
| c. The standardized statistic is .471. | | | | | | |

| **Symmetric Measures** | | | | |
| --- | --- | --- | --- | --- |
|  | | Value | Approx. Sig. | Exact Sig. |
| Nominal by Nominal | Phi | .051 | .636 | 1.000 |
|  | Cramer's V | .051 | .636 | 1.000 |
| N of Valid Cases | | 85 |  |  |

| **Risk Estimate** | | | |
| --- | --- | --- | --- |
|  | Value | 95% Confidence Interval | |
|  |  | Lower | Upper |
| Odds Ratio for VTE (yes / no) | 1.695 | .187 | 15.390 |
| For cohort post-op plt changed = <1000 x 10^3 | 1.116 | .763 | 1.632 |
| For cohort post-op plt changed = >1000 x 10^3 | .658 | .106 | 4.099 |
| N of Valid Cases | 85 |  |  |


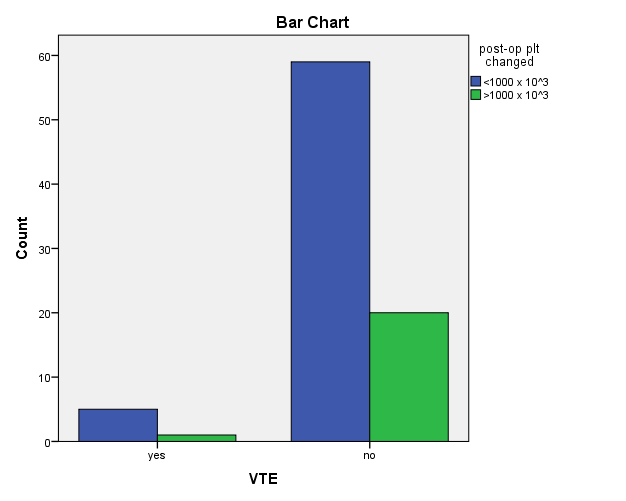


**Crosstabs**

| **Case Processing Summary** | | | | | | |
| --- | --- | --- | --- | --- | --- | --- |
|  | Cases | | | | | |
|  | Valid | | Missing | | Total | |
|  | N | Percent | N | Percent | N | Percent |
| VTE * comorbidities | 85 | 100.0% | 0 | 0.0% | 85 | 100.0% |

| **VTE * comorbidities Crosstabulation** | | | | | |
| --- | --- | --- | --- | --- | --- |
|  | | | comorbidities | | Total |
|  |  |  | <3 | >=3 |  |
| VTE | yes | Count | 4 | 2 | 6 |
|  |  | Expected Count | 4.0 | 2.0 | 6.0 |
|  |  | % within comorbidities | 7.1% | 6.9% | 7.1% |
|  | no | Count | 52 | 27 | 79 |
|  |  | Expected Count | 52.0 | 27.0 | 79.0 |
|  |  | % within comorbidities | 92.9% | 93.1% | 92.9% |
| Total | | Count | 56 | 29 | 85 |
|  |  | Expected Count | 56.0 | 29.0 | 85.0 |
|  |  | % within comorbidities | 100.0% | 100.0% | 100.0% |

| **Chi-Square Tests** | | | | | | |
| --- | --- | --- | --- | --- | --- | --- |
|  | Value | df | Asymp. Sig. (2-sided) | Exact Sig. (2-sided) | Exact Sig. (1-sided) | Point Probability |
| Pearson Chi-Square | .002^a^ | 1 | .966 | 1.000 | .668 |  |
| Continuity Correction^b^ | .000 | 1 | 1.000 |  |  |  |
| Likelihood Ratio | .002 | 1 | .966 | 1.000 | .668 |  |
| Fisher's Exact Test |  |  |  | 1.000 | .668 |  |
| Linear-by-Linear Association | .002^c^ | 1 | .967 | 1.000 | .668 | .341 |
| N of Valid Cases | 85 |  |  |  |  |  |
| a. 2 cells (50.0%) have expected count less than 5. The minimum expected count is 2.05. | | | | | | |
| b. Computed only for a 2x2 table | | | | | | |
| c. The standardized statistic is .042. | | | | | | |

| **Symmetric Measures** | | | | |
| --- | --- | --- | --- | --- |
|  | | Value | Approx. Sig. | Exact Sig. |
| Nominal by Nominal | Phi | .005 | .966 | 1.000 |
|  | Cramer's V | .005 | .966 | 1.000 |
| N of Valid Cases | | 85 |  |  |

| **Risk Estimate** | | | |
| --- | --- | --- | --- |
|  | Value | 95% Confidence Interval | |
|  |  | Lower | Upper |
| Odds Ratio for VTE (yes / no) | 1.038 | .179 | 6.035 |
| For cohort comorbidities = <3 | 1.013 | .563 | 1.823 |
| For cohort comorbidities = >=3 | .975 | .302 | 3.149 |
| N of Valid Cases | 85 |  |  |


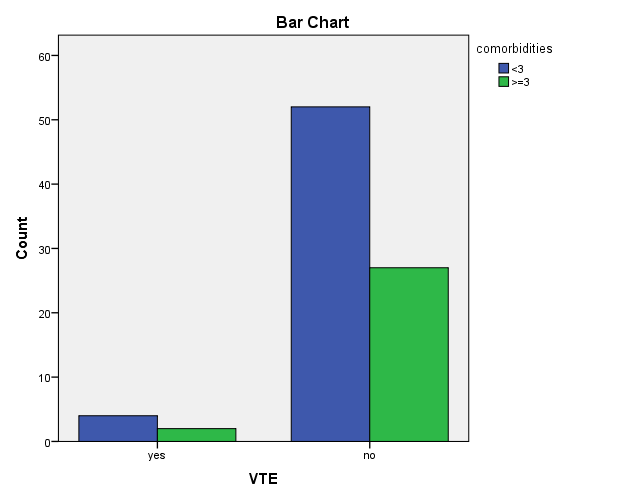

Supplement: Multimedia component 3 [file mmc3.docx]
