# Supplementary material for: Evaluation of the risk factors for venous thromboembolism post splenectomy – A ten year retrospective cohort study in St James’s hospital
Source: Ann Med Surg (Lond). 2021 May 8;66:102381. doi: 10.1016/j.amsu.2021.102381 (PMC8131975; doi:10.1016/j.amsu.2021.102381)
Supplement: Multimedia component 4 [file mmc4.doc]

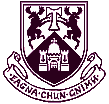


UNIVERSITY *of* LIMERICK

OLLSCOIL LUIMNIGH

STATISTICAL CONSULTING UNIT

Report on the Analyses of Splenectomy data

(Manal Alsayed)

Produced by Dr Jean Saunders

Executive Director

Statistical Consulting Unit

Introduction

This report summarises the analyses carried out on the study data provided by Manal Alsayed.

### Results

The full results are given in the ‘summaries’ and ‘analyses’ word files. There were 85 patients in total. 6/85 had a VTE (7.1%).

Firstly the variables were all summarised and these results are in the ‘summaries’ file. The most common age groups were 18-29 and 50-65. There were 41 females and 44 males. 25/34 had a BMI <30. All of the other categorical variables have been summarised with tables and bar charts. The scalar variables were plotted and summarised. Duration was negatively skewed and had a median of 2.9 inter-quartile range (IQR) (2.3. 3.5). Blood loss had a positive skew median 5 IQR (80, 1995). Hospital stay had a positive skew median 11 IQR (7, 22.5). Post-op days was reasonably normal with a mean 10.44 SD 5.75. CCI had a positive skew with a median 1 IQR (0, 3).

All of the analyses can be found in the ‘analyses’ file. Cross-tabulations and chi-squared and fishers exact tests (FET) were carried out to see if there was a significant association between the factor and VTE’s. For the scalar factors the distribution were tested for significant difference between VTE yes or no. Of the categorical factors only BMI and anticoagulation was significantly different between VTE categories yes/no with for BMI, p=0.007, Fishers exact test. More >=30 were classified as having a VTE than expected by chance. For anticoagulation p=0.005, FET with more N/As having a VTE and all those having anticoagulation not having a VTE. All other categorical risk factors did not have a significant association with VTE status. None of the scalar factors tested as significantly different between VTE groups although the ‘no’ group tended to have higher values for most of these factors (see box plots). A logistic regression was attempted to predict VTE from some of the factors, unfortunately none of the risk factors tried were significant after adjustment for age and gender.

##### Conclusion

All Significant results have been described above.

Signed: Jean Saunders
