# Supplementary material for: Evaluation of the risk factors for venous thromboembolism post splenectomy – A ten year retrospective cohort study in St James’s hospital
Source: Ann Med Surg (Lond). 2021 May 8;66:102381. doi: 10.1016/j.amsu.2021.102381 (PMC8131975; doi:10.1016/j.amsu.2021.102381)

**Frequencies**

| **Statistics** | | | | | |  |  |  |
| --- | --- | --- | --- | --- | --- | --- | --- | --- |
| Age | | | | | |  |  |  |
| N | | Valid | | 85 | |  |  |  |
|  |  | Missing | | 0 | |  |  |  |
| **Age** | | | | | | | | |
|  | | | Frequency | | Percent | | Valid Percent | Cumulative Percent |
| Valid | <18 | | 2 | | 2.4 | | 2.4 | 2.4 |
|  | 18-29 | | 23 | | 27.1 | | 27.1 | 29.4 |
|  | 30-49 | | 21 | | 24.7 | | 24.7 | 54.1 |
|  | 50-65 | | 23 | | 27.1 | | 27.1 | 81.2 |
|  | >65 | | 16 | | 18.8 | | 18.8 | 100.0 |
|  | Total | | 85 | | 100.0 | | 100.0 |  |


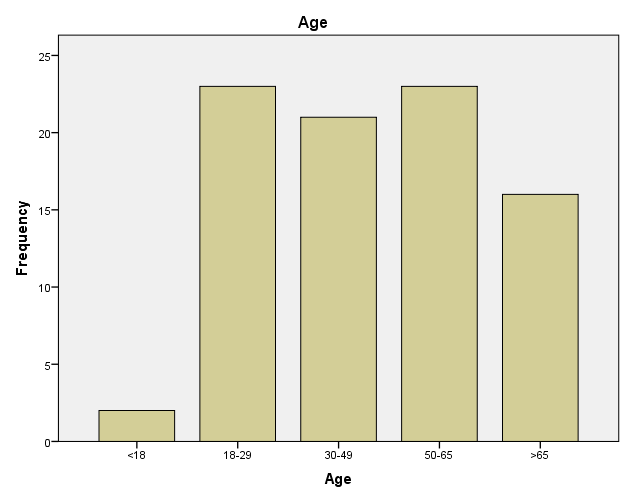


**Frequencies**

| **Statistics** | | |
| --- | --- | --- |
| Gender | | |
| N | Valid | 85 |
|  | Missing | 0 |

| **Gender** | | | | | |
| --- | --- | --- | --- | --- | --- |
|  | | Frequency | Percent | Valid Percent | Cumulative Percent |
| Valid | male | 44 | 51.8 | 51.8 | 51.8 |
|  | female | 41 | 48.2 | 48.2 | 100.0 |
|  | Total | 85 | 100.0 | 100.0 |  |


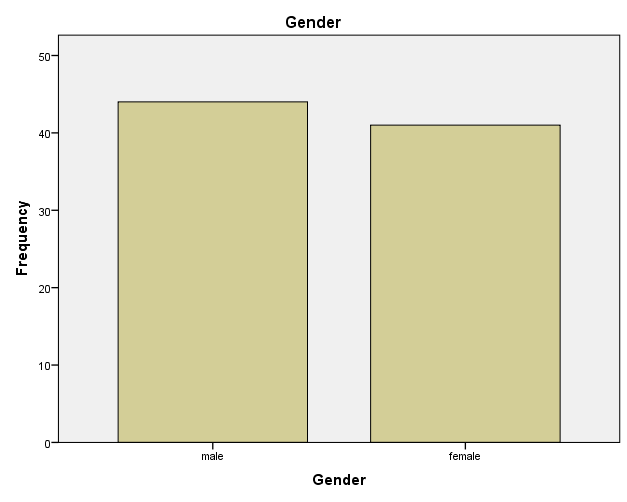


**Frequencies**

| **Statistics** | | | | |  |  |  |  |
| --- | --- | --- | --- | --- | --- | --- | --- | --- |
| BMI | | | | |  |  |  |  |
| N | | Valid | 85 | |  |  |  |  |
|  |  | Missing | 0 | |  |  |  |  |
| **BMI** | | | | | | | | |
|  | | | | Frequency | | Percent | Valid Percent | Cumulative Percent |
| Valid | <30 | | | 25 | | 29.4 | 29.4 | 29.4 |
|  | >=30 | | | 9 | | 10.6 | 10.6 | 40.0 |
|  | not mentioned | | | 51 | | 60.0 | 60.0 | 100.0 |
|  | Total | | | 85 | | 100.0 | 100.0 |  |


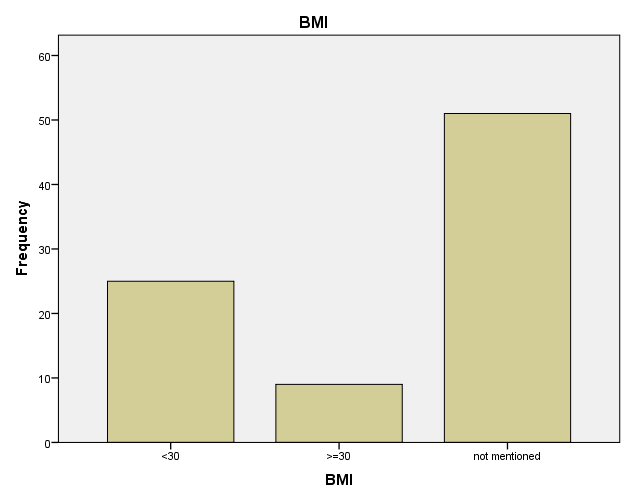


**Frequencies**

| **Statistics** | | | | | |  |  |  |
| --- | --- | --- | --- | --- | --- | --- | --- | --- |
| OCP | | | | | |  |  |  |
| N | | Valid | | 85 | |  |  |  |
|  |  | Missing | | 0 | |  |  |  |
| **OCP** | | | | | | | | |
|  | | | Frequency | | Percent | | Valid Percent | Cumulative Percent |
| Valid | yes | | 3 | | 3.5 | | 3.5 | 3.5 |
|  | no | | 39 | | 45.9 | | 45.9 | 49.4 |
|  | N/A | | 43 | | 50.6 | | 50.6 | 100.0 |
|  | Total | | 85 | | 100.0 | | 100.0 |  |


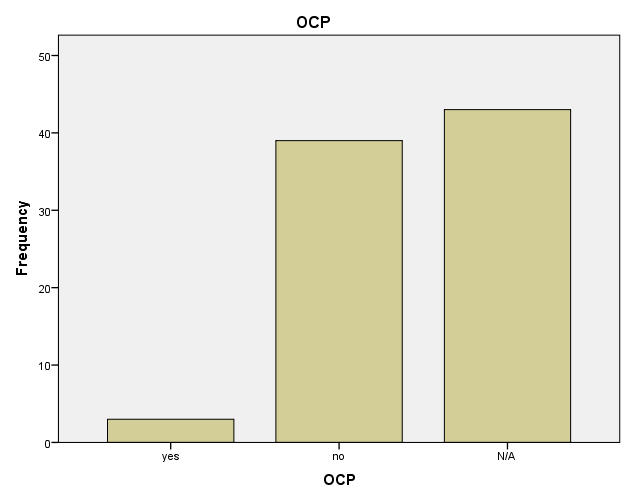


**Frequencies**

| **Statistics** | | | | | |  |  |  |
| --- | --- | --- | --- | --- | --- | --- | --- | --- |
| Active Cancer | | | | | |  |  |  |
| N | | Valid | | 85 | |  |  |  |
|  |  | Missing | | 0 | |  |  |  |
| **Active Cancer** | | | | | | | | |
|  | | | Frequency | | Percent | | Valid Percent | Cumulative Percent |
| Valid | yes | | 26 | | 30.6 | | 30.6 | 30.6 |
|  | no | | 59 | | 69.4 | | 69.4 | 100.0 |
|  | Total | | 85 | | 100.0 | | 100.0 |  |


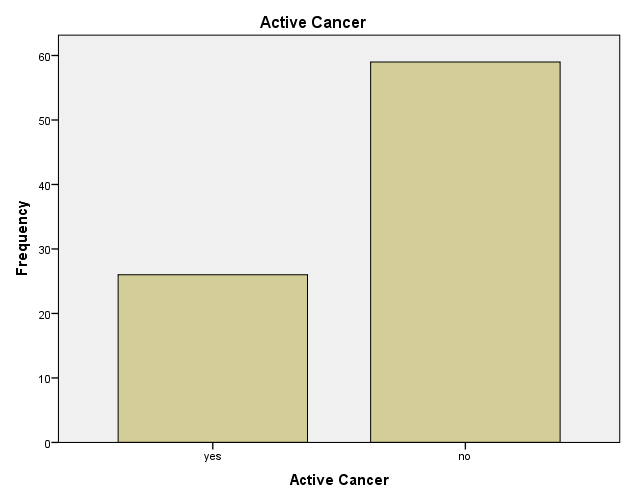


**Frequencies**

| **Statistics** | | | | | |  |  |  |
| --- | --- | --- | --- | --- | --- | --- | --- | --- |
| Chronic Liver Disease (CLD) | | | | | |  |  |  |
| N | | Valid | | 85 | |  |  |  |
|  |  | Missing | | 0 | |  |  |  |
| **Chronic Liver Disease (CLD)** | | | | | | | | |
|  | | | Frequency | | Percent | | Valid Percent | Cumulative Percent |
| Valid | yes | | 2 | | 2.4 | | 2.4 | 2.4 |
|  | no | | 83 | | 97.6 | | 97.6 | 100.0 |
|  | Total | | 85 | | 100.0 | | 100.0 |  |


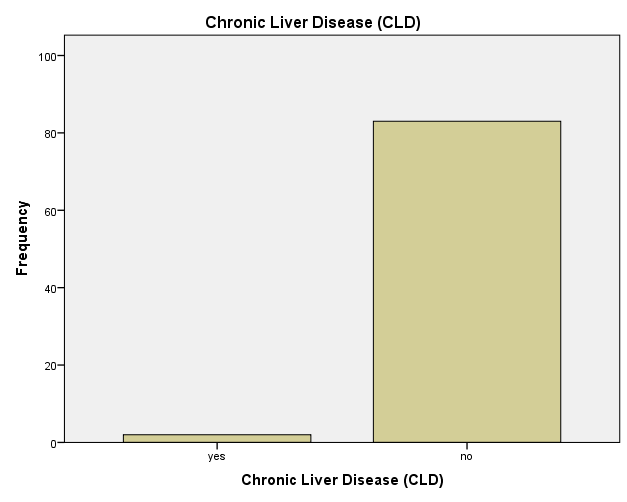


**Frequencies**

**Pre-operative platelets**

| **Statistics** | | | |  |  |  |  |
| --- | --- | --- | --- | --- | --- | --- | --- |
| Pre-operative platelets | | | |  |  |  |  |
| N | | Valid | 85 |  |  |  |  |
|  |  | Missing | 0 |  |  |  |  |
| **Pre-operative platelets** | | | | | | | |
|  | | | Frequency | | Percent | Valid Percent | Cumulative Percent |
| Valid | 50-99 | | 11 | | 12.9 | 12.9 | 12.9 |
|  | 100-450 | | 68 | | 80.0 | 80.0 | 92.9 |
|  | >450 | | 6 | | 7.1 | 7.1 | 100.0 |
|  | Total | | 85 | | 100.0 | 100.0 |  |


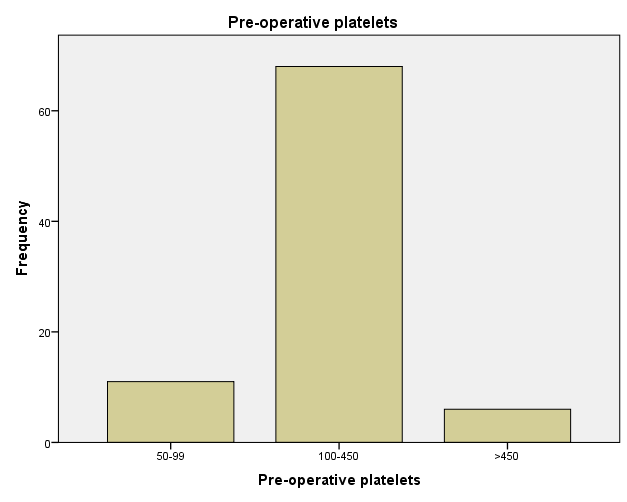


**Antiplatelets**

| **Statistics** | | | | | |  |  |  |
| --- | --- | --- | --- | --- | --- | --- | --- | --- |
| Antiplatelets | | | | | |  |  |  |
| N | | Valid | | 85 | |  |  |  |
|  |  | Missing | | 0 | |  |  |  |
| **Antiplatelets** | | | | | | | | |
|  | | | Frequency | | Percent | | Valid Percent | Cumulative Percent |
| Valid | yes | | 11 | | 12.9 | | 12.9 | 12.9 |
|  | no | | 74 | | 87.1 | | 87.1 | 100.0 |
|  | Total | | 85 | | 100.0 | | 100.0 |  |


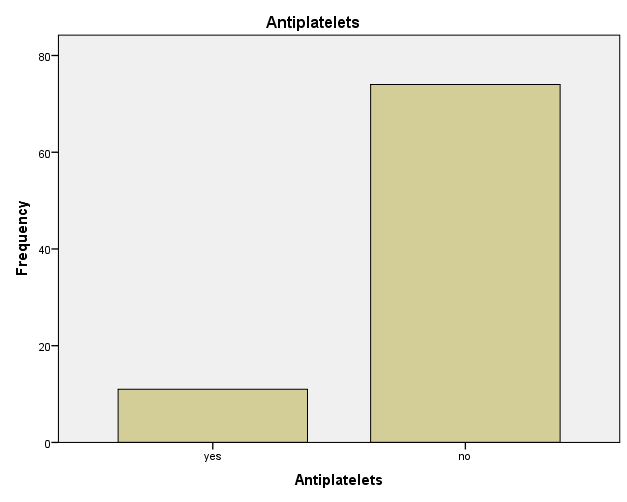


**Anticoagulation**

| **Statistics** | | | | | |  |  |  |
| --- | --- | --- | --- | --- | --- | --- | --- | --- |
| Anticoagulation | | | | | |  |  |  |
| N | | Valid | | 85 | |  |  |  |
|  |  | Missing | | 0 | |  |  |  |
| **Anticoagulation** | | | | | | | | |
|  | | | Frequency | | Percent | | Valid Percent | Cumulative Percent |
| Valid | yes | | 15 | | 17.6 | | 17.6 | 17.6 |
|  | no | | 70 | | 82.4 | | 82.4 | 100.0 |
|  | Total | | 85 | | 100.0 | | 100.0 |  |


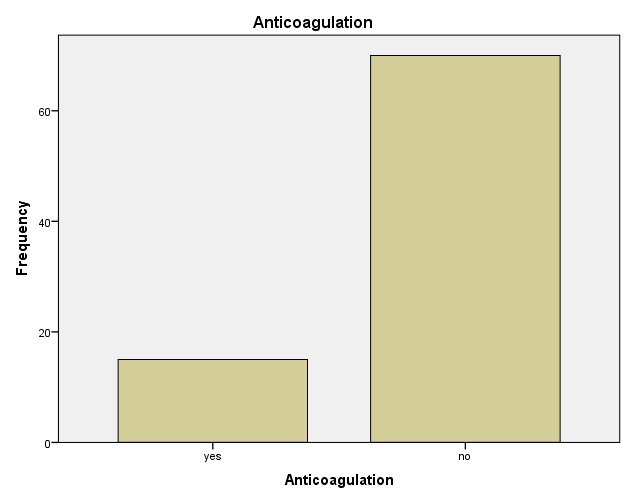


**Frequencies**

**Vaccination**

| **Statistics** | | | | |  |  |  |  |
| --- | --- | --- | --- | --- | --- | --- | --- | --- |
| Vaccination | | | | |  |  |  |  |
| N | | Valid | 85 | |  |  |  |  |
|  |  | Missing | 0 | |  |  |  |  |
| **Vaccination** | | | | | | | | |
|  | | | | Frequency | | Percent | Valid Percent | Cumulative Percent |
| Valid | pre-operative | | | 32 | | 37.6 | 37.6 | 37.6 |
|  | post-operative | | | 44 | | 51.8 | 51.8 | 89.4 |
|  | not mentioned | | | 9 | | 10.6 | 10.6 | 100.0 |
|  | Total | | | 85 | | 100.0 | 100.0 |  |


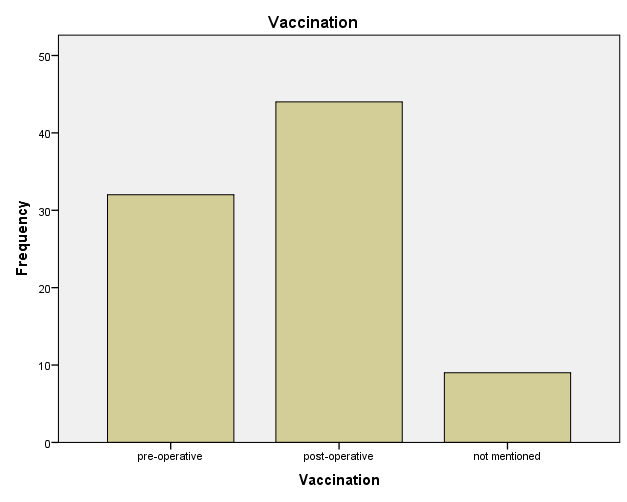


**Referring Dr**

| **Statistics** | | | | |  |  |  |  |
| --- | --- | --- | --- | --- | --- | --- | --- | --- |
| Referring Dr | | | | |  |  |  |  |
| N | | Valid | 85 | |  |  |  |  |
|  |  | Missing | 0 | |  |  |  |  |
| **Referring Dr** | | | | | | | | |
|  | | | | Frequency | | Percent | Valid Percent | Cumulative Percent |
| Valid | Haematology | | | 39 | | 45.9 | 45.9 | 45.9 |
|  | Oncology | | | 10 | | 11.8 | 11.8 | 57.6 |
|  | Upper GI | | | 30 | | 35.3 | 35.3 | 92.9 |
|  | others | | | 6 | | 7.1 | 7.1 | 100.0 |
|  | Total | | | 85 | | 100.0 | 100.0 |  |


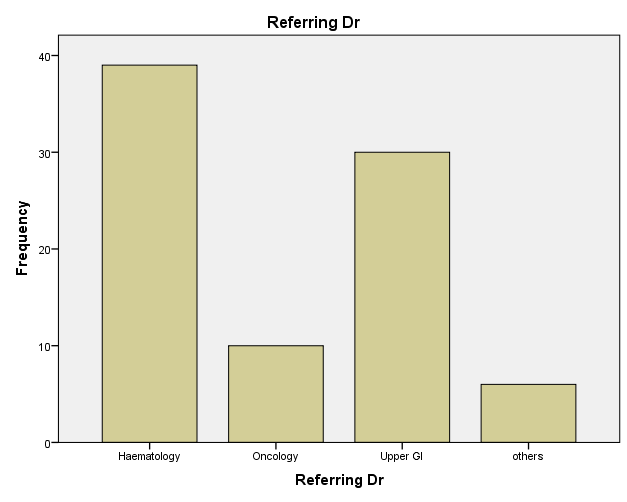


**Indication**

| **Statistics** | | | |  |  |  |  |  |
| --- | --- | --- | --- | --- | --- | --- | --- | --- |
| Indication | | | |  |  |  |  |  |
| N | | Valid | 85 |  |  |  |  |  |
|  |  | Missing | 0 |  |  |  |  |  |
| **Indication** | | | | | | | | |
|  | | | | | Frequency | Percent | Valid Percent | Cumulative Percent |
| Valid | Benign haematology | | | | 29 | 34.1 | 34.1 | 34.1 |
|  | Malignant haematology | | | | 11 | 12.9 | 12.9 | 47.1 |
|  | Solid tumors | | | | 9 | 10.6 | 10.6 | 57.6 |
|  | Spontaneous rupture | | | | 4 | 4.7 | 4.7 | 62.4 |
|  | Traumatic rupture | | | | 14 | 16.5 | 16.5 | 78.8 |
|  | Iatrogenic trauma | | | | 9 | 10.6 | 10.6 | 89.4 |
|  | Others | | | | 9 | 10.6 | 10.6 | 100.0 |
|  | Total | | | | 85 | 100.0 | 100.0 |  |


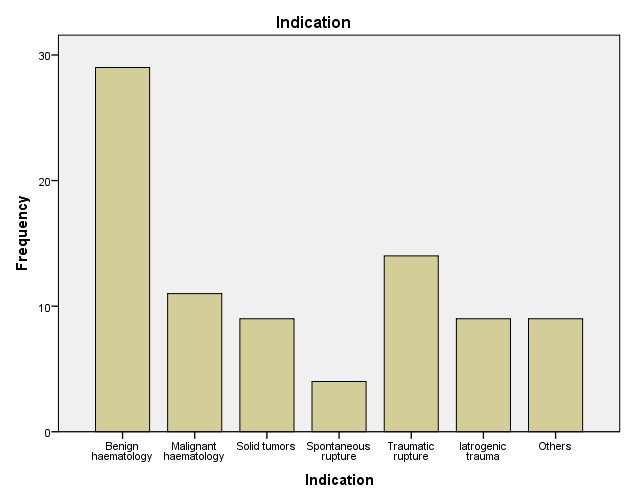


**Frequencies**

**Date of Surgery**

| **Statistics** | | | | | |  |  |  |
| --- | --- | --- | --- | --- | --- | --- | --- | --- |
| Date of Surgery | | | | | |  |  |  |
| N | | Valid | | 85 | |  |  |  |
|  |  | Missing | | 0 | |  |  |  |
| **Date of Surgery** | | | | | | | | |
|  | | | Frequency | | Percent | | Valid Percent | Cumulative Percent |
| Valid | 2007 | | 7 | | 8.2 | | 8.2 | 8.2 |
|  | 2008 | | 14 | | 16.5 | | 16.5 | 24.7 |
|  | 2009 | | 5 | | 5.9 | | 5.9 | 30.6 |
|  | 2010 | | 11 | | 12.9 | | 12.9 | 43.5 |
|  | 2011 | | 8 | | 9.4 | | 9.4 | 52.9 |
|  | 2012 | | 8 | | 9.4 | | 9.4 | 62.4 |
|  | 2013 | | 6 | | 7.1 | | 7.1 | 69.4 |
|  | 2014 | | 8 | | 9.4 | | 9.4 | 78.8 |
|  | 2015 | | 7 | | 8.2 | | 8.2 | 87.1 |
|  | 2016 | | 5 | | 5.9 | | 5.9 | 92.9 |
|  | 2017 | | 6 | | 7.1 | | 7.1 | 100.0 |
|  | Total | | 85 | | 100.0 | | 100.0 |  |


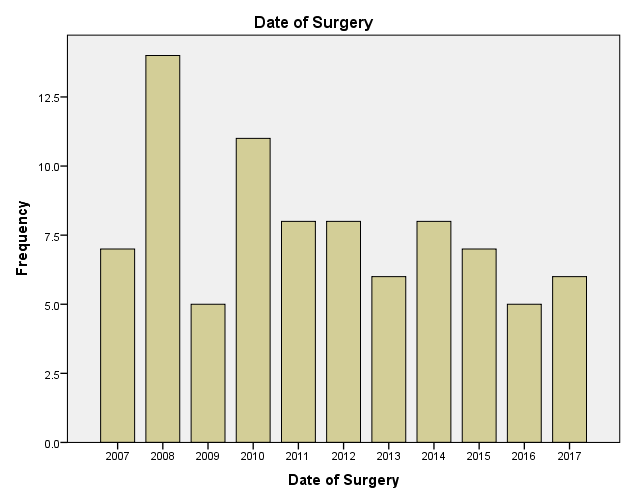


**Type**

| **Statistics** | | |
| --- | --- | --- |
| Type | | |
| N | Valid | 85 |
|  | Missing | 0 |

| **Type** | | | | | |
| --- | --- | --- | --- | --- | --- |
|  | | Frequency | Percent | Valid Percent | Cumulative Percent |
| Valid | Emergency | 30 | 35.3 | 35.3 | 35.3 |
|  | Elective | 55 | 64.7 | 64.7 | 100.0 |
|  | Total | 85 | 100.0 | 100.0 |  |


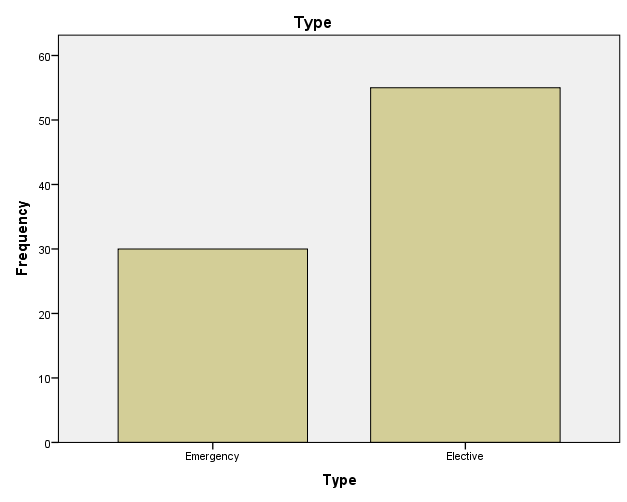


**Procedure**

| **Statistics** | | | | |  |  |  |  |
| --- | --- | --- | --- | --- | --- | --- | --- | --- |
| Procedure | | | | |  |  |  |  |
| N | | Valid | 85 | |  |  |  |  |
|  |  | Missing | 0 | |  |  |  |  |
| **Procedure** | | | | | | | | |
|  | | | | Frequency | | Percent | Valid Percent | Cumulative Percent |
| Valid | Open | | | 49 | | 57.6 | 57.6 | 57.6 |
|  | Laparoscopic | | | 36 | | 42.4 | 42.4 | 100.0 |
|  | Total | | | 85 | | 100.0 | 100.0 |  |


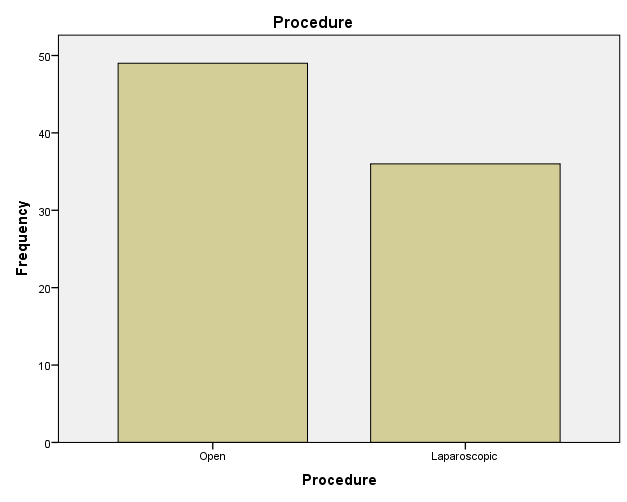


**Frequencies**

| **Statistics** | | | | |  |  |  |  |
| --- | --- | --- | --- | --- | --- | --- | --- | --- |
| Complexity | | | | |  |  |  |  |
| N | | Valid | 85 | |  |  |  |  |
|  |  | Missing | 0 | |  |  |  |  |
| **Complexity** | | | | | | | | |
|  | | | | Frequency | | Percent | Valid Percent | Cumulative Percent |
| Valid | Simple | | | 58 | | 68.2 | 68.2 | 68.2 |
|  | Complex | | | 27 | | 31.8 | 31.8 | 100.0 |
|  | Total | | | 85 | | 100.0 | 100.0 |  |


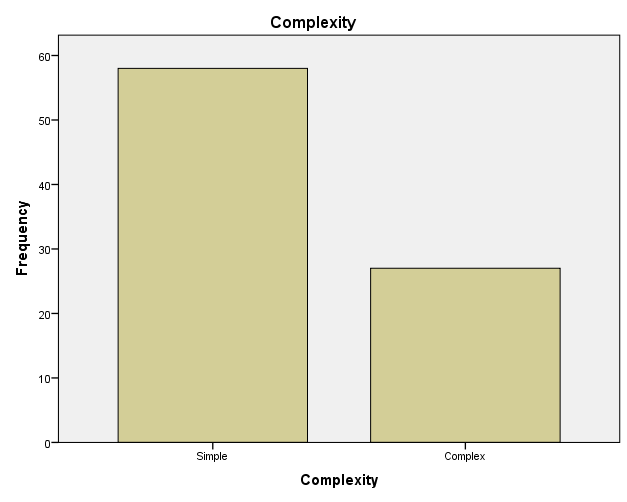


**Explore**

| **Case Processing Summary** | | | | | | | | | | |
| --- | --- | --- | --- | --- | --- | --- | --- | --- | --- | --- |
|  | Cases | | | | | | | | | |
|  | Valid | | Missing | | | | Total | | | |
|  | N | Percent | N | | Percent | | N | | Percent | |
| Duration | 84 | 98.8% | 1 | | 1.2% | | 85 | | 100.0% | |
| **Descriptives** | | | | | | | | | |  |
|  | | | | | | Statistic | | Std. Error | |  |
| Duration | Mean | | | | | 3.0357 | | .13669 | |  |
|  | 95% Confidence Interval for Mean | | | Lower Bound | | 2.7638 | |  | |  |
|  |  |  |  | Upper Bound | | 3.3076 | |  | |  |
|  | 5% Trimmed Mean | | | | | 2.9530 | |  | |  |
|  | Median | | | | | 2.8750 | |  | |  |
|  | Variance | | | | | 1.569 | |  | |  |
|  | Std. Deviation | | | | | 1.25279 | |  | |  |
|  | Minimum | | | | | 1.00 | |  | |  |
|  | Maximum | | | | | 8.00 | |  | |  |
|  | Range | | | | | 7.00 | |  | |  |
|  | Interquartile Range | | | | | 1.25 | |  | |  |
|  | Skewness | | | | | 1.252 | | .263 | |  |
|  | Kurtosis | | | | | 2.871 | | .520 | |  |

| **Percentiles** | | | | | | | | |
| --- | --- | --- | --- | --- | --- | --- | --- | --- |
|  | | Percentiles | | | | | | |
|  |  | 5 | 10 | 25 | 50 | 75 | 90 | 95 |
| Weighted Average(Definition 1) | Duration | 1.3125 | 1.7500 | 2.2500 | 2.8750 | 3.5000 | 4.6250 | 4.9375 |
| Tukey's Hinges | Duration |  |  | 2.2500 | 2.8750 | 3.5000 |  |  |

**Duration**


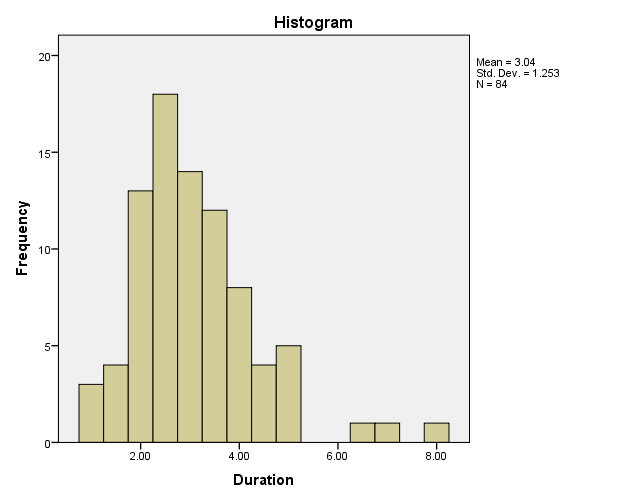


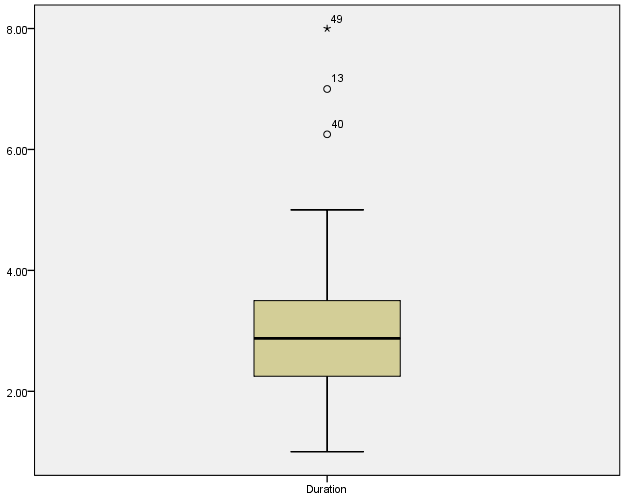


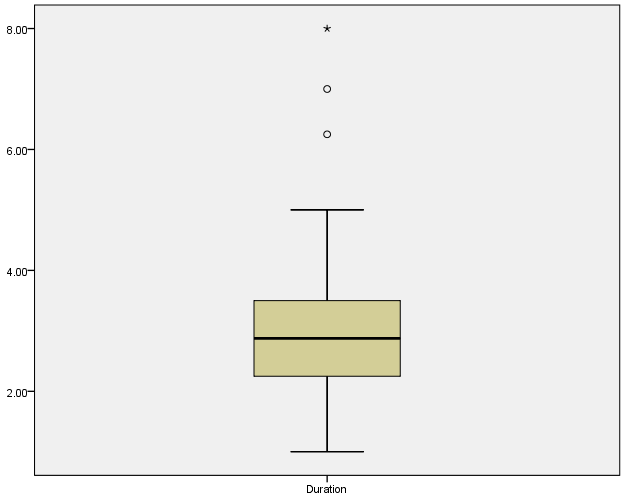


**Explore**

| **Case Processing Summary** | | | | | | | | | |  |
| --- | --- | --- | --- | --- | --- | --- | --- | --- | --- | --- |
|  | Cases | | | | | | | | |  |
|  | Valid | | Missing | | | | Total | | |  |
|  | N | Percent | N | | Percent | | N | Percent | |  |
| Blood Loss/ml | 84 | 98.8% | 1 | | 1.2% | | 85 | 100.0% | |  |
| **Descriptives** | | | | | | | | | | |
|  | | | | | | Statistic | | | Std. Error | |
| Blood Loss/ml | Mean | | | | | 1556.40 | | | 265.058 | |
|  | 95% Confidence Interval for Mean | | | Lower Bound | | 1029.22 | | |  | |
|  |  |  |  | Upper Bound | | 2083.59 | | |  | |
|  | 5% Trimmed Mean | | | | | 1185.13 | | |  | |
|  | Median | | | | | 500.00 | | |  | |
|  | Variance | | | | | 5901469.256 | | |  | |
|  | Std. Deviation | | | | | 2429.294 | | |  | |
|  | Minimum | | | | | 50 | | |  | |
|  | Maximum | | | | | 14600 | | |  | |
|  | Range | | | | | 14550 | | |  | |
|  | Interquartile Range | | | | | 1915 | | |  | |
|  | Skewness | | | | | 2.917 | | | .263 | |
|  | Kurtosis | | | | | 10.755 | | | .520 | |

| **Percentiles** | | | | | | | | |
| --- | --- | --- | --- | --- | --- | --- | --- | --- |
|  | | Percentiles | | | | | | |
|  |  | 5 | 10 | 25 | 50 | 75 | 90 | 95 |
| Weighted Average(Definition 1) | Blood Loss/ml | 50.00 | 50.00 | 80.00 | 500.00 | 1995.00 | 4040.00 | 7290.00 |
| Tukey's Hinges | Blood Loss/ml |  |  | 80.00 | 500.00 | 1990.00 |  |  |

**Blood Loss/ml**


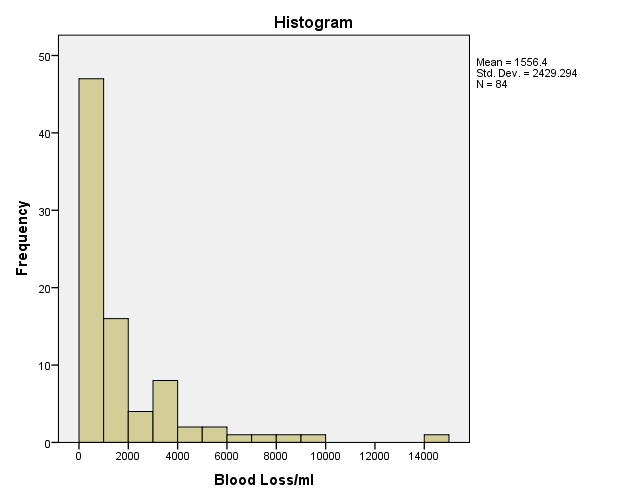


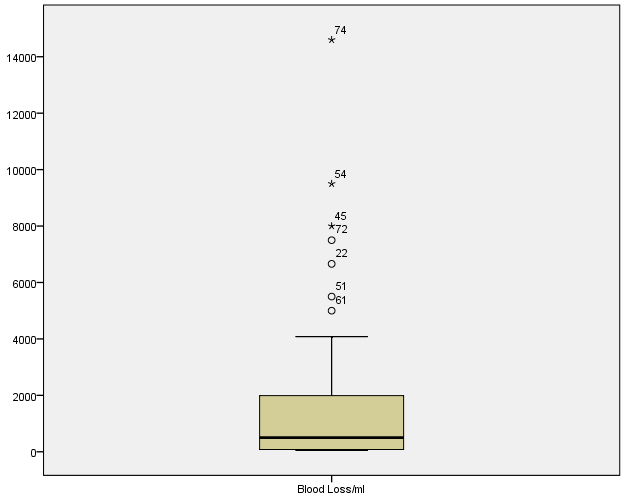


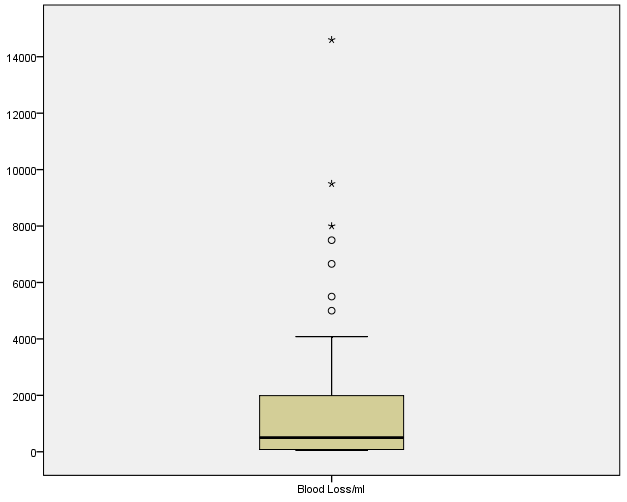


**Frequencies**

**Blood Tx**

| **Statistics** | | | | | |  |  |  |
| --- | --- | --- | --- | --- | --- | --- | --- | --- |
| Blood Tx | | | | | |  |  |  |
| N | | Valid | | 85 | |  |  |  |
|  |  | Missing | | 0 | |  |  |  |
| **Blood Tx** | | | | | | | | |
|  | | | Frequency | | Percent | | Valid Percent | Cumulative Percent |
| Valid | yes | | 34 | | 40.0 | | 40.0 | 40.0 |
|  | no | | 51 | | 60.0 | | 60.0 | 100.0 |
|  | Total | | 85 | | 100.0 | | 100.0 |  |


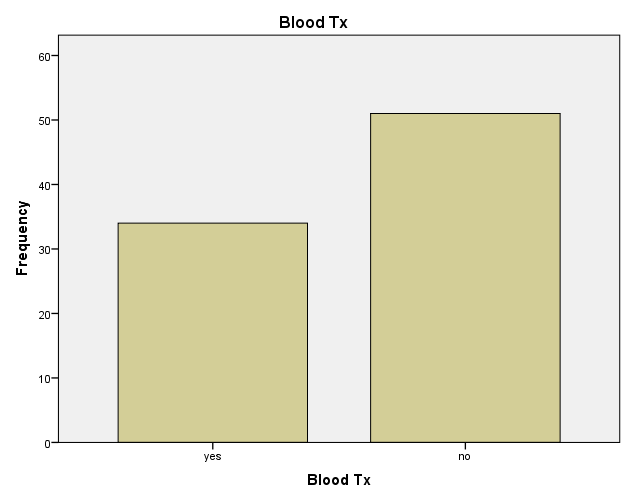


**Plts Tx**

| **Statistics** | | | | | |  |  |  |
| --- | --- | --- | --- | --- | --- | --- | --- | --- |
| Plts Tx | | | | | |  |  |  |
| N | | Valid | | 85 | |  |  |  |
|  |  | Missing | | 0 | |  |  |  |
| **Plts Tx** | | | | | | | | |
|  | | | Frequency | | Percent | | Valid Percent | Cumulative Percent |
| Valid | yes | | 15 | | 17.6 | | 17.6 | 17.6 |
|  | no | | 70 | | 82.4 | | 82.4 | 100.0 |
|  | Total | | 85 | | 100.0 | | 100.0 |  |


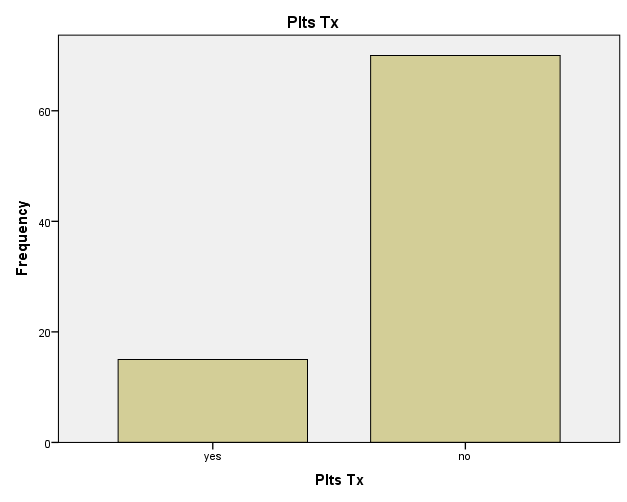


**Plasma**

| **Statistics** | | | | | |  |  |  |
| --- | --- | --- | --- | --- | --- | --- | --- | --- |
| Plasma | | | | | |  |  |  |
| N | | Valid | | 85 | |  |  |  |
|  |  | Missing | | 0 | |  |  |  |
| **Plasma** | | | | | | | | |
|  | | | Frequency | | Percent | | Valid Percent | Cumulative Percent |
| Valid | yes | | 14 | | 16.5 | | 16.5 | 16.5 |
|  | no | | 71 | | 83.5 | | 83.5 | 100.0 |
|  | Total | | 85 | | 100.0 | | 100.0 |  |


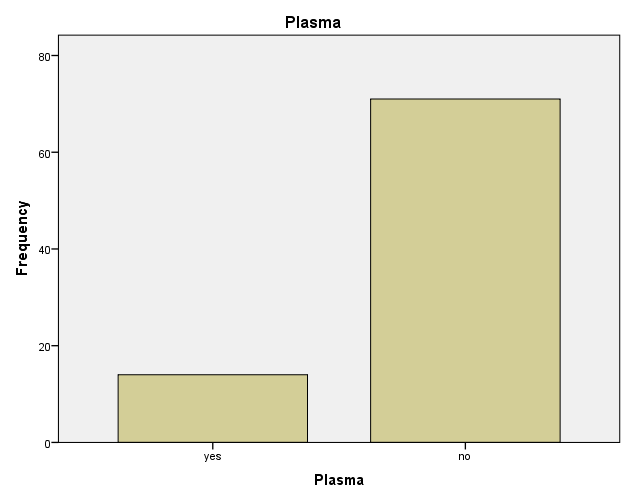


**Frequencies**

**Fibrinogen**

| **Statistics** | | | | | |  |  |  |
| --- | --- | --- | --- | --- | --- | --- | --- | --- |
| Fibrinogen | | | | | |  |  |  |
| N | | Valid | | 85 | |  |  |  |
|  |  | Missing | | 0 | |  |  |  |
| **Fibrinogen** | | | | | | | | |
|  | | | Frequency | | Percent | | Valid Percent | Cumulative Percent |
| Valid | yes | | 3 | | 3.5 | | 3.5 | 3.5 |
|  | no | | 82 | | 96.5 | | 96.5 | 100.0 |
|  | Total | | 85 | | 100.0 | | 100.0 |  |


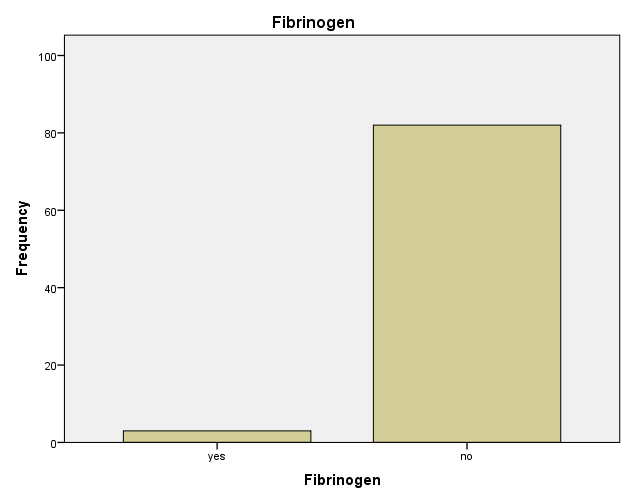


**Spleen size**

| **Statistics** | | | |  |  |  |  |  |
| --- | --- | --- | --- | --- | --- | --- | --- | --- |
| Spleen size | | | |  |  |  |  |  |
| N | | Valid | 85 |  |  |  |  |  |
|  |  | Missing | 0 |  |  |  |  |  |
| **Spleen size** | | | | | | | | |
|  | | | | | Frequency | Percent | Valid Percent | Cumulative Percent |
| Valid | Normal | | | | 64 | 75.3 | 75.3 | 75.3 |
|  | Splenomegaly | | | | 14 | 16.5 | 16.5 | 91.8 |
|  | Massive splenomegaly | | | | 7 | 8.2 | 8.2 | 100.0 |
|  | Total | | | | 85 | 100.0 | 100.0 |  |


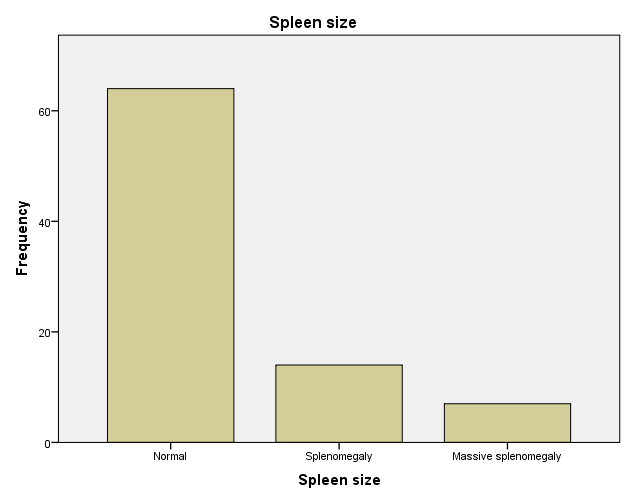


**Pathology**

| **Statistics** | | | | |  |  |  |  |
| --- | --- | --- | --- | --- | --- | --- | --- | --- |
| Pathology | | | | |  |  |  |  |
| N | | Valid | 85 | |  |  |  |  |
|  |  | Missing | 0 | |  |  |  |  |
| **Pathology** | | | | | | | | |
|  | | | | Frequency | | Percent | Valid Percent | Cumulative Percent |
| Valid | Benign | | | 72 | | 84.7 | 84.7 | 84.7 |
|  | Malignant | | | 13 | | 15.3 | 15.3 | 100.0 |
|  | Total | | | 85 | | 100.0 | 100.0 |  |


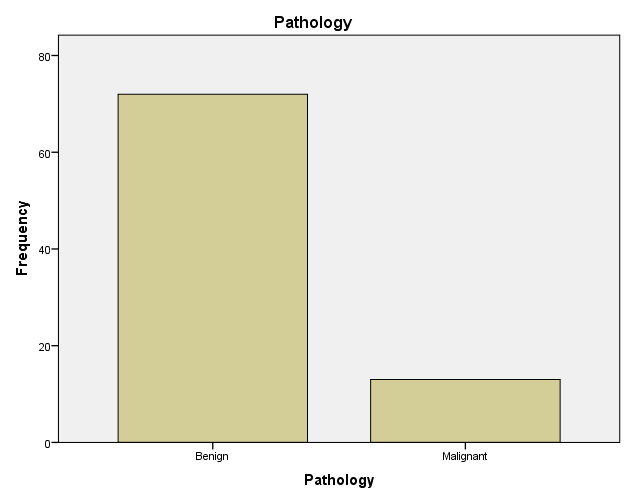


**Explore**

| **Case Processing Summary** | | | | | | | | | | |
| --- | --- | --- | --- | --- | --- | --- | --- | --- | --- | --- |
|  | Cases | | | | | | | | | |
|  | Valid | | Missing | | | | Total | | | |
|  | N | Percent | N | | Percent | | N | | Percent | |
| Hospital stay | 85 | 100.0% | 0 | | 0.0% | | 85 | | 100.0% | |
| **Descriptives** | | | | | | | | | |  |
|  | | | | | | Statistic | | Std. Error | |  |
| Hospital stay | Mean | | | | | 25.58 | | 5.033 | |  |
|  | 95% Confidence Interval for Mean | | | Lower Bound | | 15.57 | |  | |  |
|  |  |  |  | Upper Bound | | 35.59 | |  | |  |
|  | 5% Trimmed Mean | | | | | 18.23 | |  | |  |
|  | Median | | | | | 11.00 | |  | |  |
|  | Variance | | | | | 2153.461 | |  | |  |
|  | Std. Deviation | | | | | 46.405 | |  | |  |
|  | Minimum | | | | | 2 | |  | |  |
|  | Maximum | | | | | 365 | |  | |  |
|  | Range | | | | | 363 | |  | |  |
|  | Interquartile Range | | | | | 16 | |  | |  |
|  | Skewness | | | | | 5.259 | | .261 | |  |
|  | Kurtosis | | | | | 34.687 | | .517 | |  |

| **Percentiles** | | | | | | | | |
| --- | --- | --- | --- | --- | --- | --- | --- | --- |
|  | | Percentiles | | | | | | |
|  |  | 5 | 10 | 25 | 50 | 75 | 90 | 95 |
| Weighted Average(Definition 1) | Hospital stay | 4.00 | 5.00 | 7.00 | 11.00 | 22.50 | 57.60 | 104.50 |
| Tukey's Hinges | Hospital stay |  |  | 7.00 | 11.00 | 22.00 |  |  |

**Hospital stay**


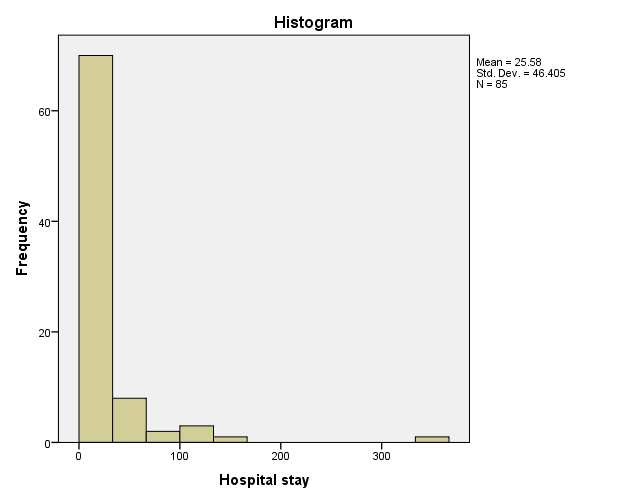


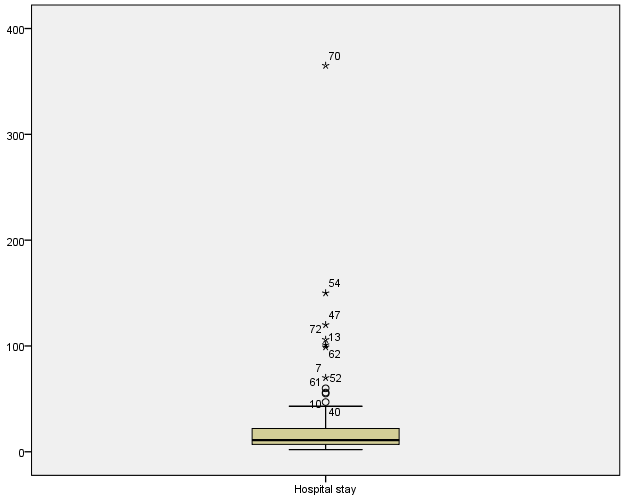


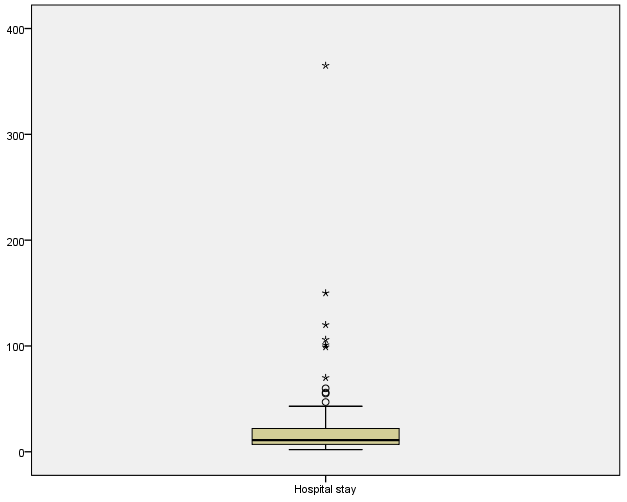


**Frequencies**

**Prophylaxis AC**

| **Statistics** | | | | | |  |  |  |
| --- | --- | --- | --- | --- | --- | --- | --- | --- |
| Prophylaxis AC | | | | | |  |  |  |
| N | | Valid | | 85 | |  |  |  |
|  |  | Missing | | 0 | |  |  |  |
| **Prophylaxis AC** | | | | | | | | |
|  | | | Frequency | | Percent | | Valid Percent | Cumulative Percent |
| Valid | yes | | 67 | | 78.8 | | 78.8 | 78.8 |
|  | no | | 18 | | 21.2 | | 21.2 | 100.0 |
|  | Total | | 85 | | 100.0 | | 100.0 |  |


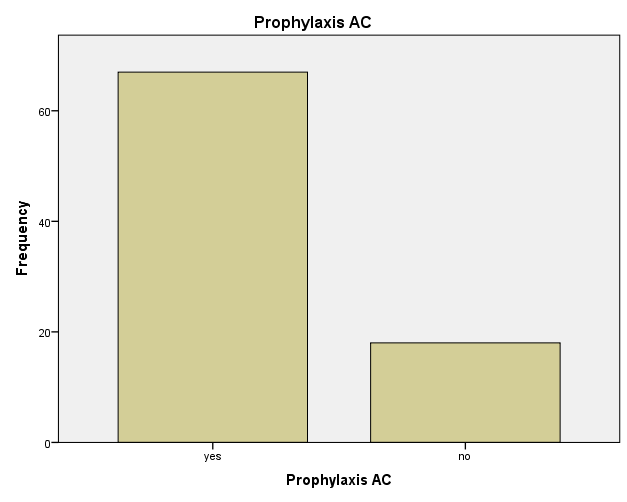


**Wound complication/Collection**

| **Statistics** | | | | | |  |  |  |
| --- | --- | --- | --- | --- | --- | --- | --- | --- |
| Wound complication/Collection | | | | | |  |  |  |
| N | | Valid | | 85 | |  |  |  |
|  |  | Missing | | 0 | |  |  |  |
| **Wound complication/Collection** | | | | | | | | |
|  | | | Frequency | | Percent | | Valid Percent | Cumulative Percent |
| Valid | yes | | 19 | | 22.4 | | 22.4 | 22.4 |
|  | no | | 66 | | 77.6 | | 77.6 | 100.0 |
|  | Total | | 85 | | 100.0 | | 100.0 |  |


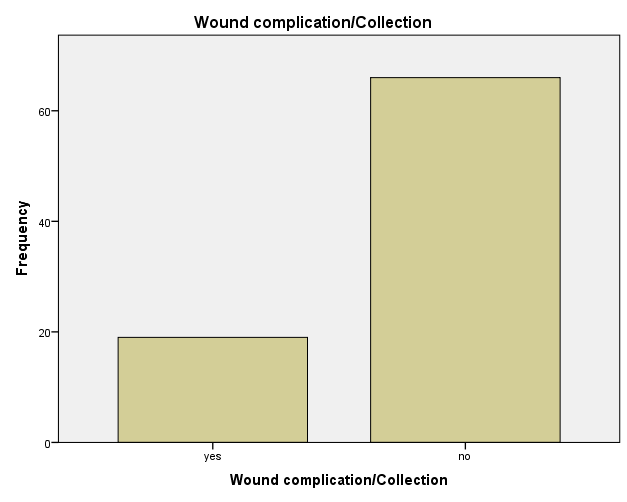


**Resurgery / Drainage**

| **Statistics** | | |
| --- | --- | --- |
| Resurgery / Drainage | | |
| N | Valid | 85 |
|  | Missing | 0 |

| **Resurgery / Drainage** | | | | | |
| --- | --- | --- | --- | --- | --- |
|  | | Frequency | Percent | Valid Percent | Cumulative Percent |
| Valid | Re-surgery | 8 | 9.4 | 9.4 | 9.4 |
|  | Drainage | 7 | 8.2 | 8.2 | 17.6 |
|  | None | 70 | 82.4 | 82.4 | 100.0 |
|  | Total | 85 | 100.0 | 100.0 |  |


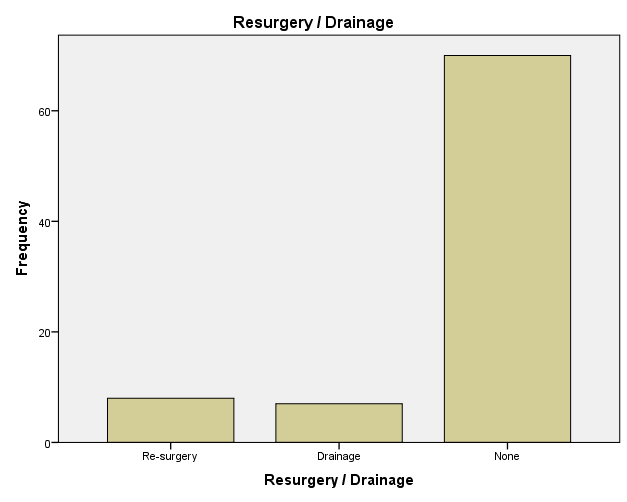


**Frequencies**

**Anticoagulation**

| **Statistics** | | |
| --- | --- | --- |
| Anticoagulation | | |
| N | Valid | 85 |
|  | Missing | 0 |

| **Anticoagulation** | | | | | |
| --- | --- | --- | --- | --- | --- |
|  | | Frequency | Percent | Valid Percent | Cumulative Percent |
| Valid | yes | 8 | 9.4 | 9.4 | 9.4 |
|  | no | 75 | 88.2 | 88.2 | 97.6 |
|  | N/A | 2 | 2.4 | 2.4 | 100.0 |
|  | Total | 85 | 100.0 | 100.0 |  |


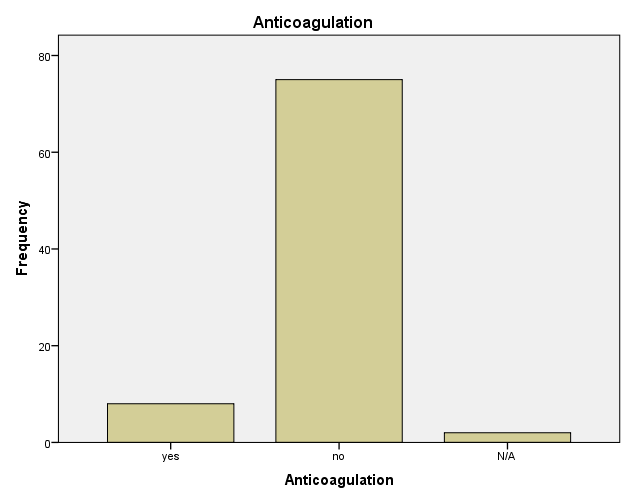


**Antiplatelets**

| **Statistics** | | | | | |  |  |  |
| --- | --- | --- | --- | --- | --- | --- | --- | --- |
| Antiplatelets | | | | | |  |  |  |
| N | | Valid | | 85 | |  |  |  |
|  |  | Missing | | 0 | |  |  |  |
| **Antiplatelets** | | | | | | | | |
|  | | | Frequency | | Percent | | Valid Percent | Cumulative Percent |
| Valid | yes | | 17 | | 20.0 | | 20.0 | 20.0 |
|  | no | | 68 | | 80.0 | | 80.0 | 100.0 |
|  | Total | | 85 | | 100.0 | | 100.0 |  |


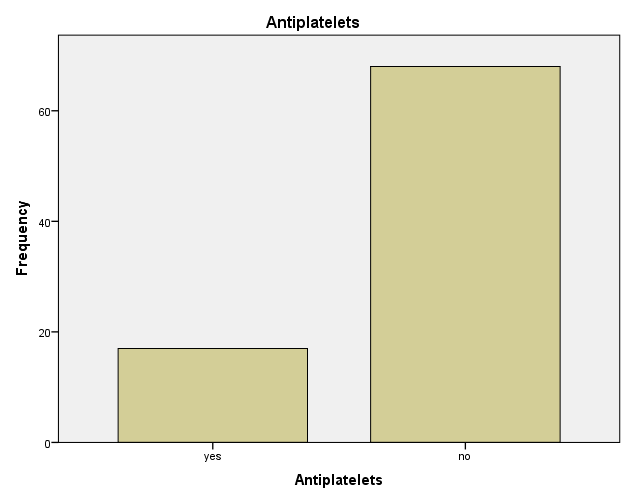


**Penicillin**

| **Statistics** | | |
| --- | --- | --- |
| Penicillin | | |
| N | Valid | 85 |
|  | Missing | 0 |

| **Penicillin** | | | | | |
| --- | --- | --- | --- | --- | --- |
|  | | Frequency | Percent | Valid Percent | Cumulative Percent |
| Valid | yes | 83 | 97.6 | 97.6 | 97.6 |
|  | no | 2 | 2.4 | 2.4 | 100.0 |
|  | Total | 85 | 100.0 | 100.0 |  |


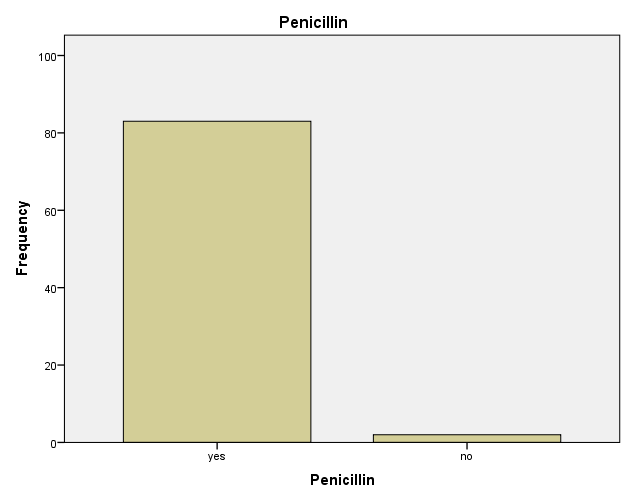


**Explore**

| **Case Processing Summary** | | | | | | |
| --- | --- | --- | --- | --- | --- | --- |
|  | Cases | | | | | |
|  | Valid | | Missing | | Total | |
|  | N | Percent | N | Percent | N | Percent |
| Post-op day | 85 | 100.0% | 0 | 0.0% | 85 | 100.0% |

| **Descriptives** | | | | |
| --- | --- | --- | --- | --- |
|  | | | Statistic | Std. Error |
| Post-op day | Mean | | 10.435 | .6239 |
|  | 95% Confidence Interval for Mean | Lower Bound | 9.195 |  |
|  |  | Upper Bound | 11.676 |  |
|  | 5% Trimmed Mean | | 10.170 |  |
|  | Median | | 10.000 |  |
|  | Variance | | 33.082 |  |
|  | Std. Deviation | | 5.7517 |  |
|  | Minimum | | .0 |  |
|  | Maximum | | 25.0 |  |
|  | Range | | 25.0 |  |
|  | Interquartile Range | | 7.5 |  |
|  | Skewness | | .553 | .261 |
|  | Kurtosis | | .030 | .517 |

| **Percentiles** | | | | | | | | |
| --- | --- | --- | --- | --- | --- | --- | --- | --- |
|  | | Percentiles | | | | | | |
|  |  | 5 | 10 | 25 | 50 | 75 | 90 | 95 |
| Weighted Average(Definition 1) | Post-op day | 2.000 | 2.600 | 6.500 | 10.000 | 14.000 | 18.400 | 22.700 |
| Tukey's Hinges | Post-op day |  |  | 7.000 | 10.000 | 14.000 |  |  |

**Post-op day**


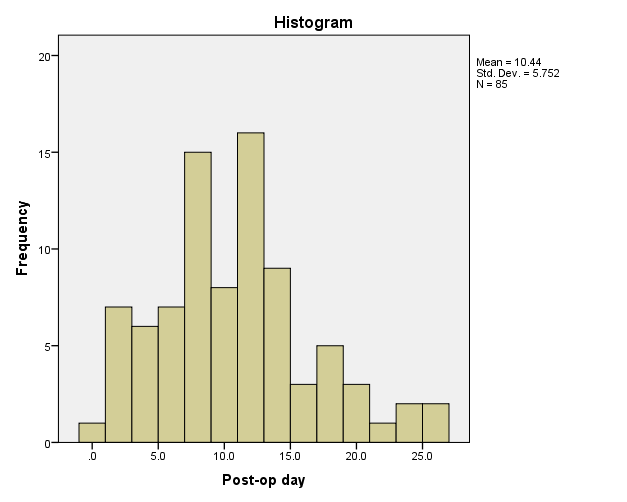


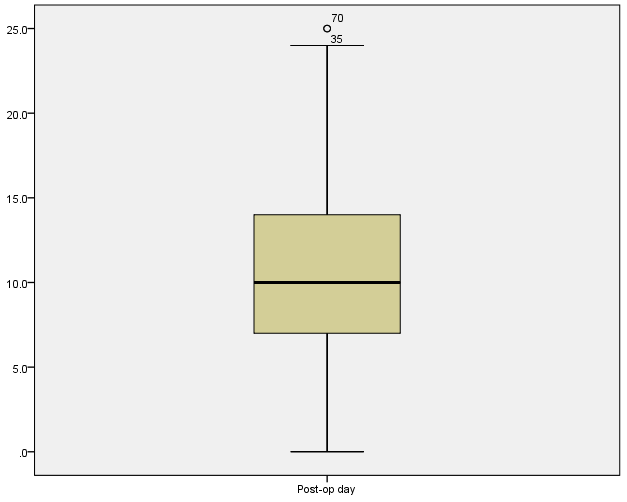


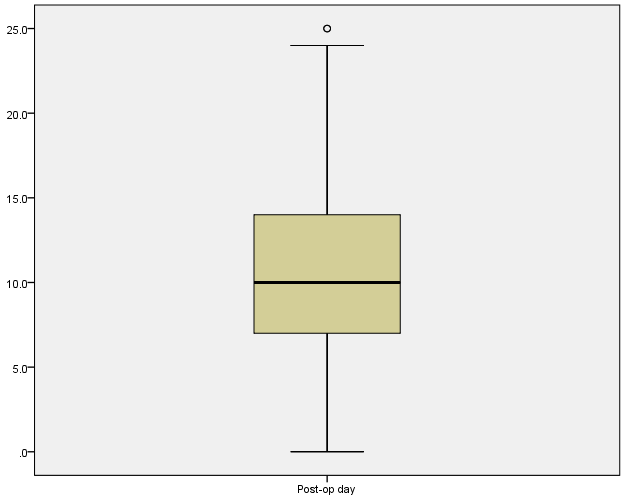


**Frequencies**

**Post -op PLT**

| **Statistics** | | | | |  |  |  |  |
| --- | --- | --- | --- | --- | --- | --- | --- | --- |
| Post -op PLT | | | | |  |  |  |  |
| N | | Valid | 85 | |  |  |  |  |
|  |  | Missing | 0 | |  |  |  |  |
| **Post -op PLT** | | | | | | | | |
|  | | | | Frequency | | Percent | Valid Percent | Cumulative Percent |
| Valid | <450 x 10^3 | | | 23 | | 27.1 | 27.1 | 27.1 |
|  | >450 x 10^3 | | | 41 | | 48.2 | 48.2 | 75.3 |
|  | >1000 x 10^3 | | | 21 | | 24.7 | 24.7 | 100.0 |
|  | Total | | | 85 | | 100.0 | 100.0 |  |


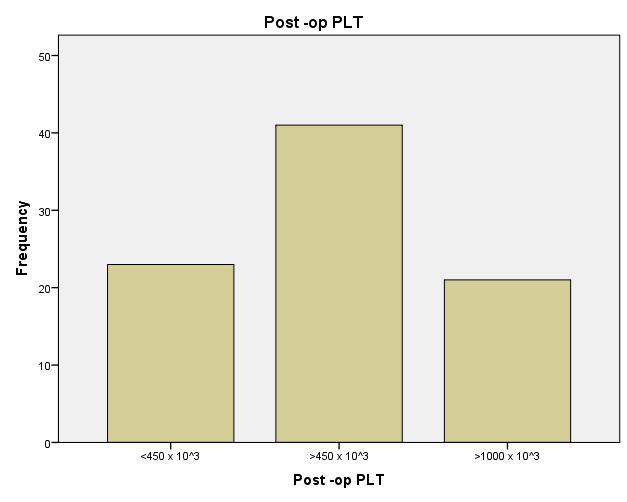


**VTE**

| **Statistics** | | | | | |  |  |  |
| --- | --- | --- | --- | --- | --- | --- | --- | --- |
| VTE | | | | | |  |  |  |
| N | | Valid | | 85 | |  |  |  |
|  |  | Missing | | 0 | |  |  |  |
| **VTE** | | | | | | | | |
|  | | | Frequency | | Percent | | Valid Percent | Cumulative Percent |
| Valid | yes | | 6 | | 7.1 | | 7.1 | 7.1 |
|  | no | | 79 | | 92.9 | | 92.9 | 100.0 |
|  | Total | | 85 | | 100.0 | | 100.0 |  |


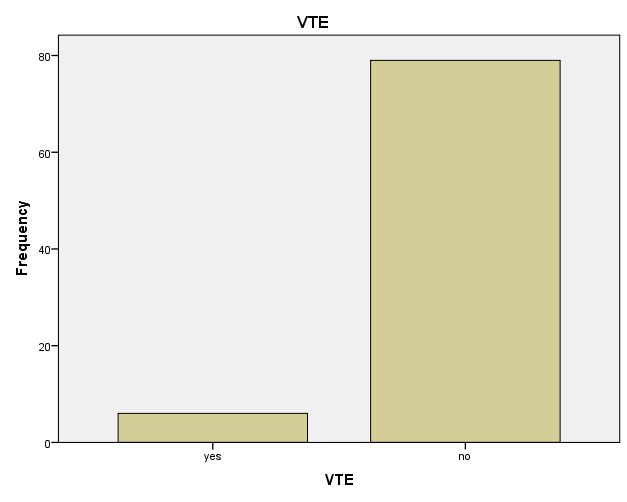


**Explore**

| **Case Processing Summary** | | | | | | |
| --- | --- | --- | --- | --- | --- | --- |
|  | Cases | | | | | |
|  | Valid | | Missing | | Total | |
|  | N | Percent | N | Percent | N | Percent |
| Charlson Comorbidity Index | 85 | 100.0% | 0 | 0.0% | 85 | 100.0% |

| **Descriptives** | | | | |
| --- | --- | --- | --- | --- |
|  | | | Statistic | Std. Error |
| Charlson Comorbidity Index | Mean | | 1.8941 | .23552 |
|  | 95% Confidence Interval for Mean | Lower Bound | 1.4258 |  |
|  |  | Upper Bound | 2.3625 |  |
|  | 5% Trimmed Mean | | 1.6895 |  |
|  | Median | | 1.0000 |  |
|  | Variance | | 4.715 |  |
|  | Std. Deviation | | 2.17137 |  |
|  | Minimum | | .00 |  |
|  | Maximum | | 9.00 |  |
|  | Range | | 9.00 |  |
|  | Interquartile Range | | 3.00 |  |
|  | Skewness | | 1.161 | .261 |
|  | Kurtosis | | .785 | .517 |

| **Percentiles** | | | | | | | | |
| --- | --- | --- | --- | --- | --- | --- | --- | --- |
|  | | Percentiles | | | | | | |
|  |  | 5 | 10 | 25 | 50 | 75 | 90 | 95 |
| Weighted Average(Definition 1) | Charlson Comorbidity Index | .0000 | .0000 | .0000 | 1.0000 | 3.0000 | 5.0000 | 7.0000 |
| Tukey's Hinges | Charlson Comorbidity Index |  |  | .0000 | 1.0000 | 3.0000 |  |  |

**Charlson Comorbidity Index**


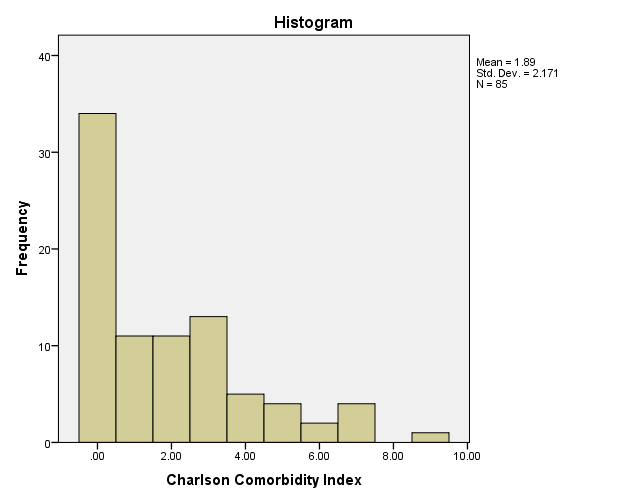


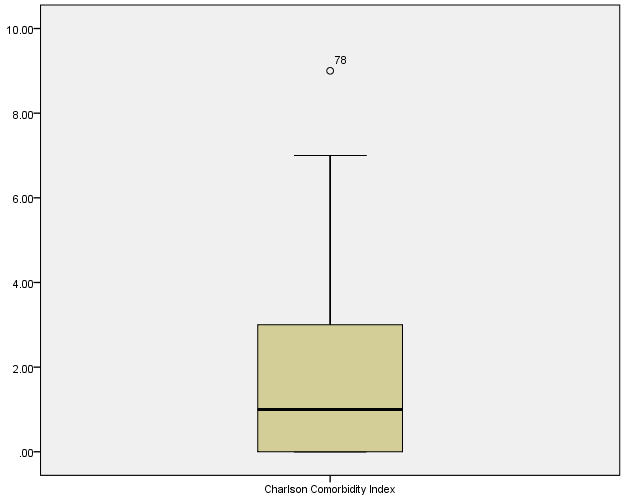


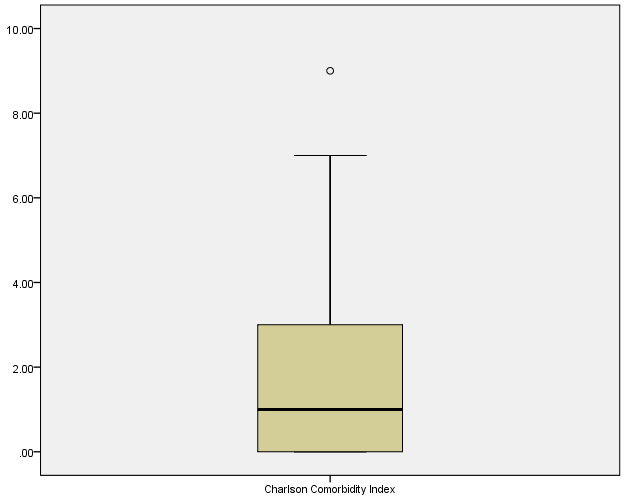

Supplement: Multimedia component 5 [file mmc5.docx]
